# Long non-coding RNA H19 contributes to apoptosis of hippocampal neurons by inhibiting let-7b in a rat model of temporal lobe epilepsy

**DOI:** 10.1038/s41419-018-0496-y

**Published:** 2018-05-23

**Authors:** Chun-Lei Han, Ming Ge, Yun-Peng Liu, Xue-Min Zhao, Kai-Liang Wang, Ning Chen, Wei Hu, Jian-Guo Zhang, Liang Li, Fan-Gang Meng

**Affiliations:** 1 Department of Functional Neurosurgery, Beijing Neurosurgical Institute, Capital Medical University, 100050 Beijing, China; 2 Beijing Key Laboratory of Neuromodulation, Beijing Municipal Science and Technology Commission, 100050 Beijing, China; 3 Department of Neurosurgery, Beijing Children’s Hospital, Capital Medical University, 100045 Beijing, China; 4 Department of Neurosurgery, Beijing Tiantan Hospital, Capital Medical University, 100050 Beijing, China; 5 Department of Neurology, University of Florida, Gainesville, Florida 32607 USA; 6 Department of Pathology, School of Basic Medical Sciences, Capital Medical University, 100069 Beijing, China

## Abstract

Temporal lobe epilepsy (TLE) is one of the most common types of intractable epilepsy, characterized by hippocampal neuron damage and hippocampal sclerosis. Long noncoding RNAs (lncRNAs) have been increasingly recognized as posttranscriptional regulators. However, their expression levels and functions in TLE remain largely unknown. In the present study, TLE rat model is used to explore the expression profiles of lncRNAs in the hippocampus of epileptic rats using microarray analysis. Our results demonstrate that H19 is the most pronouncedly differentiated lncRNA, significantly upregulated in the latent period of TLE. Moreover, the in vivo studies using gain- and loss-of-function approaches reveal that the overexpression of H19 aggravates SE-induced neuron apoptosis in the hippocampus, while inhibition of H19 protects the rats from SE-induced cellular injury. Finally, we show that H19 might function as a competing endogenous RNA to sponge microRNA let-7b in the regulation of cellular apoptosis. Overall, our study reveals a novel lncRNA H19-mediated mechanism in seizure-induced neural damage and provides a new target in developing lncRNA-based strategies to reduce seizure-induced brain injury.

## Introduction

Temporal lobe epilepsy (TLE) is the most common type of acquired epilepsy in adults, of which one-third of patients are refractory to medications^1^. Hippocampal neuron loss and hippocampal sclerosis are the common pathologic hallmarks of TLE in humans and animal models, which is involved in recurrent spontaneous seizures and cognitive impairment of TLE^2,3^. The development of TLE was characterized by a latent period following the initial precipitating injury (i.e., the acute period), such as status epilepticus (SE), till the appearance of recurrent seizures (i.e., the chronic period)^4^. During the latent period of the onset of spontaneous seizures, hippocampus undergoes a variety of pathological changes in cellular structure and function including neuronal loss and apoptosis, and the neuronal cell damage resulting from SE has been implicated as a causal factor in epileptogenesis^5^. However, current knowledge of the exact mechanisms by which neurons die following seizures remains quite limited.

The mammalian transcriptome comprises not only large numbers of protein-coding RNAs (messenger RNAs (mRNAs)), but also a large set of non-protein-coding transcripts that have structural, regulatory, or unknown functions^6^. In recent years, although studies of microRNAs (miRNA) have dominated the field of noncoding RNA biology in epileptogenesis^7^, the biological functions of long noncoding RNAs (lncRNAs) also attract increasing attention. LncRNAs, defined as noncoding RNAs of >200 nucleotides, are characterized by the complexity and diversity of their sequences and mechanisms of action^8^. A handful of studies have implicated that lncRNAs are involved in a variety of diseases, including tumor^9^ and nervous system diseases^10^. However, to date, few studies have explored the functions of lncRNAs in TLE.

H19 is the first identified lncRNA^11^. It is highly expressed during fetal life and strongly downregulated after birth, except for persistent expression in the adult skeletal muscle and heart^12^. The function of H19 is still controversial. Evidences have been presented that H19 can act as either an oncogene^13^ or tumor suppressor^14,15^. Apart from tumor-related properties, H19 is also involved in several other physiological conditions and non-cancerous disease states, such as cartilage degeneration in the course of osteoarthritis^16^, skeletal muscle differentiation and regeneration^17^, and glucose metabolism in muscle cells^18^. It is possible that H19 may play differential roles depending on tissue type and/or developmental stage of different pathological conditions^13^. In the central nervous system (CNS), H19 is significantly overexpressed in glioblastoma tissues and its expression level is associated with patient survival. Furthermore, increased H19 promotes the invasion, angiogenesis, stemness, and tumorigenicity of glioblastoma cells^19–21^. However, the biological role of H19 in non-neoplastic CNS diseases including epilepsy remains unknown.

In the present study, we explored the expression profiles of lncRNAs in the hippocampus of epileptic rat models and found that H19 was significantly upregulated in the latent period of epilepsy. In addition, using gain- and loss-of-function approaches in vivo, we found that H19 played an important role in hippocampal neuron apoptosis by acting as a competing endogenous RNA to target let-7b to regulate Casp3 expression.

## Results

### LncRNA and mRNA expression profiles in the hippocampus of rats after SE

**Fig. 1:**
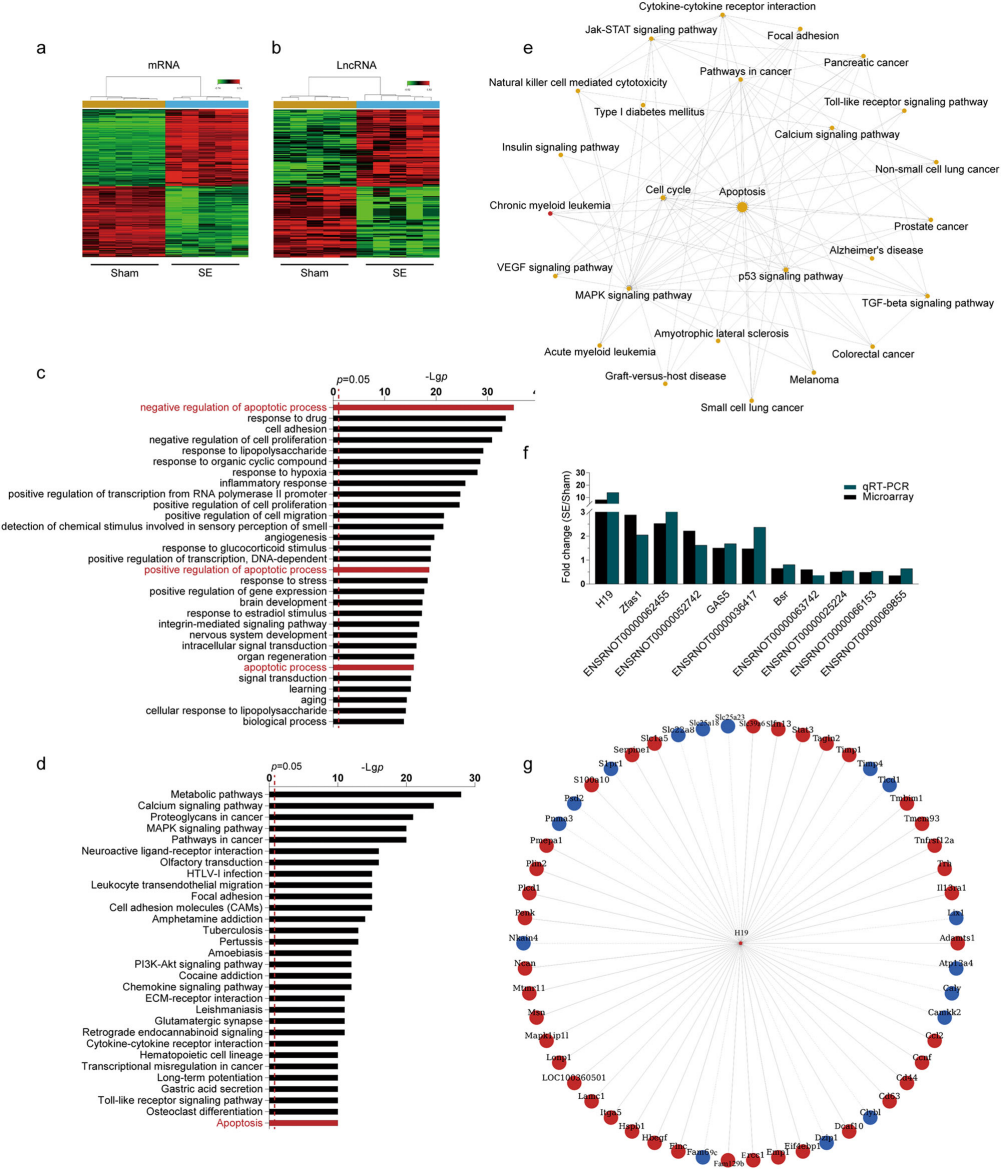
Differential expression of mRNAs and long noncoding RNAs (lncRNAs) in rat hippocampus after status epilepticus (SE). **a**,** b** Hierarchical clustering analysis of **a** 2612 mRNAs (>1.5-fold; *p* < 0.05) and **b** 313 lncRNAs (>1.2-fold; *p* < 0.05) (*n* = 5). Expression values represented in shades of red and green indicate expression levels above and below the median expression value across all samples. **c** Gene ontology (GO) and **d** pathway analysis of the differentially expressed genes. The first 30 GO names and pathways that exhibited significant differences after SE are listed. The GO terms or pathway related to apoptosis are indicated in red. The horizontal axis represents the gene expression levels after Log-normalized transformation and the vertical axis represents the GO names or pathways. The vertical dashed lines indicate the point of *p* *=* 0.05. The greater the −Log *p*-value, the higher the significance. **e** The sub-network of the significant pathways associated with SE centered on the apoptotic pathway. Pathways are represented by nodes, of which the red node represents a pathway in which all genes were upregulated and the yellow nodes represent pathways in which the genes were either upregulated or downregulated after SE. Lines indicate interactions between pathways where the pathways indicated by the arrowheads were regulated by the pathways indicated by the arrow tails. **f** The fold changes of the expression levels of 11 lncRNAs tested by microarray and qPCR, respectively (*n* = 5). **g** LncRNA H19 co-expression network. This co-expression network consists of H19 (center) and its 54 co-expression protein-coding genes. Protein-coding genes are represented by nodes, of which red nodes represent upregulated genes and blue nodes represent downregulated genes. The solid lines indicate positive correlations, and the dashed lines indicate negative correlations

To explore the expression profiles of lncRNAs and mRNAs related to epileptogenesis, hippocampi from epilepsy rat model at 1 day post-SE were used for microarray analysis. The threshold for differential expression was set as fold change >1.2 for lncRNAs and >1.5 for mRNAs. A total of 313 differentially expressed lncRNAs were found, of which 152 were downregulated and 161 were upregulated. Among them, H19 was the most upregulated lncRNA, increased by 8.1 times. Meanwhile, 2612 mRNAs were differentially expressed, of which 1361 were upregulated and 1251 were downregulated (Supplemental Material 1 and 2). Hierarchical clustering showed systematic variations in these differentially expressed mRNAs (Fig. 1a) and lncRNAs (Fig. 1b) between the sham and the epileptic rats. Function annotation and pathway analysis demonstrated that the differentially expressed genes were involved in many functions and pathways, including apoptosis (Fig. 1c–e). To validate the microarray analysis results, 11 lncRNAs (6 upregulated and 5 downregulated) were selected to test their expression levels by quantitative real-time polymerase chain reaction (qPCR). As shown in Fig. 1f, changes of the 11 selected lncRNAs were consistent with what had been found in the microarray, indicating that a set of lncRNAs, especially H19, might play important roles in epileptogenesis. Furthermore, gene co-expression network built according to the normalized signal intensity of specific expression genes showed that H19 was connected with 54 protein-coding genes (Fig. 1g).

### LncRNA H19 was highly expressed in the latent period of epilepsy

**Fig. 2:**
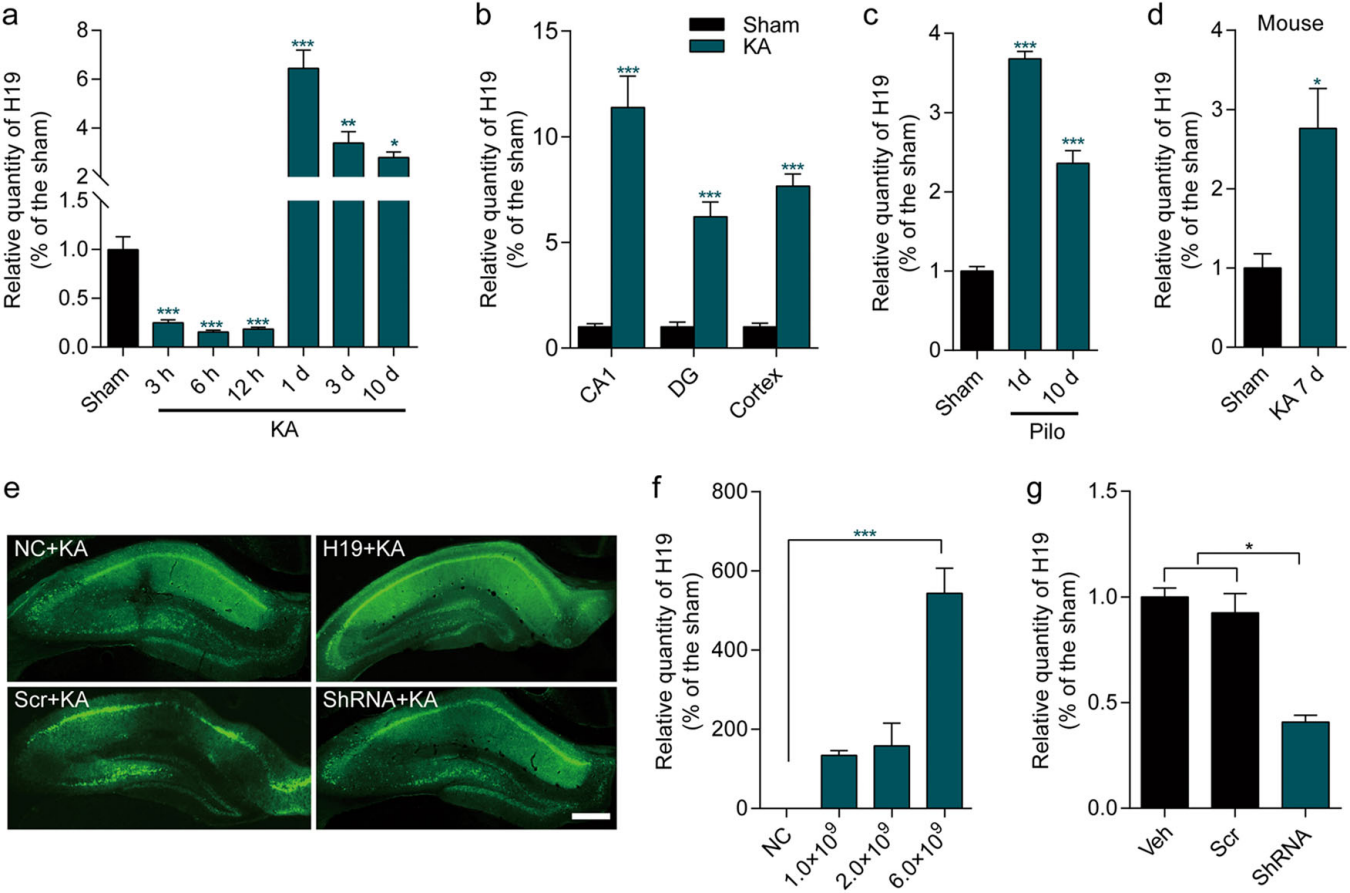
The expression pattern of lncRNA H19. The expression of lncRNA H19 was determined by qPCR in **a** CA3 subfield of hippocampus from epileptic rats induced by intra-amygdala kainic acid (KA) injection at 3, 6, 12 h and 1, 3, 10 days after KA injection (*n* = 5–7), in **b** CA1, DG subfield of hippocampus, and the frontoparietal cortex at 1 day after KA injection (*n* = 5–7), and **c** pilocarpine (Pilo) intraperitoneal injection at 1 and 10 days after Pilo injection (*n* = 3–4), as well as **d** in hippocampus from KA-induced epileptic mice at 7 days after KA injection (*n* = 4). **e** Representative fluorescence images of rat hippocampus injected with H19 overexpression and H19 knockdown vectors as well as their control vectors at 14 days after AAV vectors injection (scale bar = 400 μm). **f** Quantification of H19 expression examined by qPCR in CA3 subfield of the hippocampus with a stereotaxic injection of three different doses of AAV-H19 or negative control (NC) vectors for 14 days (*n* = 4). **g** The expression of H19 determined by qPCR in CA3 subfield of the hippocampus with a stereotaxic injection of vehicle (Veh), scramble (Scr), or short hairpin RNA targeting H19 (shRNA) AAV vectors for 14 days (*n* = 4-6). Relative H19 levels were normalized to GAPDH. All data are shown as mean ± s.e.m. *p < 0.05, **p < 0.01, ***p < 0.001 versus the sham group

To fully explore the H19 expression pattern in different periods of TLE, intra-amygdala injection of kainic acid (KA)-induced rat model was used for qPCR analyses of H19 expression. As shown in Fig. 2a, H19 expression in CA3 subfield of the hippocampus was significantly decreased in SE-experienced rats at 3, 6, and 12 h after KA injection; however, it was increased in the seizure-free latent period at 1, 3, and 10 days after KA injection. Consistent with what had been found in CA3 subfield, the same expression patterns were also observed in the CA1, DG subfield, and the frontoparietal cortex at 1 day after KA injection (Fig. 2b). In addition, analyses of H19 expression levels in pilocarpine (Pilo)-induced rat model, as well as KA-induced mouse model further confirmed the high expression of H19 in the latent period of epilepsy (Fig. 2c, d).

### H19 was involved in SE-induced neuronal loss and cellular apoptosis in the hippocampus

Next, the in vivo biological functions of H19 were investigated by H19 overexpression and knockdown using an adeno-associated virus (AAV) delivery system. The results of fluorescence observation on brain slices showed that the injected vectors had fused into the hippocampus (Fig. 2e). qPCR results confirmed that the application of AAV-H19 or AAV-shRNA resulted in significantly high H19 expression (Fig. 2f) or H19 reduction (Fig. 2g) in CA3 subfield of the hippocampus, respectively.

**Fig. 3:**
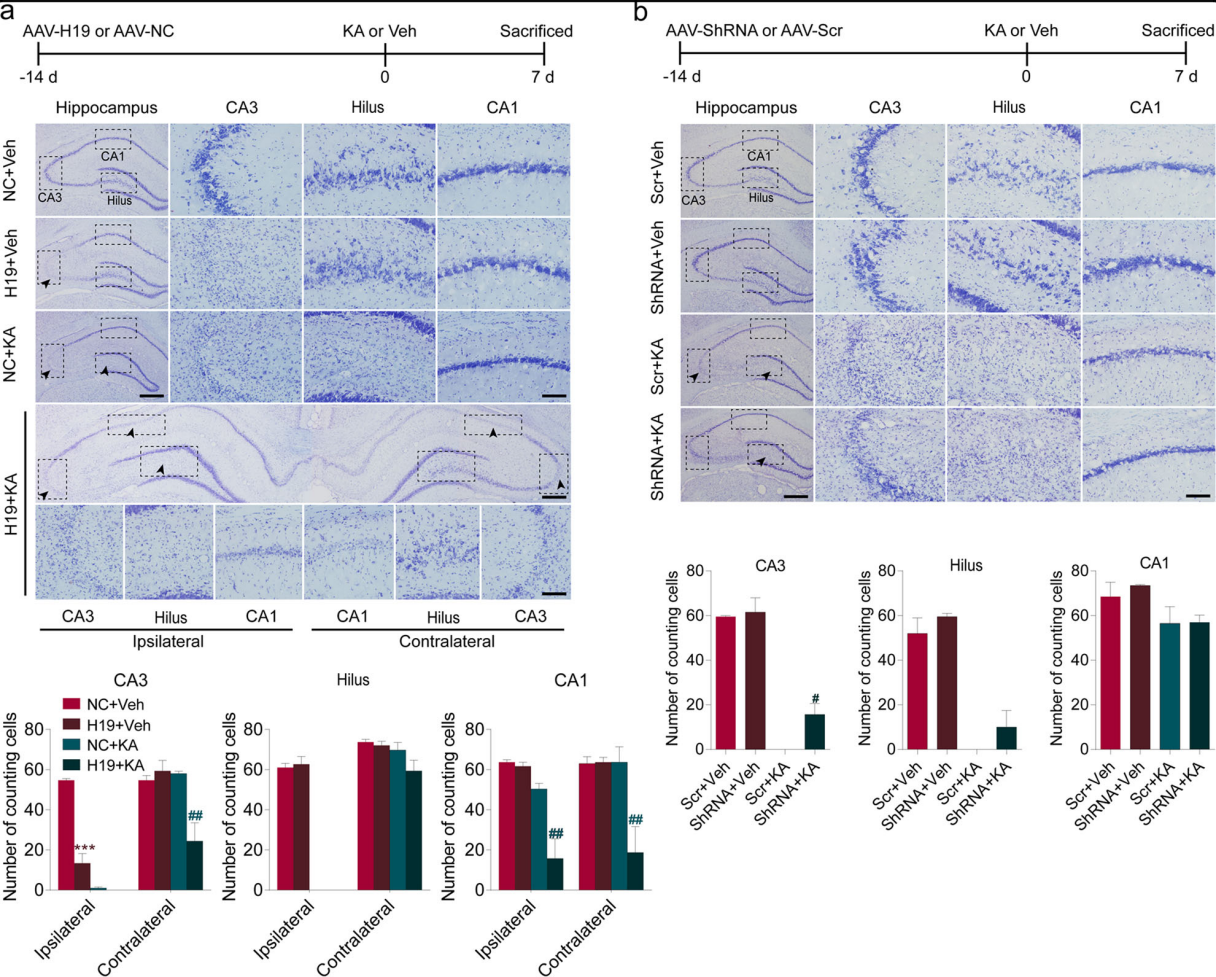
H19 exacerbated SE-induced hippocampal neuron death in vivo. **a**,** b** Top, the timeline showing the experimental design. Middle, representative Nissl staining photomicrographs of the ipsilateral hippocampus in response to the AAV vectors and/or KA injection from **a** H19 overexpression and **b** H19 knockdown rats with or without KA treatment for 7 days. The arrowheads indicate the damage areas (scale bar = 400 μm). The magnified images correspond to the labeled boxes in the far left panels (scale bar = 100 μm). The bottom panels are the counts of cells in the CA3, dentate hilus, and CA1 regions of hippocampus ipsilateral or contralateral to the AAV vectors and/or KA injection side (*n* = 3–5). All data are shown as mean ± s.e.m. ***p < 0.001 versus NC+Veh or Scr+Veh group; ^#^*p* < 0.05, ^##^*p* < 0.01, versus NC+KA or Scr+KA group

First, the effect of H19 on the hippocampal neurons from rats at 7 days after SE was investigated using Nissl staining and Fluoro-Jade C (FJC) staining. H19 overexpression alone without KA treatment resulted in neuron loss in CA3 area where AAV-H19 vectors were injected, and rats at 7 days after SE displayed neuron damage in the ipsilateral CA3 and dentate hilus areas of the hippocampus (Fig. 3a). Furthermore, AAV-H19 pretreatment aggravated SE-induced hippocampal neuron injury with a demonstration of expansion of damage area to ipsilateral CA1 area as well as contralateral CA1 and CA3 areas (Fig. 3a). In contrast, H19 knockdown showed no damage on the cells of CA3 area of normal rats and protected the cells in CA3 area in the rats at 7 days after SE (Fig. 3b). These morphological changes were further confirmed by FJC staining (Supplementary Figure 1). At 60 days after SE, rats of TLE exhibited ipsilateral hippocampal atrophy with the relative ratio of the ipsilateral hippocampus to the contralateral decreased to 61.6%. Moreover, H19 overexpression significantly exacerbated ipsilateral hippocampal atrophy with the relative ratio of the ipsilateral hippocampus to the contralateral further decreased to 50.4% (Supplementary Figure 2a). On the contrary, this hippocampal atrophy could be prevented by H19 knockdown with an increased relative ratio of the ipsilateral hippocampus to the contralateral from 60.4% to 75.8% (Supplementary Figure 2b).

**Fig. 4:**
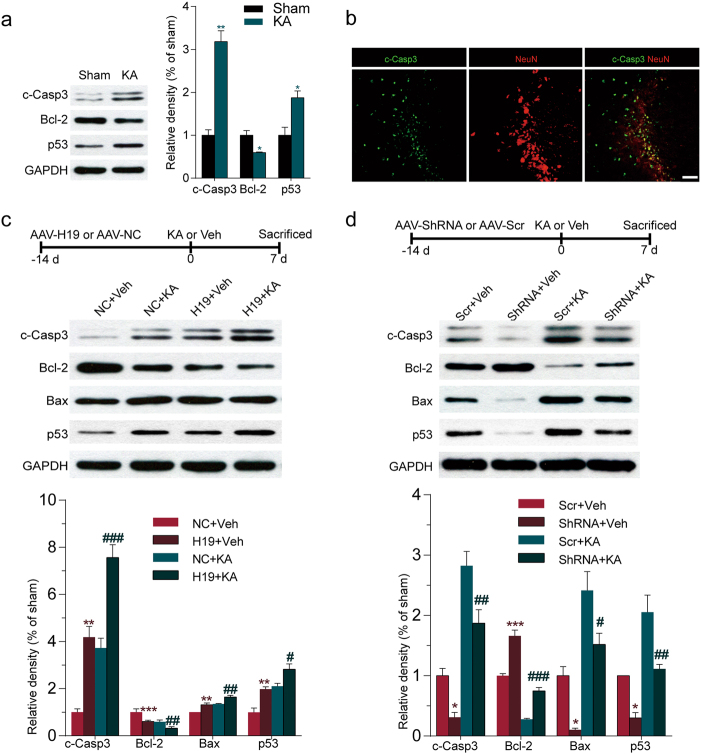
H19 promoted SE-induced hippocampal cell apoptosis in vivo. **a**,** b** The protein levels of c-Casp3, Bcl-2 and p53 in **a** hippocampus from KA-induced epileptic rats at 7 days after surgery (*n* = 3), as determined by western blot. **b**Representative photomicrographs of c-Casp3 and NeuN immunofluorescence in hippocampus from KA-induced epileptic rats at 7 days after KA injection (scale bars = 50 μm). **c**,** d** Top, the timeline showing the experimental design. Bottom, quantification of c-Casp3, Bcl-2, Bax, and p53 proteins in CA3 subfield of hippocampus from **c** H19 overexpression or **d** H19 knockdown rats with or without KA treatment for 7 days (*n* = 3–4). The blots were densitometrically quantified, and the data were normalized to GAPDH blot. All data are shown as mean ± s.e.m. *p < 0.05, **p < 0.01, ***p < 0.001 versus control (Con) or sham groups (sham, NC+Veh, or Scr+Veh). ^#^*p* < 0.05, ^##^*p* < 0.01, ^###^*p* < 0.001 versus NC+KA or Scr+KA group

Since the functional annotation and pathway analysis implied that apoptosis signaling pathway might be involved in SE-induced injury (Fig. 1c–e), proteins related to apoptosis were then examined. As shown in Fig. 4a, significantly elevated c-Casp3 and p53, as well as reduced Bcl-2 were also observed in CA3 subfield of the hippocampus from rats at 7 days after SE. Immunofluorescence analysis showed that c-Casp3 immunoreactive substances were located in neurons in CA3 area of the hippocampus (Fig. 4b). Experiments were performed to explore whether H19 influenced the expression levels of the apoptosis-related proteins. As shown in Fig. 4c, d, H19 overexpression increased the protein expression levels of c-Casp3, Bax, and p53 and reduced Bcl-2 expression in CA3 subfield of the hippocampus and H19 knockdown reversed these changes. Furthermore, H19 overexpression exacerbated the alterations in the expression of these proteins in the rats after SE, while H19 knockdown prevented SE-induced increase of c-Casp3, Bax, and p53 and promoted the anti-apoptotic protein Bcl-2 expression at 7 days after SE. All these changes were maintained to 60 days after SE (Supplementary Figure 3). The cellular apoptosis was further confirmed by terminal deoxinucleotidyl transferase-mediated dUTP-fluorescein nick end labeling staining (Supplementary Figure 4).

### H19 contributed to cellular apoptosis by targeting let-7b

**Fig. 5:**
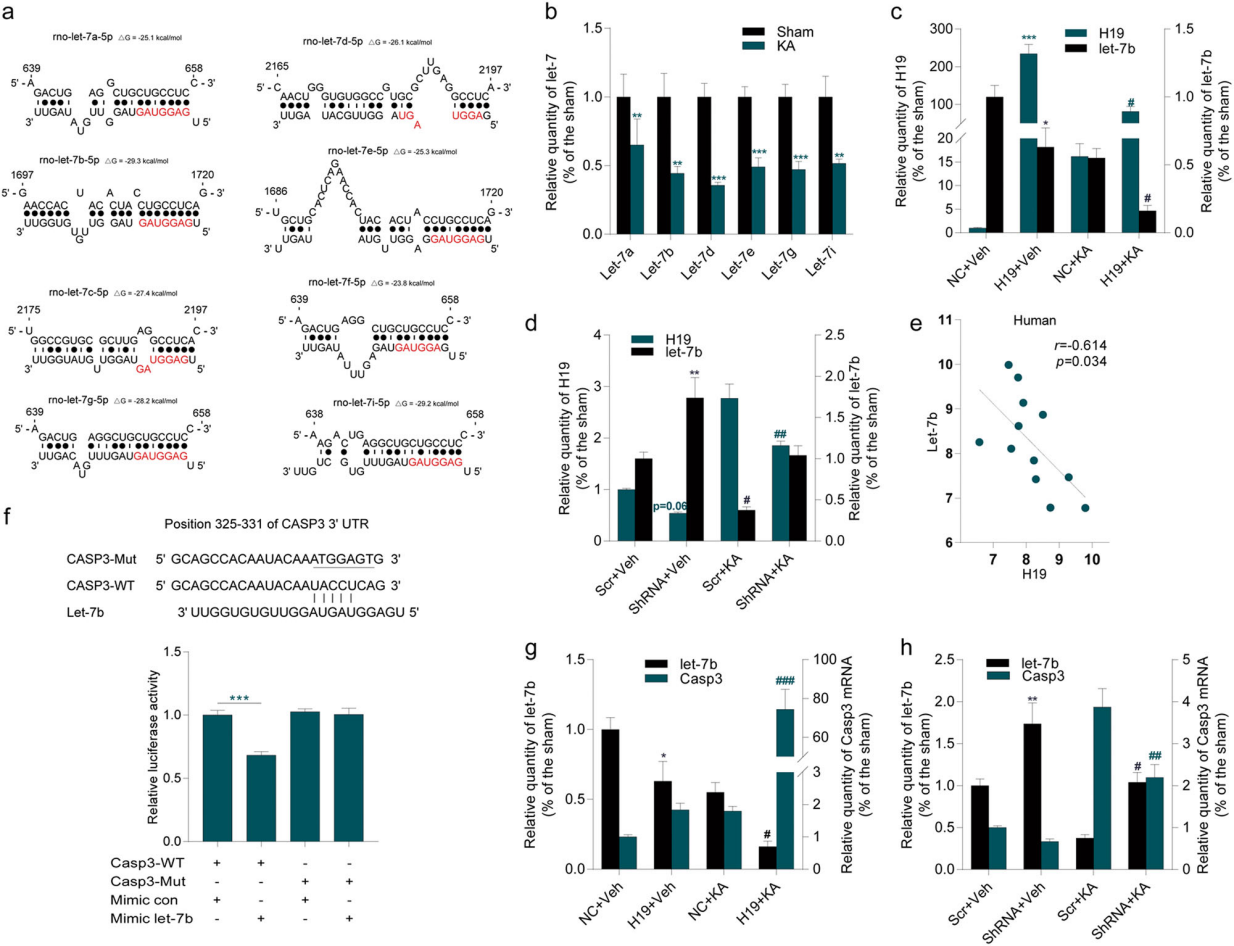
H19 might function by sponging let-7b targeting Casp3. **a** Bioinformatics-predicted binding sites for eight let-7 subtypes (let-7a, let-7b, let-7c, let-7d, let-7e, let-7f, let-7g, and let-7i) in rat H19. Let-7-binding sites on H19 were predicted using a web-based program RNAhybrid (http://bibiserv.techfak.uni-bielefeld.de/rnahybrid/). Nucleotides of the miRNA let-7 seed region (positions 2–8) are marked in red. **b** The expression levels of let-7 family members (let-7a, let-7b, let-7d, let-7e, let-7g, and let-7i) in CA3 subfield of hippocampus from sham-operated and KA-induced epileptic rats at 1 day after surgery as determined using qPCR (*n* = 7–8). **c**,** d** qPCR quantification of H19 and let-7b levels in CA3 subfield of hippocampus from H19 overexpression (**c**) and H19 knockdown (**d**) rats with or without KA treatment for 7 days (*n* = 8). **e** Correlations according to Pearson coefficient between H19 and let-7b levels in surgically resected hippocampal samples from patients with TLE (*n* = 12). **f** Top, complementarity between let-7b seed sequence and the 3′-UTR position of Casp3 of rat predicted by a computational and bioinformatics-based approach using TargetScan. Mutant Casp3 (Casp3-Mut) was generated by mutating the putative let-7b-binding site (underlined). Watson–Crick complementarity was connected by “|”. Bottom, luciferase reporter gene assay for interactions between let-7b and its binding sites or mutated binding sites in the 3′-UTR of the Casp3 mRNA in HEK293 cells. The relative luciferase activity was normalized against the firefly luciferase values shown as mean ± s.e.m (*n* = 3). **g**,** h** qPCR quantification of let-7b and Casp3 mRNA levels in CA3 subfield of hippocampus from **g** H19 overexpression (*n* = 8) and **h** H19 knockdown (*n* = 4–5) rats with or without KA treatment for 7 days. The relative H19 levels were normalized to GAPDH and the relative expression levels of let-7 family members were normalized to U6. All data are shown as mean ± s.e.m. *p < 0.05, **p < 0.01, ***p < 0.001 versus sham groups (sham, NC+Veh, or Scr+Veh). ^#^*p* < 0.05, ^##^*p* < 0.01, ^###^*p* < 0.001 versus NC+KA or Scr+KA group

Recent studies demonstrated that H19 could regulate gene expression by acting as competing endogenous RNA for miRNA let-7, resulting in the derepression of several protein-coding genes targeted by let-7^18,22,23^, as H19 harbors both canonical and non-canonical-binding sites for the let-7 family^23^. The bioinformatics analysis also revealed putative complementary sequences for let-7 family members (let-7a, let-7b, let-7d, let-7e, let-7g, and let-7i) in rat H19 (Fig. 5a). The expression levels of the let-7 family members were examined in CA3 subfield of the hippocampus from rats at 1 day after SE. Compared to the sham rats, the levels of all let-7 family members were significantly decreased (Fig. 5b). H19 overexpression and knockdown in the in vivo study further verified a strong negative correlation of H19 and let-7b expression in epileptic rats at 7 days (Fig. 5c, d) and 60 days (Supplementary Figures 5a–d) after SE. Correlation analysis also showed that H19 and let-7b had a negative correlation in hippocampal samples from patients with TLE (Fig. 5e).

According to the prediction of miRNA target gene, Casp3 is one of the potential downstream target genes of the let-7 family. The luciferase assay demonstrated that co-transfection of the let-7b mimic plasmid containing a sequence of position 325–331 in Casp3 3′-untranslated region (3′-UTR) consistently produced less luciferase activities by 31.72%. Mutation of the binding sites abolished the repressive effect of let-7b (Fig. 5f). H19 overexpression and knockdown in the in vivo study further verified the strong negative correlation of let-7b and Casp3 protein levels in epileptic rats at 7 days (Fig. 5g, h) and 60 days after SE (Supplementary Figures 5e, f). These data suggested that H19 played an important role in SE-induced hippocampal neuron damage by acting as a competing endogenous RNA to target let-7b to regulate Casp3 expression.

## Discussion

To our knowledge, there has been only one reported study that focuses on the lncRNA expression profiles in the chronic period of TLE presenting with spontaneous recurrent seizures^24^. The authors identified hundreds of dysregulated lncRNAs in the whole brain of Pilo and KA mouse models using microarray analysis, as well as lncRNA-associated genes involving embryonic appendage morphogenesis and neuron differentiation using Gene ontology analysis. Unfortunately, it did not perform further functional analyses. In addition, we were not sure whether lncRNA H19 was changed or not in that study, as the authors only listed partially dysregulated lncRNAs in their article and supplementary material^24^. The latent period is an important stage for epileptogenesis as a variety of molecular and cellular changes occur in this stage (especially in the hippocampus), finally transforming normal brain to an epileptic one. Here, for the first time, we report the aberrant lncRNA profiles in the hippocampus of epileptic rats in the latent period of TLE, of which H19 is the most significantly differentiated RNA. It is reported that H19 is highly expressed during embryogenesis to promote differentiation^25^, but its expression is shut off in most tissues postnatally^26^. In the present study, our results show that H19 is upregulated for a long time in the latent period of TLE, implying that H19 might play important roles in epileptogenesis.

It has been suggested that hypoxia can inhibit the expression of H19^27^. It is well known that epileptic seizures, including SE and spontaneous recurrent seizures, result in severe hypoxic insult in the brain, which could last at least 1 h after seizure termination^28,29^. The present study shows that H19 is downregulated in the seizure-experienced rats at the acute periods of TLE, indicating that acute cerebral hypoxia caused by seizures may inhibit H19 expression. However, the expression of H19 is significantly elevated in the seizure-free latent period of TLE in this study. Our in vitro studies show that H19 expression is repressed under hypoxia, while reoxygenation restores H19 expression (data not shown). It is possible that reoxygenation following the hypoxia induced by the epileptic seizures might lead to H19 elevation in the latent period. Studies have reported that many factors such as cytokines, growth factors, and tissue repair factors^30^, as well as transcription factors such as hypoxia-inducible factor-1a (HIF-1a)^31^ and c-Myc^32,33^, can promote H19 expression. Meanwhile, most of these factors such as HIF-1α^34,33^ and c-Myc (data not shown) increase in the latent period of TLE. Therefore, it is possible that SE-induced activation of these substances might participate in the upregulation of H19 in the latent period of TLE. We further speculated that, in the chronic period of TLE, the hypoxia-reoxygenation induced by repeated spontaneous recurrent seizures leads to H19 expression change, thus exacerbate the hippocampal damage. However, the detailed mechanisms need to be determined in future studies.

The hippocampus is highly susceptible to neuropathological injuries following SE. As seizure discharge propagates from the injection site (amygdaloid) to hippocampus through the perforant pathway, pyramidal cells of CA3 and hilus areas are usually damaged ahead of CA1 area in the early post-SE phase. Over time, the extent of the lesion expanded to CA1 as epilepsy progresses, and finally, the hippocampus will degenerate completely within 2 weeks to 1 month after SE^35^. In the present study, we found that the hippocampus damage is usually restricted to the ipsilateral CA3 and hilus areas at 7 days after SE, while the contralateral hippocampus was usually not involved. However, H19 overexpression expanded the extent of damage induced by SE from ipsilateral CA3 and hilus areas to the CA1 area, even to the contralateral CA1, CA3, and hilus areas. On the other hand, H19 knockdown preserved SE-induced neural damage within the hippocampus especially in the CA3 area and prevent the damage extension to the contralateral hippocampus. FJC stain, a method to stain all degenerating neurons, confirmed the result found by Nissl stain in this study. These results indicate that H19 is involved in hippocampal neural damage in the rats of TLE.

Apoptosis, one of the key molecular mechanisms underlying cell death following SE, is a form of programmed cell death used to dispose of unwanted or damaged cells in a controlled manner. Caspases and Bcl-2 family proteins, comprising both pro-apoptotic members (such as Bax) and anti-apoptotic members (such as Bcl-2), are key protein modulators of the apoptotic response^36^. Alterations of apoptosis-associated signaling pathways are widely reported in TLE tissue and animal TLE models. Consistent with previous studies^36^, we also found elevated pro-apoptotic proteins and decreased anti-apoptotic proteins in the hippocampus from the epileptic rat. Here we demonstrated that H19 promoted the apoptosis of neurons in the hippocampus not only in the epileptic state but also in the normal physiological state, indicating the pro-apoptosis functional of H19. This is also consistent with our previous research, showing that high-throughput microarray analysis and bioinformatics analysis predicts the diverse functions of H19, including cell apoptosis^37^.

The mechanisms of downstream regulation of lncRNA are multifaceted and complicated. LncRNA can either positively or negatively regulate gene expression in various biological contexts through diverse mechanisms at the transcriptional, translational, or mRNA stability levels^38^. There are two regulatory types of H19: on one hand, it can encode miR-675 from its first exon, inhibiting its target genes^17,39^; on the other hand, it can function as a ceRNA to bind a series of miRNAs including let-7b^18,22,23^, derepressing their targets. In this study, miR-675 (both miR-675-3p and miR-675-5p) levels in the hippocampus of the rat after SE was not changed (data not shown). The mechanisms of downstream regulation of H19 are complicated and H19 may play differential roles depending on tissue type and/or developmental stage of different pathological conditions^13^. The previous studies reveal that H19 enhances tumorigenesis, metastasis, and invasion of different kinds of tumor cells through encoding miRNA-675, such as pancreatic ductal adenocarcinoma^40^, breast cancer^41,42^, gastric cancer^43^, and glioma^19^. However, the present results with no changes of miR-675 after SE suggest that H19 might not act by encoding miR-675 during epileptogenesis in TLE. Bioinformatics analysis and luciferase assays demonstrate that vertebrate H19 harbors binding sites for the miRNA let-7 family and can bind to let-7 directly to modulate its availability^23^ The results from the present study are consistent with the reports. H19 regulates gene expression by acting as a competing endogenous RNA for let-7 in SE-induced neural damage during epileptogenesis.

Collectively, there are hundreds of dysregulated lncRNAs including H19 after SE. The upregulation of H19 in the latent period of epilepsy is involved in SE-induced neuronal damage by functioning as a competing endogenous RNA to sponge miRNA let-7b in the regulation of cellular apoptosis. These findings contribute to our understanding of the molecular mechanisms of seizure-induced neural death and may offer a new therapeutic target to interfere with seizure-induced brain damage. However, the upstream and downstream regulation mechanisms of H19 are complex. Extensive studies are required to reveal the role of H19 in the epileptogenesis in the future.

## Materials and methods

### Animals

Male Sprague-Dawley rats weighing 200–220 g were obtained from Vital-River Experimental Animal Technology, Co., Ltd. (Beijing, China). Animals were housed and maintained in a temperature-controlled room with a 12-h light–dark cycle and free access to standard food and water. All animal experimental procedures were in compliance with the Chinese Animal Welfare Act, the Guidance for Animal Experimentation of Capital Medical University, and Beijing Guidelines for the Care and Use of Laboratory Animals. The study was approved by the Ethics Committee of Beijing Neurosurgical Institute, Capital Medical University (Process NO. 201402019). Efforts were made to minimize the number of animals used and their suffering.

### Epilepsy models

KA-induced epilepsy rat model was established by intra-amygdala micro-injection of KA as described previously^44^. After anesthesia (10% chloral hydrate, 0.003 ml/g, intraperitoneal (i.p.)), the animals were placed in a stereotaxic apparatus (David Kopf Instruments, USA). A total of 0.7 or 0.3 μl KA (1 μg/μl, Sigma-Aldrich, USA) was injected into amygdala (2.76 mm posterior to bregma, 4.5 mm lateral from midline, 8.6 mm ventral to bregma) of rats according to the brain atlas of Paxinos and Watson^45^. The animals that received intra-amygdala saline injections were used as sham-operated controls. The injection was conducted with a 1 µl stepper-motorized microsyringe (Pigeon, Shanghai, China) at a rate of 0.2 μl/min. The needle was withdrawn over a course of 5 min. For mice, the coordinates were: 0.94 mm posterior to bregma, 2.75 mm lateral from midline and 4.75 mm ventral, and 0.3 μl KA was injected. Pilo-induced epilepsy rat model was established by lithium plus Pilo i.p. injection as described previously^46^. An aqueous solution of lithium chloride (3 eEq/kg, i.p.; Sigma-Aldrich) was injected 18 h prior to the administration of Pilo (30 mg/kg, i.p.; Sigma-Aldrich). Rats were pretreated with scopolamine methyl bromide (1 mg/kg, i.p.; Sigma-Aldrich) 30 min prior to Pilo injection to reduce its peripheral effects. Development of seizures was evaluated by Racine’s scale assessment^47^. Seizures were terminated with chloral hydrate (10%, 0.003 ml/g, i.p.) when rats experienced stage four or greater seizures for 90 min. The rats that received an equal amount of saline were used as the sham-operated controls.

### Microarray and data analysis

Genome-wide expression profiling analysis was performed by Genminix Informatics Ltd., Co. using GeneChip Rat Gene 2.0 ST Array (Affymetrix, USA). Briefly, total RNA was separately extracted from the 10 individual samples using the RNeasyMini Kit (QIAGEN). Double-stranded complementary DNA (cDNA) was then synthesized, labeled, and hybridized to the gene chip. After hybridization and washing, the slides were scanned with the GeneChip GCOS Software (Affymetrix, USA). Raw data extraction and subsequent data processing were performed using the Affymetrix GeneChip Operating Software (Affymetrix, USA). The random-variance model *t*-test was applied to filter differentially expressed genes. After significance analysis and false discovery rate (FDR) analysis, differentially expressed genes were selected according to their *p*-value threshold and fold change. The threshold set for upregulated and downregulated mRNAs was a fold change >1.5 and a *p*-value <0.05, and for lncRNAs was a fold change >1.2 and a *p-*value <0.05. Pathway analysis was applied to find out significant pathways of the differential expression genes according to the KEGG database. Two-side Fisher’s exact test and χ^2^ test were used to classify the significant pathways. The FDR was calculated to correct the *p*-value and the threshold of significance was defined by *p-*value <0.05. The interaction net of the significant pathways was built according to the interaction among pathways of the KEGG database to find the interaction among the significant pathways directly and systemically. Gene co-expression network was built according to the normalized signal intensity of differentially expressed genes.

### Stereotaxic injection of the AAV vectors

The AAV9 vectors carrying H19 (AAV9-H19) or short hairpin RNA targeting H19 (AAV9-shRNA) were constructed by GeneChem Co., Ltd. (Shanghai, China). For AAV9-H19 packaging, a CMV-betaGlobin-MCS-SV40 PolyA vector was used. The titer of these AAV vectors was 3.5 × 10^12^ vg/ml. The empty AAV vectors coding EGFP (AAV-NC) were used as the control. For AAV9-shRNA packaging, the sequence of the shRNA primers was 5′-GTGCAGGTAGAGCGAGGTAAA-3′ and the scramble sequence (AAV-Scr) was 5′-TTCTCCGAACGTGTCACGT-3′. These sequences were inserted into the pAAV-U6-shRNA-Ubi-eGFP-3Flag vector. The titer of these AAV vectors was 1.0 × 10^13^ vg/ml. AAV vectors were injected into both the right dorsal and ventral hippocampus. The titers used were 1.0 × 10^12^ for AAV9-H19 and 4.0 × 10^12^ for AAV9-ShRNA. A total of 6 μl AAV was infused through a microsyringe at a speed of 0.2 μl/min into the right dorsal hippocampus (3.12 mm posterior to bregma, 3.0 mm lateral from midline, 3.4 mm ventral to bregma) and the ventral hippocampus (5.04 mm posterior to bregma, 5.0 mm lateral from midline, 6.4 mm ventral to bregma; 3 μl at each location in the dorsal–ventral plane).^45^ To prevent backflow of viral particles, the pipette was left in place for an additional 5 min after injection.

### Human sample and ethics statement

Surgically resected hippocampi used in this study were obtained from patients with intractable TLE, who underwent surgical treatment in Beijing Tiantan Hospital. All patients were diagnosed with pharmacoresistant TLE with hippocampal sclerosis according to high-resonance magnetic resonance imaging measurements; interictal fluorodeoxyglucose–positron emission tomography, electroencephalography (EEG), or video-EEG recording; and histopathologic examination. Surgically resected tissues from TLE patients were stored in liquid nitrogen until further analysis. All experimental protocols in this study were approved by the Ethics Committee on Human Research at Capital Medical University. Written informed consent was obtained from each patient for the use of brain tissues for research purposes.

### Nissl staining

Coronal sections (25 μm) from levels of the dorsal hippocampus (2.50–3.50 mm posterior to bregma) were analyzed. Tissue sections were stained with Cresyl violet (Beyotime Institute of Biotechnology, Shanghai, China) in accordance with the manufacturer’s protocol. Brain sections were stained with 1% toluidine blue for 10 min. The slides were then rinsed in distilled water, dehydrated in a series of gradient ethanol (70, 80, 90, and 100 %), cleared in xylene, and coverslipped with neutral balsam. Every fifth coronary section was collected and a total of three sections from each animal were used for quantification. The total cell number in CA3, dentate hilus, and CA1 areas of the hippocampus were counted from three non-overlapping 400× fields of each section (Olympus BX41, Olympus Optical Co. Ltd., Japan) using a computer-assisted image analysis system (Leica Qwin Analysis software V2.8). The cells in well-delimited form with a distinct nucleus were counted. Neurons with shrunken cell body or surrounding empty spaces were considered destined to die and excluded from the counting. The cross-sectional area of the hippocampus was measured using the ImageJ software (US National Institutes of Health). All procedures were conducted by a pathologist, who was blind to the grouping.

### FJC staining

For detection of neurodegeneration, sections were stained using FJC staining^48^. Briefly, sections were immersed in 80% ethanol containing 1% sodium hydroxide for 5 min followed by 70% ethanol and distilled water for 2 min each and then treated with 0.06% potassium permanganate solution for 10 min. After rinsing in distilled water for 2 min, sections were incubated with 0.001% FJC (Millipore, Bedford, MA, USA) in 0.1% acetic acid for 30 min. The slides were rinsed in distilled water three times for 1 min each and air dried. At last, the slides were cleared in xylene for 5 min and then coverslipped with neutral balsam in xylene mounting medium.

### Immunohistochemistry

Frozen sections were dried, washed, permeabilized, and blocked in 5% goat serum and then incubated overnight with antibodies against NeuN (1:500; Abcam/ab177487, 1:200; Abcam/ab104224) and cleaved-Caspase3 (1:500; CST/9664#). Sections were then washed and incubated with secondary goat antibodies conjugated with Alexa Fluor 594 (for NeuN) or Alexa Fluor 488 (for cleaved-Caspase3) (BioSciences Ltd). All images were captured using a Leica inverted fluorescence microscope (Olympus, Japan).

### Quantitative real-time PCR

Rats or mice were deeply anesthetized with 10% chloral hydrate (500 mg/kg, i.p.) and decapitated. Hippocampus was separated and further subdivided to obtain the separate CA1, CA3, or DG-enriched portion using a microdissection procedure described previously^49^. Total RNAs were extracted using the Ultrapure RNA Kit (CWbio Co. Ltd, Beijing, China) according to the manufacturer’s recommendations. Reverse transcription was obtained using the HiFi-MMLV cDNA First Strand Synthesis Kit according to the manufacturer’s instructions (CWbio Co. Ltd, Beijing, China). qPCR was performed with UltraSYBR Mixture (CWbio Co. Ltd, Beijing, China) in a 20 µl system containing 10 µl of UltraSYBR Mixture, 0.4 µl of each primer (10 µM), 2 µl of cDNA sample, and 7.2 µl of dH_2_O using ABI Prism 7500 sequence detection system (Applied Biosystems) at the conditions of initial denaturation at 95°C for 10 min followed by 40 cycles of 15 s at 95°C and 60 s at 60°C. The primer sequences were 5′-GATGGAGAGGACAGAAGGACAGT-3′, and 5′-GAGAGCAGCAGAGATGTGTTAGC-3′ for rat H19; 5′-CCTCAAGATGAAAGAAATGGTGCTA-3′ and 5′-TCAGAACGAGACGGACTTAAAGAA-3′ for mouse H19; 5′-TGCTGCACTTTACAACCACTG-3′ and 5′-ATGGTGTCTTTGATGTTGGGC-3′ for Human H19; 5′-CCCAGCGAGACTCTGTGCGGA-3′ and 5′-GGAAGTACGGCCTGAGAGGTA-3′ for IGF2; 5′-ACTGGAAAGCCGAAACTCTTCATCA-3′ and 5′-GGAAGTCGGCCTCCACTGGTATC-3′ for Casp3; 5′-GCTGGTGCCGAGTATGTT-3′ and 5′-CAGAAGGTGCGGAGATGA-3′ for rat glyceraldehyde 3-phosphate dehydrogenase (GAPDH); and 5′-CTGGGCTACACTGAGCACC-3′ and 5′-AAGTGGTCGTTGAGGGCAATG-3′ for human GAPDH. Each sample was determined in triplicate. The PCR products were confirmed by melting curve analysis. Relative expression levels were normalized to GAPDH or U6 using the 2^−ΔΔCt^ method.

### Western blot analysis

Samples were harvested and homogenized. A standard procedure was carried out as described previously^50^. The primary antibodies used were rabbit anti-cleaved-Caspase3 monoclonal antibody (1:500; CST/9664#), rabbit anti-Bcl-2 polyclonal antibody (1:100; Santa/Sc-492), rabbit anti-Bax monoclonal antibody (1:500; Abcam/ab32503), and mouse anti-p53 monoclonal antibody (1:500; CST/2524#). The rabbit anti-GAPDH monoclonal antibody (1:3000; Abcam/ab181602) was used as a control. The quantification of the blots was carried out using Epson V330 Photo scanner (Seiko Epson Co., Nagano, Japan) and analyzed with the Quantity One software (Bio-Rad, USA).

### Dual luciferase reporter assays

The luciferase reporter plasmid pmiR-RB-REPORTTM (RiboBio, Guangzhou, China) encoding both renialla luciferase (hRluc) and the control firefly luciferase (hluc+) was used for all assays. The 3′-UTR sequence of Casp3 was obtained from the NCBI (gene ID: 836). The sequence at its position 325–331, UACCUCA, was cloned into the site downstream of the synthetic Renilla luciferase gene. This sequence was mutated to ATGGAGT in the mutant group. HEK293 cells were seeded into 96-well plates, and 100 μl of transfection solution containing 0.5 μl Lipofectamine 2000 (Invitrogen, USA) and 25 ng reporter plasmids with 50 nM let-7b mimic or 50 nM mimic control, respectively, were added. The cells were washed and harvested 24 h after transfection. Luciferase activities were measured using the Dual-luciferase Reporter Assay Kit (Promega, USA) and Centro XS (Berthold technologies, Germany). The relative luciferase activity was normalized against the firefly luciferase values. The experiment was repeated in triplicate.

### Bioinformatics analysis

The sequences of rat H19 were retrieved from genebank (http://www.ncbi.nlm.nih.gov/gene) using its corresponding accession numbers of NR_027324.1. The sequences of rat mature miRNAs were retrieved from miRBase (http://www.mirbase.org/). The binding sites of miRNAs to H19 were predicted using the web-based program RNAhybrid^51^.

### Statistical analysis

All statistical analyses were performed using the GraphPad Prism 5 statistical software. Data were compared by Student’s *t*-test (two groups) or by one-way analysis of variance followed by appropriate multiple comparisons test (more than two groups). Data in all figures are expressed as mean ± standard error of the mean (s.e.m.).

## Electronic supplementary material

Supplemental Material 1 The differentially expressed lncRNAs

Supplemental Material 2 The differentially expressed mRNAs

Supplementary Figures
