# Supplementary material for: Long non-coding RNA H19 contributes to apoptosis of hippocampal neurons by inhibiting let-7b in a rat model of temporal lobe epilepsy
Source: Cell Death Dis. 2018 May 23;9(6):617. doi: 10.1038/s41419-018-0496-y (PMC5966382; doi:10.1038/s41419-018-0496-y)
Supplement: Supplementary file 1 — Supplemental Material 1 The differentially expressed lncRNAs [file 41419_2018_496_MOESM1_ESM.pdf]

Gene Symbol	Accession Number	Fold Change	p-value	Rank
H19	NR_027324	8.100401	0.000223	1
	ENSRNOT00000053541	-3.160898	0.00023	2
	Zfas1	2.862421	0.000315	3
Epha8	X59290	-2.766758	0.000576	4
Mir2964	NR_037382	-2.74637	0.000278	5
	BC086388	-2.685125	0.000237	6
Mir3546	NR_037320	-2.68479	0.000365	7
Mir434	NR_032278	2.592695	0.000523	8
	ENSRNOT00000062371	-2.574911	0.000628	9
	AF090348	-2.569908	0.000948	10
Mettl11b	FQ213916	-2.536278	0.000244	11
	ENSRNOT00000062455	2.481387	0.000661	12
	DQ486884	2.464361	0.000347	13
Moxd1	BC091331	-2.424328	0.000271	14
	ENSRNOT00000070294	-2.402805	0.000706	15
	ENSRNOT00000068981	2.352536	0.000737	16
	ENSRNOT00000053348	-2.280139	0.000713	17
Fndc5	AF529211	-2.24065	0.000549	18
	ENSRNOT00000062850	2.110732	0.000406	19
	ENSRNOT00000062780	2.087039	0.000555	20
Rnf149	BC086427	2.066526	0.000257	21
	ENSRNOT00000053394	2.048175	0.000592	22
Esyt3	FQ211602	-2.046701	0.000727	23
	ENSRNOT00000069607	-2.041587	0.000673	24
	ENSRNOT00000053524	1.933587	0.000372	25
	ENSRNOT00000062528	-1.9151	0.000491	26
	ENSRNOT00000053353	-1.831624	0.000792	27
LOC363181	BC082066	-1.799983	0.00076	28
LOC501396	BC079387	-1.785722	0.000299	29
	ENSRNOT00000063034	-1.780942	0.000621	30
	ENSRNOT00000053873	1.775825	0.000307	31
	ENSRNOT00000052451	1.764493	0.00075	32
	ENSRNOT00000052964	1.759047	0.00042	33
	ENSRNOT00000069864	-1.756739	0.000654	34
Coro2b	BC089991	-1.751412	0.000647	35
Wwc1	FQ212171	-1.749975	0.00034	36
Epm2a	AF347030	-1.749175	0.000292	37
	ENSRNOT00000052727	1.73975	6.00E-04	38
	ENSRNOT00000062418	-1.735234	0.000636	39
Gas5	NR_002704	1.718184	0.00025	40
	ENSRNOT00000069462	-1.711695	0.000453	41
C1qb	FQ234810	1.681008	0.000542	42
Tmem59l	FQ212329	-1.675845	0.000285	43
	ENSRNOT00000070770	1.675363	0.000614	44
	ENSRNOT00000052686	1.672341	0.000841	45
	ENSRNOT00000053816	1.670035	0.00087	46
	ENSRNOT00000053816	1.670035	0.00087	47
	FQ229993	1.663223	0.000434	48
	FQ229993	1.663223	0.000434	49
	FQ229993	1.663223	0.000434	50
	ENSRNOT00000070428	1.650469	0.000485	51
	ENSRNOT00000063600	-1.646631	0.000906	52
Mir344b-1	NR_037392	1.63764	0.000744	53

	ENSRNOT00000005120	-1.635974	0.000467	54
Tmcc2	FQ211467	-1.635465	0.000477	55
Hint3	FQ215151	-1.632514	0.000498	56
	ENSRNOT00000069075	1.606238	0.000607	57
Nop56	FQ224806	1.594296	0.00072	58
	ENSRNOT00000062543	1.582887	0.000683	59
	ENSRNOT00000062543	1.582887	0.000683	60
	ENSRNOT00000062416	1.580913	0.000326	61
	ENSRNOT00000069439	-1.57231	0.000832	62
Rps27l	FQ209888	1.56442	0.000413	63
Gars	BC088347	1.551162	0.00046	64
RGD1559747	NR_027235	-1.543451	0.000802	65
	J01884	1.507819	0.000937	66
	FQ213674	-1.500986	0.000392	67
	ENSRNOT00000069935	1.464598	0.000264	68
Nol9	BC098821	1.458434	0.000379	69
Mir22	NR_031824	1.457225	0.000699	70
Gars	BC088347	1.43997	0.000385	71
LOC10036315[illegible]	XR_146498	-1.423301	0.000333	72
	ENSRNOT00000053411	-1.413346	0.000896	73
	FQ232177	1.409871	0.00086	74
Rpl7l1	FQ214078	1.39735	0.000358	75
	FQ210617	-1.387009	0.000886	76
Utp6	FQ210246	1.386554	0.000399	77
Pvrl1	AF091111	-1.373363	0.000569	78
Siat7E	AJ646872	-1.366275	0.000809	79
Zfand1	FQ229099	-1.357277	0.000583	80
Mir219-2	NR_031933	-1.355967	0.000562	81
	ENSRNOT00000053479	1.324587	0.000923	82
Ndufc1	FQ217680	-1.319667	0.00078	83
	ENSRNOT00000047494	1.316686	0.000769	84
	ENSRNOT00000053854	-3.23157	0.001084	85
	ENSRNOT00000053969	-2.152363	0.001027	86
	BC087706	-2.037154	0.001129	87
	ENSRNOT00000053992	1.83488	0.001248	88
	ENSRNOT00000062974	-1.81737	0.001286	89
LOC303140	DQ119106	1.75169	0.001046	90
Mir27a	NR_031833	1.730268	0.001166	91
	ENSRNOT00000040329	1.688447	0.000955	92
	ENSRNOT00000063760	1.651086	0.000966	93
	BC091362	-1.61226	0.00134	94
	ENSRNOT00000063242	-1.582746	0.001097	95
	ENSRNOT00000052533	1.528018	0.001309	96
	ENSRNOT00000052533	1.528018	0.001309	97
Rn45s	NR_046239	1.492002	0.001147	98
	ENSRNOT00000054038	1.461059	0.001011	99
Mir219-1	NR_031932	1.453595	0.000985	100
	AJ240056	1.447056	0.001061	101
	ENSRNOT00000031588	-1.401179	0.001053	102
Mapk8ip2	BC105884	-1.339863	0.001104	103
Arf4	FQ234248	-1.332215	0.001034	104
	ENSRNOT00000069567	-1.283559	0.000978	105
	FQ224565	-1.248269	0.001112	106
Mir185	NR_031903	-1.212933	0.001229	107

	ENSRNOT00000062460	-2.27024	0.001433	108
	ENSRNOT00000062912	-2.197459	0.001386	109
	ENSRNOT00000053806	-2.063504	0.00144	110
	ENSRNOT00000068906	1.790244	0.001463	111
LOC367381	BC079433	-1.692196	0.001629	112
	ENSRNOT00000070144	-1.649517	0.001755	113
	ENSRNOT00000054025	-1.618942	0.001605	114
Mir21	NR_031823	1.606789	0.001363	115
	ENSRNOT00000062261	1.50509	0.001662	116
	ENSRNOT00000062791	1.501743	0.001716	117
	ENSRNOT00000052073	1.452751	0.001521	118
LOC497848	XR_006411	-1.394269	0.00151	119
	FQ233740	-1.369608	0.001689	120
	ENSRNOT00000016107	1.368878	0.001729	121
	ENSRNOT00000052835	1.359129	0.001412	122
LOC10036256З	XR_086023	-1.34443	0.001581	123
Rarb	AJ002942	-1.323453	0.001451	124
Nup188	BC086451	1.294426	0.001369	125
	BC089955	-1.244431	0.001722	126
Eri3	BC167080	-1.202449	0.001426	127
Itga2	AB067445	3.033315	0.002104	128
	ENSRNOT00000053261	-2.177177	0.002154	129
Mir3554	NR_037332	2.155589	0.001787	130
	ENSRNOT00000053991	-2.052523	0.001979	131
	NC_001665	-1.941543	0.001908	132
	ENSRNOT00000062407	-1.88897	0.001872	133
	ENSRNOT00000062667	1.796761	0.002066	134
	ENSRNOT00000063156	1.780029	0.00246	135
	ENSRNOT00000054218	1.762175	0.002055	136
	ENSRNOT00000063742	-1.652251	0.002497	137
	Zfas1	1.601084	0.00249	138
	ENSRNOT00000052762	1.586229	0.002005	139
	ENSRNOT00000053922	-1.558339	0.001926	140
	ENSRNOT00000063435	1.529717	0.002441	141
	ENSRNOT00000052996	1.522209	0.001762	142
	NC_001665	-1.500158	0.002344	143
	ENSRNOT00000070509	-1.436516	0.002466	144
Kcnq4	AF249748	-1.414048	0.001954	145
	FQ231321	-1.411069	0.002097	146
	ENSRNOT00000053390	1.338463	0.002083	147
	FQ231069	-1.32688	0.001967	148
	FQ234399	1.302451	0.001914	149
Dnlz	NR_024073	1.296572	0.001771	150
	ENSRNOT00000070488	1.288126	0.001937	151
	FQ226363	1.245076	0.002197	152
	ENSRNOT00000062799	-2.694078	0.002912	153
	ENSRNOT00000069568	-2.283756	0.003694	154
Mir132	NR_031878	2.162351	0.003289	155
	ENSRNOT00000054537	2.077352	0.004084	156
	ENSRNOT00000025224	-2.017595	0.003818	157
Mir3074	NR_037331	1.938173	0.002594	158
	ENSRNOT00000063727	-1.900456	0.002855	159
Mir410	NR_032274	1.849014	0.00344	160
Sfrp1	AF167308	1.834293	0.002827	161

	ENSRNOT00000063262	1.770016	0.003458	162
	ENSRNOT00000053548	1.738827	0.003243	163
	ENSRNOT00000062557	-1.698309	0.003557	164
	ENSRNOT00000054437	-1.694747	0.003964	165
	ENSRNOT00000053523	-1.673748	0.002737	166
	ENSRNOT00000062493	-1.636298	0.002675	167
	ENSRNOT00000052511	-1.606341	0.004238	168
	ENSRNOT00000062467	1.596271	0.003944	169
	ENSRNOT00000069429	-1.578917	0.004279	170
	Bsr	-1.576143	0.004161	171
	ENSRNOT00000069085	-1.574901	0.002806	172
	ENSRNOT00000063323	-1.574198	0.004134	173
Gpr176	D38450	1.553051	0.00266	174
	ENSRNOT00000053050	-1.550862	0.003544	175
	ENSRNOT00000062343	-1.500115	0.003014	176
Lrrc25	FQ228377	1.483043	0.004312	177
	ENSRNOT00000052494	1.476082	0.002838	178
	ENSRNOT00000069514	1.445077	0.003517	179
	ENSRNOT00000052797	1.4334	0.002929	180
	ENSRNOT00000052774	-1.427124	0.002973	181
Zhx2	AB081946	-1.417293	0.003364	182
Aoc2-ps1	NR_033180	1.413452	0.003222	183
Mir674	NR_032290	1.395425	0.003953	184
LOC100912852	XM_003754639	-1.383507	0.002957	185
	ENSRNOT00000068966	1.383436	0.004347	186
	ENSRNOT00000062748	-1.381174	0.003501	187
	ENSRNOT00000062711	1.37269	0.003684	188
	ENSRNOT00000062285	1.369419	0.003757	189
	ENSRNOT00000069961	1.35904	0.004128	190
Med23	BC090022	1.351639	0.003041	191
	ENSRNOT00000070301	-1.351059	0.003129	192
Fam195b	BC098826	1.335231	0.004221	193
	ENSRNOT00000052877	1.33464	0.003157	194
Mir3561	NR_037343	1.328046	0.002785	195
	ENSRNOT00000070231	-1.303001	0.003336	196
	ENSRNOT00000070231	-1.303001	0.003336	197
	ENSRNOT00000053364	1.299587	0.004406	198
	ENSRNOT00000062839	-1.290711	0.003229	199
Oip5	BC167069	1.255681	0.002601	200
	ENSRNOT00000069963	-1.254608	0.004095	201
	ENSRNOT00000069963	-1.254608	0.004095	202
LOC100360682	FQ211122	1.244158	0.002555	203
Pisd	FQ234220	1.243985	0.002862	204
Abhd11	BC088239	1.238193	0.003916	205
Abhd11	BC088239	1.236359	0.003411	206
	ENSRNOT00000053902	1.232528	0.003853	207
Zfp496	FQ223523	1.229104	0.00328	208
	ENSRNOT00000062573	1.222133	0.004291	209
Smg5	BC169031	1.20548	0.003579	210
U2af2	BC089996	1.201503	0.003904	211
	ENSRNOT00000054592	2.439393	0.007044	212
	ENSRNOT00000062320	-2.163952	0.007518	213
Mir568	NR_032745	-2.028959	0.004626	214
	ENSRNOT00000062506	-1.935753	0.00602	215

	ENSRNOT00000068862	1.830804	0.008187	216
Mir374	NR_032135	-1.808736	0.00533	217
	ENSRNOT00000069984	1.774808	0.005744	218
	ENSRNOT00000070411	-1.691497	0.007267	219
	ENSRNOT00000063194	1.677322	0.007037	220
	ENSRNOT00000062658	-1.672134	0.005988	221
	ENSRNOT00000062363	1.663396	0.005556	222
	ENSRNOT00000053547	1.630636	0.005574	223
	ENSRNOT00000054149	1.613311	0.005105	224
	ENSRNOT00000053812	-1.585888	0.005796	225
	ENSRNOT00000068911	1.579008	0.005766	226
	ENSRNOT00000062236	-1.562044	0.004994	227
	ENSRNOT00000062324	1.559443	0.006076	228
	ENSRNOT00000070268	1.555186	0.008168	229
	ENSRNOT00000062422	1.553943	0.006491	230
	Bsr	-1.538297	0.006879	231
	GAS5	1.533574	0.005893	232
	ENSRNOT00000062411	-1.525741	0.004913	233
	ENSRNOT00000069132	-1.522952	0.005196	234
Mir23b	NR_031826	1.508106	0.008259	235
	ENSRNOT00000063361	-1.499036	0.006612	236
	ENSRNOT00000054089	-1.496477	0.005203	237
	ENSRNOT00000053391	-1.493822	0.007298	238
	GAS5	1.468107	0.006622	239
	ENSRNOT00000068879	-1.458505	0.00554	240
	ENSRNOT00000054151	1.456346	0.00714	241
	ENSRNOT00000063015	-1.426461	0.005759	242
	ENSRNOT00000053942	-1.422378	0.005059	243
	ENSRNOT00000054352	1.39415	0.005596	244
	ENSRNOT00000053327	-1.392364	0.004579	245
	ENSRNOT00000053327	-1.392364	0.004579	246
	ENSRNOT00000070231	-1.39024	0.004987	247
	ENSRNOT00000062861	1.378607	0.006095	248
	GAS5	1.375971	0.004878	249
	ENSRNOT00000052967	-1.374019	0.005414	250
	ENSRNOT00000052967	-1.374019	0.005414	251
	ENSRNOT00000052967	-1.373977	0.004771	252
	ENSRNOT00000052967	-1.373977	0.004771	253
	ENSRNOT00000052967	-1.373977	0.004771	254
	ENSRNOT00000052967	-1.373977	0.004771	255
	ENSRNOT00000052967	-1.373977	0.004771	256
	ENSRNOT00000052967	-1.373977	0.004771	257
	ENSRNOT00000052967	-1.373977	0.004771	258
	ENSRNOT00000052967	-1.373977	0.004771	259
	ENSRNOT00000052967	-1.373977	0.004771	260
	ENSRNOT00000052967	-1.373977	0.004771	261
Deptor	XM_001066889	-1.371057	0.006244	262
	ENSRNOT00000054610	-1.368741	0.004503	263
	ENSRNOT00000070264	1.36668	0.005467	264
	ENSRNOT00000063652	-1.366646	0.006181	265
	ENSRNOT00000052868	-1.365423	0.005433	266
	ENSRNOT00000068796	-1.364759	0.005458	267
	ENSRNOT00000063002	-1.364544	0.005813	268
	ENSRNOT00000069925	-1.354078	0.005112	269

	ENSRNOT00000069137	1.353386	0.005706	270
	ENSRNOT00000070256	1.352529	0.007978	271
	ENSRNOT00000053175	-1.350128	0.0059	272
	FQ225434	-1.347075	0.004552	273
	GAS5	1.340047	0.007723	274
	ENSRNOT00000069295	1.336587	0.005785	275
	Zfas1	1.330435	0.005917	276
	ENSRNOT00000062375	1.328515	0.006501	277
	ENSRNOT00000062375	1.328515	0.006501	278
Rpl10	FQ220555	1.327357	0.005066	279
	ENSRNOT00000069800	-1.326122	0.005122	280
Mir499	NR_032141	1.325582	0.007388	281
	ENSRNOT00000053425	1.32254	0.006067	282
	ENSRNOT00000053497	1.320336	0.004654	283
	ENSRNOT00000069162	1.309561	0.00496	284
	ENSRNOT00000069164	-1.301584	0.004936	285
	ENSRNOT00000054060	1.298757	0.006188	286
	ENSRNOT00000053402	1.292899	0.005257	287
	ENSRNOT00000062773	-1.292039	0.005937	288
	ENSRNOT00000069869	1.281621	0.007121	289
	ENSRNOT00000053496	1.277908	0.005399	290
	ENSRNOT00000053218	1.276488	0.007689	291
	ENSRNOT00000053195	1.276229	0.004943	292
	ENSRNOT00000062390	1.275093	0.005589	293
	ENSRNOT00000069068	-1.274249	0.005073	294
	ENSRNOT00000062555	1.273282	0.007082	295
	ENSRNOT00000054251	1.273189	0.007418	296
	ENSRNOT00000054632	1.269583	0.008084	297
Mir375	NR_032271	1.265837	0.005225	298
	FQ216506	1.264918	0.00505	299
	ENSRNOT00000070073	1.2598	0.004637	300
RGD1309995	FQ222739	1.255113	0.007061	301
	FQ227485	-1.25195	0.007604	302
Ipo11	BC092638	-1.245271	0.004603	303
	ENSRNOT00000068893	1.242074	0.004896	304
Mir3553	NR_037330	1.241182	0.005169	305
RGD1303117	NR_037147	1.233656	0.005238	306
	ENSRNOT00000069265	-1.229558	0.006729	307
	ENSRNOT00000052790	1.228047	0.007573	308
	ENSRNOT00000062305	1.22606	0.007128	309
	ENSRNOT00000023316	1.221093	0.004644	310
Ankrd10	BC098779	1.217719	0.00529	311
	ENSRNOT00000068836	1.214142	0.007639	312
Tmem80	NR_045202	-1.205103	0.006333	313
