# Supplementary material for: Long non-coding RNA H19 contributes to apoptosis of hippocampal neurons by inhibiting let-7b in a rat model of temporal lobe epilepsy
Source: Cell Death Dis. 2018 May 23;9(6):617. doi: 10.1038/s41419-018-0496-y (PMC5966382; doi:10.1038/s41419-018-0496-y)
Supplement: Supplementary file 2 — Supplemental Material 2 The differentially expressed mRNAs [file 41419_2018_496_MOESM2_ESM.pdf]

Gene Symbol	Accession Number	Fold Change	p-value	Rank
Ccl2	NM_031530	82.445463	4.80E-05	1
Serpine1	ENSRNOT00000001916	61.620505	4.50E-05	2
Trh	ENSRNOT00000015944	45.971225	6.10E-05	3
Serpinb2	NM_021696	45.835052	0.00018	4
Timp1	NM_053819	31.480955	4.40E-05	5
Hspb1	NM_031970	27.128222	5.80E-05	6
Ccl7	NM_001007612	24.821817	0.000166	7
Sprr1a	ENSRNOT00000068099	20.530092	0.000103	8
Serpina3n	ENSRNOT00000014073	18.661864	0.000221	9
Itga5	NM_001108118	16.962659	8.20E-05	10
Lgals3	NM_031832	16.254612	5.70E-05	11
Lox	NM_017061	15.633711	0.000282	12
Spp1	NM_012881	14.021988	0.000175	13
Tfpi2	ENSRNOT00000013989	13.372206	0.000139	14
Tuba1c	NM_001011995	12.989019	6.50E-05	15
Cd14	ENSRNOT00000023977	12.92542	8.70E-05	16
Inhba	NM_017128	12.898756	9.20E-05	17
Osmr	ENSRNOT00000040847	11.939514	9.40E-05	18
Msr1	NM_001191939	11.917834	5.90E-05	19
Tubb6	NM_001025675	11.239949	7.50E-05	20
Tnfrsf12a	ENSRNOT00000004842	10.967068	4.90E-05	21
Hbegf	ENSRNOT00000025157	10.872804	5.50E-05	22
Emp1	NM_012843	10.588872	5.00E-05	23
Flnc	NM_001191862	10.551716	4.60E-05	24
Fosl1	ENSRNOT00000027891	9.770313	9.10E-05	25
Gprc5a	NM_001079890	9.203857	0.000179	26
Socs3	NM_053565	8.958824	6.60E-05	27
Cyr61	ENSRNOT00000019501	8.784129	0.000324	28
Gadd45g	ENSRNOT00000018252	8.563947	0.000155	29
Fgl2	NM_053455	8.554343	7.60E-05	30
Csf2rb	ENSRNOT00000000203	8.361756	6.90E-05	31
Gem	NM_001106637	7.909201	1.00E-04	32
Fcgr2b	NM_175756	7.889241	9.70E-05	33
Glra1	NM_013133	7.706654	0.000168	34
Tmbim1	NM_001007713	7.656939	4.20E-05	35
P4ha3	ENSRNOT00000046943	7.645386	0.000743	36
Gjb6	NM_053388	-7.603804	0.000217	37
S100a10	NM_031114	7.572394	5.30E-05	38
Fos	NM_022197	7.532718	0.001077	39
Msn	ENSRNOT00000040148	7.482452	6.70E-05	40
Plp2	NM_207601	7.480787	7.80E-05	41
Clec7a	ENSRNOT00000052373	7.437399	0.000121	42
Il1rn	NM_022194	7.399558	0.001874	43
Plin2	ENSRNOT00000009749	7.34418	5.20E-05	44
Htr5b	NM_024395	-7.329792	0.000214	45
Reg3g	NM_173097	7.216983	0.00201	46
Glipr2	ENSRNOT00000019916	7.121086	7.00E-05	47
S1pr3	XM_225216	6.939498	0.000391	48
Clic1	NM_001002807	6.8464	9.60E-05	49
Adamts1	NM_024400	6.647144	6.20E-05	50
Fstl3	ENSRNOT00000012578	6.638523	0.000153	51
LOC299277	XM_001074308	6.636007	0.000818	52
Cldn10	NM_001106058	-6.500473	0.000135	53

Gldn	NM_181382	6.367905	0.000649	54
Cd44	ENSRNOT00000009000	6.355755	7.10E-05	55
Zfp36	NM_133290	6.340447	9.00E-05	56
LOC100363184	XM_003752471	6.286944	0.002826	57
	ENSRNOT00000013475	6.215922	0.000109	58
Pdpn	ENSRNOT00000020316	5.888326	8.00E-05	59
Hmox1	NM_012580	5.870463	0.000557	60
Kctd4	NM_001109650	-5.823536	0.000403	61
Il11	NM_133519	5.787922	0.000212	62
Mmp9	ENSRNOT00000023965	5.784478	0.001492	63
PVR	ENSRNOT00000064305	5.774526	0.00013	64
Sdc1	NM_013026	5.765137	0.000368	65
Trib1	NM_023985	5.757075	0.000126	66
Lif	NM_022196	5.592575	0.000189	67
Tagln2	NM_001013127	5.567383	6.30E-05	68
Fam129b	NM_001109885	5.506872	7.30E-05	69
Atf3	NM_012912	5.492544	0.0015	70
Hspb8	NM_053612	5.457607	8.30E-05	71
LOC10036088C	NM_001256509	5.399664	0.000659	72
Gpnmb	ENSRNOT00000011945	5.39604	0.000134	73
Fbln2	ENSRNOT00000009696	5.38591	0.000171	74
Rassf4	NM_001024275	5.355688	0.000259	75
Cbln1	NM_001109127	5.298564	0.001199	76
LOC10036229€	ENSRNOT00000055513	5.260901	0.000457	77
	ENSRNOT00000055802	5.162475	0.00079	78
Lcn2	ENSRNOT00000018776	5.089372	0.001804	79
Lsp1	NM_001025420	5.086443	0.00015	80
Nov	NM_030868	-5.07386	0.000451	81
Rai14	NM_001011947	5.027223	0.000394	82
Anxa2	ENSRNOT00000038677	5.019168	0.000414	83
S100a11	NM_001004095	4.986544	0.000518	84
Hmgcs2	ENSRNOT00000026121	-4.979926	0.001628	85
Penk	NM_017139	4.943723	9.50E-05	86
Cxcl16	NM_001017478	4.926148	0.000522	87
Pmepa1	ENSRNOT00000008261	4.888024	5.40E-05	88
Tnfsf18	ENSRNOT00000039221	4.846564	0.001992	89
Fmo9p	NM_001109466	4.800566	0.000628	90
Clec12a	NM_001134716	4.764069	0.000125	91
Nptx2	NM_001034199	4.761281	0.00016	92
Gadd45a	ENSRNOT00000007698	4.746356	0.00026	93
Il13ra1	NM_145789	4.744748	0.00012	94
Il13ra1	NM_145789	4.706002	0.000114	95
Igfbp3	NM_012588	4.701869	0.000327	96
Vgf	NM_030997	4.698169	0.000353	97
Slc5a3	ENSRNOT00000002749	4.680987	0.000281	98
Sox11	ENSRNOT00000045963	4.675587	0.000252	99
Slco1c1	NM_053441	-4.655541	0.000208	100
A3galt2	NM_138524	4.640719	0.000273	101
Gadd45b	NM_001008321	4.631224	0.000206	102
Htr1a	ENSRNOT00000013618	-4.561002	0.00055	103
Agbl1	ENSRNOT00000031334	4.536958	0.001188	104
Tll1	NM_001106081	4.5256	0.000907	105
RGD1562846	BC167027	4.494129	0.000376	106
Tnfsf10	NM_145681	-4.483982	0.000204	107

	ENSRNOT00000064950	4.477063	0.00248	108
Il1r2	ENSRNOT00000019415	4.471179	0.000732	109
Gal	NM_033237	4.455386	0.001368	110
	ENSRNOT00000039392	4.43454	0.00042	111
Procr	NM_001025733	4.426559	0.000428	112
LOC100363941	ENSRNOT00000008559	4.402347	0.000512	113
Fam110c	NM_001025051	4.340676	0.000939	114
Itgam	ENSRNOT00000026748	4.326304	0.000105	115
Elf4	NM_001191735	4.283538	0.000285	116
Il31ra	NM_001257278	4.261997	0.000314	117
Csrnp1	NM_001108786	4.251078	0.000284	118
Car14	NM_001109655	-4.178411	0.000593	119
Acss1	NM_001106524	-4.135348	0.000246	120
RGD1563982	BC091204	4.088749	0.000196	121
Gabre	ENSRNOT00000022431	4.062882	0.00108	122
Pappa	ENSRNOT00000040898	4.046446	0.001793	123
Tmem100	NM_001017479	4.019913	0.000132	124
Tril	NM_001034010	-4.005493	0.000159	125
Cml3	ENSRNOT00000021103	-3.993018	0.000989	126
Gjc3	XM_221997	-3.97931	0.000341	127
Ctsc	NM_017097	3.967389	0.000219	128
Adhfe1	NM_001025423	-3.966802	0.000151	129
Ret	NM_012643	3.958938	0.000511	130
Prodh	NM_001135778	-3.947717	0.000172	131
Timp4	NM_001109393	-3.945153	0.000116	132
Cbs	NM_012522	-3.940312	0.000336	133
Ermn	ENSRNOT00000034449	-3.933063	0.001691	134
Clcf1	NM_207615	3.929498	0.000235	135
Adamts9	NM_001107877	3.921133	0.000145	136
P2ry13	NM_001002853	-3.914278	0.000489	137
Hes5	ENSRNOT00000018769	-3.906493	0.000474	138
Serinc2	NM_001031656	3.858061	0.000216	139
Ogn	NM_001106103	-3.852047	0.00206	140
P2ry12	NM_022800	-3.844633	0.000553	141
Dusp2	NM_001012089	3.841828	0.001878	142
Plaur	NM_134352	3.837987	0.000311	143
Trem1	NM_001106885	3.834455	0.000825	144
Slc22a8	NM_031332	-3.825674	0.000107	145
Ier3	NM_212505	3.815732	0.00029	146
Crhbp	NM_139183	3.810307	0.000564	147
Fam167a	NM_001109102	3.798568	0.000231	148
Agpat9	NM_001025670	3.791653	0.000302	149
Acvr1c	ENSRNOT00000059280	3.783316	0.001206	150
Hapln1	NM_019189	-3.778524	0.000349	151
Cd63	NM_017125	3.777181	8.80E-05	152
Slco2a1	NM_022667	3.77146	0.001143	153
Fam163a	NM_001109072	-3.77034	0.000322	154
Slc18a3	NM_031663	3.76091	0.000571	155
Dbp	ENSRNOT00000028546	-3.752483	0.000187	156
Trim47	NM_001109585	3.738426	8.40E-05	157
Nupr1	NM_053611	3.724616	0.000351	158
Ch25h	NM_001025415	3.723896	0.000326	159
Srxn1	NM_001047858	3.708889	0.000226	160
Lix1	NM_001106214	-3.70357	0.000275	161

Sik1	ENSRNOT00000001579	3.682622	0.000385	162
Lrrc15	NM_145083	3.676188	0.000402	163
S100a4	NM_012618	3.662476	0.000142	164
Stat3	NM_012747	3.647233	7.90E-05	165
Neurod6	NM_001109237	-3.62993	0.001243	166
Il6	NM_012589	3.62883	0.002675	167
Fam38a	NM_001077200	3.623143	0.000141	168
Emp3	NM_030847	3.608364	0.000387	169
	ENSRNOT00000060689	3.589694	0.000271	170
Gstt3	NM_001137643	-3.572631	0.000673	171
LOC500300	NM_001024334	3.571962	0.000755	172
Cdkn1a	NM_080782	3.561416	0.00133	173
Map3k6	NM_001107909	3.539882	0.000117	174
Lyz2	ENSRNOT00000007747	3.529984	0.000258	175
Olr522	NM_001000562	3.526889	0.001769	176
Sidt1	NM_001100653	-3.524616	0.000137	177
Gsta3	NM_031509	-3.519896	0.000666	178
	NM_207601	3.518469	0.000112	179
Akr1b8	NM_173136	3.516343	0.002326	180
Itm2a	NM_001025712	-3.480477	0.000418	181
Flna	NM_001134599	3.462938	0.000269	182
Nfil3	NM_053727	3.458696	0.000289	183
Kcnk10	NM_023096	3.455804	0.000684	184
Agpat2	NM_001107821	3.447938	0.000338	185
Ncan	NM_031653	3.447665	9.90E-05	186
Tinagl1	ENSRNOT00000018464	3.442687	0.001232	187
Gpr34	ENSRNOT00000060988	-3.442644	0.000974	188
Ptgs2	ENSRNOT00000003567	3.434539	0.000752	189
Mdfic	NM_001105668	3.423401	0.000143	190
Ninj2	ENSRNOT00000013721	-3.399243	0.001119	191
Nhlh1	NM_001105970	-3.398363	0.000432	192
	GENSCAN00000024327	3.398164	0.000164	193
Tnfrsf1a	ENSRNOT00000048529	3.393443	0.000104	194
	ENSRNOT00000065210	-3.372746	0.000837	195
Prss23	NM_001007691	3.371612	0.00063	196
Olfml1	NM_001013192	-3.369777	0.000605	197
Cxcl12	ENSRNOT00000066670	-3.364907	0.00036	198
Mal	ENSRNOT00000020870	-3.361233	0.002679	199
Mthfd2	NM_001109398	3.350167	0.000188	200
Sbno2	ENSRNOT00000068197	3.349734	0.000133	201
Olr1	NM_133306	3.349482	0.00166	202
Rbp1	NM_012733	3.345	0.000795	203
Grm3	NM_001105712	-3.314157	0.000317	204
Myc	NM_012603	3.29132	0.000201	205
Evi2a	NM_001044287	-3.285133	0.000757	206
Aifm3	NM_001013977	-3.280562	0.000439	207
Spry1	NM_001106427	3.280263	0.000369	208
Map2k6	ENSRNOT00000006217	-3.27871	0.000366	209
Pim1	ENSRNOT00000000637	3.277336	0.000491	210
Fmo1	NM_012792	-3.273191	0.000585	211
Pdlim1	NM_017365	3.265756	0.00052	212
Psd2	NM_001107395	-3.252231	0.000158	213
Tac1	NM_012666	3.244758	0.001885	214
Sox7	NM_001106045	3.241299	0.000631	215

Tax1bp3	NM_001025419	3.237746	0.000101	216
Slfn13	NM_001013970	3.236614	0.000124	217
Pde6b	NM_001106024	3.212842	0.002611	218
Agt	NM_134432	-3.206174	8.60E-05	219
Pamr1	NM_001107755	-3.204341	0.000478	220
Nes	NM_012987	3.202858	0.000669	221
Slc1a5	NM_175758	3.202445	0.000191	222
Cyp4f4	NM_173123	-3.181274	0.000229	223
Col6a3	ENSRNOT00000026707	3.174855	0.001349	224
Bag3	NM_001011936	3.170566	0.000469	225
Prom1	NM_021751	-3.162062	0.000233	226
Cyp7b1	NM_019138	-3.159079	0.001021	227
Opalin	NM_001017386	-3.156752	0.002306	228
RGD1309821	ENSRNOT00000033235	-3.152682	0.000111	229
Selenbp1	NM_080892	-3.150045	0.000243	230
Pla1a	ENSRNOT00000038400	3.142037	0.000242	231
Dusp5	ENSRNOT00000018889	3.136774	0.000964	232
LOC100360501	ENSRNOT00000022241	3.135907	7.40E-05	233
Sorcs3	NM_001106367	3.120074	0.001083	234
Ppap2b	NM_138905	-3.11989	0.000183	235
Adra1d	ENSRNOT00000028877	-3.118956	0.000292	236
	ENSRNOT00000020260	3.112764	0.000768	237
Tnip2	NM_001024771	3.107876	0.000209	238
Ano3	ENSRNOT00000006349	-3.086479	0.002337	239
Ccdc85a	NM_001191553	-3.084739	0.00109	240
Lix1	NM_001106214	-3.083965	0.000239	241
Ndst4	ENSRNOT00000012773	-3.072333	0.000667	242
Pdyn	NM_019374	3.063465	0.002341	243
Ifitm3	ENSRNOT00000020265	3.059763	0.000529	244
Tst	NM_012808	-3.050213	0.000265	245
Ptgds	NM_013015	-3.040599	0.001957	246
Has1	NM_172323	3.040264	0.000857	247
Igfbp5	ENSRNOT00000023530	-3.039964	0.00089	248
Xirp1	ENSRNOT00000024955	3.03699	0.001894	249
Slco1a2	NM_131906	-3.034253	0.000373	250
Baz1a	NM_001170568	3.033972	0.000176	251
Igsf9b	XM_001054496	3.031619	0.001847	252
Gamt	NM_001207007	-3.030183	0.000817	253
Abcd2	ENSRNOT00000021064	-3.029758	0.000681	254
Angpt2	NM_134454	3.026852	0.000223	255
Fn3k	ENSRNOT00000054915	-3.019645	0.000345	256
Atp13a4	NM_001191658	-3.003702	0.000147	257
Oxtr	NM_012871	3.002902	0.001301	258
Cd93	NM_053383	2.995526	0.001087	259
Scimp	XM_003752341	2.995254	0.0021	260
Hnmt	ENSRNOT00000007471	-2.98623	0.000193	261
Glipr1	NM_001011987	2.985354	0.000415	262
Tfec	ENSRNOT00000008156	2.980132	0.000305	263
Enpp2	ENSRNOT00000005561	-2.972054	0.002595	264
Cd68	NM_001031638	2.969607	0.00054	265
Kcng4	NM_001107435	-2.969011	0.000618	266
Dusp15	NM_001108598	-2.958565	0.000307	267
Angpt1	ENSRNOT00000007979	-2.957833	0.000177	268
Mustn1	NM_181368	2.953337	0.002546	269

Tnfsf13	NM_001009623	-2.945665	0.002103	270
Adam12	XM_002725723	2.927917	0.000535	271
Klf6	NM_031642	2.927796	0.000525	272
	ENSRNOT00000066977	-2.91266	0.000279	273
LOC689499	XM_001067525	-2.911084	0.000672	274
Tm4sf1	NM_001106434	2.90679	0.001621	275
Rgs1	NM_019336	2.906717	0.001752	276
	ENSRNOT00000039027	-2.90349	0.001208	277
Padi2	NM_017226	-2.896771	0.001317	278
Cmtm5	NM_001106034	-2.892476	0.001027	279
Gfap	NM_017009	2.892351	0.000507	280
LOC501110	NM_001024361	-2.886226	0.001435	281
Mthfd2	NM_001109398	2.886132	0.000162	282
	ENSRNOT00000019816	2.882248	0.000462	283
Ptx3	NM_001109536	2.879232	0.00073	284
Cd200r1	NM_023953	2.87822	0.002035	285
Ranbp3l	ENSRNOT00000032327	-2.87791	0.002366	286
Slc25a18	NM_001044280	-2.869126	0.000192	287
Slc15a2	NM_031672	-2.865335	0.000934	288
Gpr17	NM_001071777	-2.858477	0.000514	289
Kif18a	NM_001137642	2.840505	0.000583	290
LOC681325	ENSRNOT00000030692	2.839823	0.00071	291
Tgfbi	NM_053802	2.833891	0.001496	292
Chst3	ENSRNOT00000000697	2.824895	0.000426	293
Ehd2	NM_001024897	2.824791	0.000156	294
Aqp9	NM_022960	-2.806383	0.000437	295
Ms4a11	ENSRNOT00000033795	2.79835	0.000482	296
Akap5	ENSRNOT00000008416	-2.797673	0.001002	297
Aadat	NM_017193	-2.792592	0.00171	298
Npy	NM_012614	2.791944	0.000809	299
Ifitm1	NM_001106314	2.789065	0.000821	300
Plat	ENSRNOT00000025763	2.787386	0.00028	301
Shmt2	NM_001008322	2.78213	0.000146	302
Cpg1	NM_178104	2.781962	0.001079	303
Mt1a	ENSRNOT00000038212	2.7792	0.001161	304
Sh3bp2	NM_001100684	2.779191	7.00E-04	305
Ezr	NM_019357	2.778177	0.000427	306
3-Mar	NM_001007759	2.776076	0.000431	307
Tgm2	ENSRNOT00000018328	2.775118	0.000685	308
Prelp	NM_053385	-2.770636	0.000788	309
Ttpa	ENSRNOT00000009611	-2.765907	0.000202	310
Pnma3	NM_001106342	-2.757367	0.000237	311
Cml1	NM_021668	-2.752755	0.000615	312
Crisp1	ENSRNOT00000018163	2.748355	0.001975	313
Sphk1	NM_133386	2.745783	0.000294	314
Runx1	ENSRNOT00000002313	2.745337	0.001388	315
Vim	NM_031140	2.743152	0.000301	316
Clec4a3	ENSRNOT00000013308	2.742498	0.001352	317
Kcnip4	NM_181365	-2.735712	0.00132	318
Kcnt2	ENSRNOT00000064223	-2.729275	0.00066	319
Ano6	NM_001108108	2.729051	0.000277	320
Selplg	NM_001013230	-2.724593	0.000167	321
Capg	NM_001013086	2.723548	0.000227	322
Gja1	NM_012567	-2.722378	0.000411	323

Slc6a11	ENSRNOT00000008342	-2.719072	0.000352	324
Enpp3	NM_019370	2.716808	0.002648	325
	ENSRNOT00000037015	-2.706372	0.000251	326
Pthlh	NM_012636	2.704747	0.001346	327
Cxcl1	ENSRNOT00000003778	2.702452	0.001036	328
	ENSRNOT00000030613	-2.702152	0.00032	329
Slc38a3	ENSRNOT00000023623	-2.699366	0.000844	330
Hmga1	ENSRNOT00000000580	2.694537	0.000149	331
Upp1	ENSRNOT00000006765	2.692108	0.000218	332
Rgs2	ENSRNOT00000005156	2.678535	0.000515	333
Tpm4	ENSRNOT00000021073	2.674361	0.000331	334
Adcy1	NM_001107239	-2.670193	0.001813	335
RGD1359349	NM_001007738	2.668318	0.00232	336
Slc7a10	NM_053726	-2.663046	0.000364	337
Pou3f1	NM_138838	-2.660753	0.001283	338
Fpgs	NM_001146125	2.659732	0.000315	339
Sec14l2	NM_053801	-2.659235	0.001461	340
Has2	NM_013153	2.659162	0.00185	341
Cth	ENSRNOT00000067843	2.658057	0.000268	342
RGD1309537	NM_001135017	2.656251	0.00044	343
Kbtbd10	ENSRNOT00000009969	2.652614	0.002553	344
Pkp2	NM_001100499	-2.648707	0.002048	345
Pygm	NM_012638	-2.642154	0.00105	346
Casp3	ENSRNOT00000014095	2.634048	0.001187	347
Slc11a1	NM_001031658	2.632993	0.000785	348
	ENSRNOT00000057998	2.632119	0.000965	349
Cttnbp2nl	NM_001107712	2.63107	0.000138	350
Plau	NM_013085	2.629124	0.000556	351
	ENSRNOT00000043345	-2.62729	0.000113	352
Mlf1ip	NM_001025673	2.625894	0.00023	353
C3	NM_016994	2.625172	0.000545	354
Sult1d1	ENSRNOT00000060179	-2.624594	0.000729	355
Rdh5	ENSRNOT00000010217	2.624	0.001848	356
Nipal4	NM_001106995	-2.62393	0.000833	357
Prkab2	ENSRNOT00000063783	2.620247	0.000306	358
Prkcd	ENSRNOT00000025858	2.619694	0.002019	359
Arhgef25	NM_199395	-2.619194	0.002091	360
Fxyd5	ENSRNOT00000028597	2.618572	0.000398	361
Map2k3	NM_001100674	2.615376	0.000347	362
Lrp1b	NM_001107843	-2.611896	0.000296	363
Col4a1	NM_001135009	2.610971	0.000293	364
Stac2	NM_001108834	2.610929	0.001982	365
Rasgrp3	NM_001108009	-2.604877	0.00092	366
Slc4a4	NM_053424	-2.596008	0.000299	367
	ENSRNOT00000061713	-2.595363	0.00074	368
Hopx	NM_133621	2.592463	0.000423	369
Myl1	NM_001077656	2.588341	0.003032	370
Nkain4	NM_001106550	-2.586959	0.00017	371
Ccl6	ENSRNOT00000045867	2.575269	0.001202	372
Ripk3	ENSRNOT00000027759	2.570478	0.000494	373
	ENSRNOT00000005478	2.56944	0.001016	374
Gpx2	NM_183403	2.565578	0.002001	375
Rrad	ENSRNOT00000016259	2.55912	0.000873	376
Gpr3	ENSRNOT00000012710	2.557528	0.001677	377

Rab32	NM_001108902	2.551828	0.000393	378
Spsb1	NM_001107994	2.55141	0.000607	379
Agmo	NM_001135899	-2.544075	0.00039	380
	AF353304	-2.542884	0.000466	381
Lin7b	NM_021758	-2.540741	0.001594	382
Ccnf	NM_001100474	2.54057	0.000122	383
Scrg1	NM_033499	-2.539463	0.000731	384
Efemp1	NM_001012039	-2.539373	0.001505	385
Ppapdc1a	NM_001191631	2.538653	0.001755	386
Aox1	ENSRNOT00000068633	-2.531428	0.001277	387
Gsta4	NM_001106840	-2.530298	0.001919	388
Clcn2	NM_017137	-2.52614	0.000479	389
Itpkc	ENSRNOT00000018696	2.525188	0.000163	390
Pcdh19	NM_001169129	-2.52088	0.000503	391
Rgs16	NM_001077589	2.518209	0.001669	392
Tyrobp	NM_212525	2.517796	0.000261	393
Cyp2j3	ENSRNOT00000013087	-2.516986	0.000845	394
Il17ra	NM_001107883	2.515128	0.00041	395
Tlr1	NM_001172120	2.515014	0.000777	396
Grin2c	NM_012575	-2.510259	0.000477	397
Cyp3a9	NM_147206	-2.507386	0.000903	398
Pthlh	NM_012636	2.506887	0.001974	399
Cmbl	NM_001008770	-2.50654	0.000763	400
Calml4	NM_001127575	-2.506499	0.000255	401
Slc39a14	NM_001107275	2.501584	0.000548	402
Clic2	NM_001009651	2.500307	0.001393	403
Adck4	NM_001012065	2.499239	0.000247	404
Jph4	NM_001003711	-2.498048	0.000882	405
Rnase4	NM_020082	-2.497106	0.001644	406
Npm3	XM_577868	2.494059	0.000589	407
Wfdc6a	NM_001246283	2.493548	0.000792	408
LOC10036445[illegible]	ENSRNOT00000066265	2.48738	0.000332	409
Tpm3	NM_173111	2.487124	0.000272	410
Ifitm3	ENSRNOT00000020265	2.480955	0.000495	411
Arsj	NM_001047887	2.478903	0.001788	412
Neurod1	NM_019218	-2.477491	0.001192	413
Nfkbia	NM_001105720	2.475255	0.002079	414
Itih3	NM_017351	-2.475108	0.000901	415
Bdnf	NM_012513	2.472722	0.000715	416
Junb	NM_021836	2.472483	0.002577	417
Camkk2	ENSRNOT00000001774	-2.471809	0.000248	418
	GENSCAN00000013411	-2.470393	0.000355	419
Pnoc	NM_013007	2.467943	0.000383	420
Acss3	NM_001108091	-2.460241	0.001142	421
Prosapip1	ENSRNOT00000028835	-2.459678	0.000361	422
LOC683626	NM_001129880	2.457853	0.001058	423
LOC100364834	ENSRNOT00000058957	2.455535	0.002758	424
Cpne6	NM_001191113	-2.45135	0.000543	425
Itgb2	NM_001037780	2.449575	0.001074	426
Galntl4	ENSRNOT00000022984	-2.447252	0.000886	427
Anxa1	NM_012904	2.446775	0.001391	428
Lamc1	NM_053966	2.445043	0.000174	429
Itga11	NM_001108156	-2.444005	0.001737	430
Carhsp1	ENSRNOT00000003514	2.442239	0.000118	431

Lims1	ENSRNOT00000064288	2.441928	0.000154	432
Fhl3	NM_001107979	2.440248	0.000688	433
Ptn	ENSRNOT00000016088	-2.438921	0.00087	434
LOC100361272	XM_002725952	-2.437841	0.00239	435
Ankrd56	XM_223228	2.433595	0.001124	436
Sspn	NM_001109255	-2.428127	0.001196	437
Clmn	NM_001106755	-2.426393	0.001901	438
	ENSRNOT00000030206	-2.424603	0.00059	439
	ENSRNOT00000016839	2.42322	0.001721	440
Slc13a5	ENSRNOT00000020043	-2.421761	0.00051	441
Ccr1	NM_020542	2.419279	0.001458	442
Maff	NM_001130573	2.418282	0.001042	443
Gdf10	ENSRNOT00000027380	-2.418017	0.000869	444
Tgfb1	NM_021578	2.416939	0.000813	445
RGD1565350	ENSRNOT00000015338	2.416507	0.002014	446
Cars	NM_001106319	2.415456	0.000222	447
Ugt8	NM_019276	-2.413504	0.00204	448
Ptbp1	ENSRNOT00000041277	2.407695	0.000195	449
Maob	ENSRNOT00000044009	-2.405594	0.000389	450
Nedd9	NM_001011922	2.404279	0.000407	451
F2rl1	NM_053897	2.402883	0.000591	452
Per3	NM_023978	-2.401186	0.000377	453
Nfkb2	NM_001008349	2.401029	0.001183	454
Pawr	NM_033485	2.397647	0.001332	455
Casp4	ENSRNOT00000061973	2.397528	0.001413	456
Tmem2	NM_001107596	2.394594	0.002279	457
RGD1559896	NM_001109134	-2.394451	0.000637	458
Wwtr1	NM_001024869	2.388328	0.000205	459
Phlda1	NM_017180	2.386936	0.000877	460
Mchr1	ENSRNOT00000025564	2.386901	0.002222	461
Gpr37l1	NM_145784	-2.384132	0.000717	462
Gucy1a3	ENSRNOT00000017190	-2.380744	0.000957	463
LOC691418	XM_001068693	2.379863	0.000554	464
Myo5b	NM_017083	-2.378893	0.001378	465
Sypl2	NM_001108563	2.377891	0.000422	466
Fcgr1a	NM_001100836	2.374554	0.000184	467
Gstm6	NM_001109192	-2.370391	0.001575	468
Eif4ebp1	NM_053857	2.369049	0.000108	469
Il1r1	ENSRNOT00000019673	2.36844	0.000908	470
Tmem74	XM_576263	-2.367616	0.001475	471
LOC100360982	XM_002728907	2.365374	0.000803	472
Myo1e	NM_173101	2.361731	0.000185	473
Rtn4r	ENSRNOT00000051037	-2.359985	0.001038	474
Kit	ENSRNOT00000003050	-2.356067	0.002002	475
Clcn4	NM_022198	-2.354844	0.001046	476
Vtn	ENSRNOT00000039954	-2.35383	0.000339	477
Ddc	NM_012545	-2.353286	0.00061	478
Cd86	NM_020081	2.352142	0.001179	479
Nfam1	ENSRNOT00000037681	-2.351406	0.001292	480
Slc8a2	ENSRNOT00000047289	-2.350932	0.001446	481
Mapkapk3	NM_001012127	2.347711	0.000435	482
Mme	NM_012608	-2.347602	0.000493	483
Thsd7b	NM_001191669	-2.346032	0.000622	484
RGD1306739	NM_001134576	-2.345951	0.001057	485

Ntng1	NM_001106465	-2.345425	0.001447	486
RGD1563319	ENSRNOT00000027344	2.344648	0.000213	487
	ENSRNOT00000006122	2.344311	0.002575	488
	ENSRNOT00000043852	-2.344082	0.001252	489
Dgkg	ENSRNOT00000002452	-2.34329	0.001012	490
Wnt7b	ENSRNOT00000021196	2.339836	0.001107	491
Pfkfb3	ENSRNOT00000068354	2.338606	0.000487	492
Sectm1b	NM_199082	2.335724	0.000746	493
Isg20	NM_001008510	2.333678	0.000531	494
Ankrd57	NM_001109364	2.331145	0.000587	495
	ENSRNOT00000055984	-2.328087	0.001645	496
Clec5a	NM_001109377	2.325986	0.000481	497
E2f5	ENSRNOT00000014361	2.325841	0.000263	498
Klk10	NM_001004100	-2.325668	0.002559	499
Clybl	NM_001100685	-2.324626	0.000357	500
C1qb	ENSRNOT00000017060	2.324165	0.000713	501
Pgm5	NM_001191953	-2.322792	0.001305	502
Chrm5	ENSRNOT00000008387	-2.321745	0.001375	503
Ppp1r15a	NM_133546	2.317866	0.000447	504
Dusp26	NM_001012352	2.313469	0.002351	505
Cdh9	NM_001168630	-2.312561	0.002078	506
Gp5	ENSRNOT00000040884	2.312193	0.002522	507
Faah	NM_024132	-2.311586	0.001168	508
Rnf125	ENSRNOT00000021136	2.310485	0.002451	509
Pex11a	NM_053487	2.30755	0.002538	510
Calca	NM_001033955	2.306883	0.000722	511
Magee2	NM_001106941	-2.30659	0.001541	512
Apold1	NM_001003403	2.306503	0.002159	513
Efna5	NM_053903	2.305976	0.002187	514
Tmem117	XM_001058576	2.305388	0.000496	515
Slc38a2	ENSRNOT00000039002	2.303832	0.000536	516
Tmem88	NM_001128155	2.303427	0.000452	517
C1qc	NM_001008524	2.301895	0.000854	518
Myd88	ENSRNOT00000018341	2.29928	0.000419	519
Rhoc	NM_001106461	2.295314	0.000129	520
LOC290595	NM_001106063	2.289552	0.001029	521
Samd12	NM_001130562	-2.289133	0.001115	522
Cited2	NM_053698	2.28794	0.00043	523
	ENSRNOT00000064161	-2.287645	0.001213	524
Dcc	ENSRNOT00000045408	-2.287024	0.000288	525
Mycl1	NM_001191763	-2.286782	0.00154	526
	ENSRNOT00000002159	2.286004	0.002092	527
Mafk	NM_145673	2.285503	0.000544	528
Plcd1	ENSRNOT00000045493	2.28501	0.00033	529
Sertad1	NM_001007735	2.280317	0.000523	530
Aass	NM_001100963	-2.278901	0.000441	531
Col12a1	ENSRNOT00000051159	2.270133	0.000264	532
Akap12	NM_057103	2.268145	0.001544	533
Fzd9	NM_153305	-2.267873	0.001395	534
RT1-DMb	NM_198740	2.266772	0.000614	535
Chsy1	NM_001106268	2.264542	0.000404	536
Ar	NM_012502	-2.263105	0.001172	537
Kbtbd5	NM_001108195	2.257641	0.00113	538
Mmd2	NM_001037217	-2.25479	0.002447	539

Arhgap31	NM_001105879	2.254597	0.001004	540
Ril	NM_017062	2.253506	0.000793	541
Fcgr2a	M64368	2.250925	0.001854	542
Fgr	ENSRNOT00000013778	2.250334	0.002505	543
Tacr1	NM_012667	2.250211	0.000412	544
Ninj1	ENSRNOT00000022341	2.249097	0.000416	545
Kcng2	NM_001107372	-2.247356	0.001117	546
Rnf217	NM_001106204	2.246256	0.000449	547
Jun	NM_021835	2.243977	0.000781	548
Phyhip	NM_001017376	-2.243734	0.001591	549
Ca2	ENSRNOT00000013354	-2.242903	0.001054	550
Sdc4	NM_012649	2.237667	0.000689	551
Plk3	NM_022187	2.237526	0.000734	552
Ugt1a5	NM_001039549	2.232692	0.001048	553
Bhlhe22	NM_001108940	-2.232479	0.001579	554
Paqr6	NM_001191077	-2.232475	0.000866	555
Ppp1r1b	NM_138521	-2.232166	0.000986	556
Thbs2	NM_001169138	2.231964	0.001789	557
Txnrd1	NM_031614	2.225358	0.000609	558
Ptger4	ENSRNOT00000017886	2.224257	0.000528	559
Fgf1	NM_012846	2.223807	0.001821	560
Zmiz1	NM_001108393	2.222043	0.000692	561
Siglec5	NM_001106249	-2.221141	0.000566	562
Mmp19	NM_001107159	2.221134	0.000599	563
Zfp455	NM_173314	2.220926	0.00128	564
Kank4	NM_001107947	-2.220734	0.00097	565
Lcat	NM_017024	-2.22013	0.000648	566
Itgax	ENSRNOT00000067658	2.220071	0.001201	567
Tspan2	ENSRNOT00000035605	-2.21591	0.000806	568
Ehd4	ENSRNOT00000010701	2.211825	0.001063	569
Rgs5	ENSRNOT00000003705	-2.2116	0.001414	570
Tead1	NM_001198589	2.209873	0.000319	571
Myo1c	ENSRNOT00000036666	2.207496	0.000748	572
Eif1a	NM_001008773	2.207415	0.000586	573
Vat1	NM_001033683	2.205393	0.000313	574
Tef	NM_019194	-2.205004	0.00024	575
Serpinh1	ENSRNOT00000022983	2.203515	0.000433	576
Chrm1	NM_080773	-2.203403	0.00034	577
Angptl2	ENSRNOT00000022585	2.202655	0.002822	578
Rhbdl1	NM_001191822	-2.202354	0.000658	579
Actn1	ENSRNOT00000068522	2.201828	0.000676	580
LOC100363777	ENSRNOT00000065359	-2.200509	0.000461	581
Btbd16	NM_001017464	2.200239	0.001517	582
Thpo	NM_031133	-2.200139	6.00E-04	583
Fxyd1	NM_031648	-2.199903	0.001499	584
Fmod	NM_080698	-2.198051	0.002024	585
Rbm24	NM_001191100	-2.194542	0.002383	586
Trim65	NM_001135714	2.194056	2.00E-04	587
Tsku	NM_001009965	2.194044	0.001602	588
Tbr1	NM_001191070	-2.192252	0.001802	589
Plce1	NM_053758	2.191559	0.000911	590
Inmt	NM_001109022	2.188205	0.002528	591
Lgals1	ENSRNOT00000013538	2.187612	0.002375	592
Zfand2a	NM_001008363	2.185469	0.002456	593

Gpr37	ENSRNOT00000003593	-2.183183	0.002066	594
Ppp1r18	NM_001126287	2.183066	0.000197	595
Pik3ip1	ENSRNOT00000057738	-2.181786	0.001428	596
Casp7	NM_022260	2.181438	0.000298	597
Glis2	NM_001106978	2.178599	0.000486	598
Fat4	NM_001191705	-2.176868	0.002444	599
Fbxo23	NM_001013138	-2.17539	0.001015	600
Plcd4	NM_080688	-2.175256	0.000883	601
Adam11	NM_001108300	-2.174394	0.00046	602
Aldoc	NM_012497	-2.173352	0.000718	603
Cyp2d4	ENSRNOT00000011880	-2.172663	0.000951	604
Slc26a7	NM_001106638	-2.169494	0.002639	605
Hsph1	NM_001011901	2.169377	0.001663	606
Kif22	NM_001009645	2.167067	0.000736	607
Bgn	NM_017087	2.167044	0.001182	608
Slc14a1	ENSRNOT00000022598	2.166	0.000656	609
Kpna2	NM_053483	2.165556	0.000238	610
Itpr1	NM_001007235	-2.1649	0.00112	611
Stk40	NM_183056	2.163556	0.000443	612
	ENSRNOT00000061065	-2.16266	0.000687	613
Mkx	ENSRNOT00000025623	-2.16106	0.0025	614
RGD1309730	ENSRNOT00000056935	-2.160457	0.000759	615
Ipo5	ENSRNOT00000014859	2.160116	0.000256	616
	ENSRNOT00000015387	2.159862	0.001084	617
Mcm2	NM_001107873	2.159803	0.000567	618
Cdr2	NM_001025682	2.159295	0.002147	619
Ifi47	NM_172019	2.158168	0.000808	620
Olfm3	ENSRNOT00000024243	-2.157775	0.000849	621
Ece1	ENSRNOT00000067616	2.155808	0.000694	622
RGD1562811	XM_243842	2.154824	0.0026	623
Aen	NM_001108487	2.154434	0.000453	624
Kpna2	NM_053483	2.152798	0.000309	625
Ier2	NM_001009541	2.151421	0.001359	626
Kcnip2	NM_020094	-2.147813	0.001075	627
Icam5	NM_001172079	-2.146748	0.001019	628
Tlcd1	ENSRNOT00000016840	-2.14489	0.000297	629
Slc26a2	ENSRNOT00000024374	2.142184	0.001294	630
Cd9	NM_053018	2.14168	0.000581	631
LOC690930	XM_001078193	2.139716	0.001159	632
Mcm6	NM_017287	2.13927	0.001987	633
Tagln	ENSRNOT00000024030	2.138398	0.00298	634
Fa2h	NM_001135583	-2.136073	0.002499	635
Furin	ENSRNOT00000015521	2.133693	0.000198	636
Runx3	NM_130425	2.131907	0.002309	637
Il10rb	NM_001107111	2.131678	0.000181	638
Myh1	NM_001135158	2.130192	0.00057	639
Hdac11	NM_001106610	-2.129739	0.000359	640
Atp10d	ENSRNOT00000003146	2.129423	0.000753	641
Sla	NM_178097	2.128847	0.003047	642
Rhoj	NM_001008320	2.126932	0.003038	643
Grm1	NM_001114330	-2.125055	0.001906	644
Cited1	ENSRNOT00000004263	2.124262	0.001487	645
Itgb1	NM_017022	2.123304	0.000286	646
Usp53	ENSRNOT00000066581	2.120222	0.001417	647

Ceacam10	NM_173339	2.11734	0.002032	648
Chdh	NM_198731	2.11529	0.002264	649
Itga4	NM_001107737	-2.110303	0.000643	650
Eprs	NM_001024238	2.108935	0.000225	651
Fam107a	ENSRNOT00000061158	-2.107838	0.002597	652
Errfi1	NM_001014071	2.107086	0.002138	653
LOC100365061	ENSRNOT00000060322	-2.102426	0.000579	654
LOC100365061	ENSRNOT00000060322	-2.102426	0.000579	655
	ENSRNOT00000033811	-2.101745	0.000594	656
Mafb	NM_019316	2.101709	0.001157	657
Ercc1	NM_001106228	2.100007	0.000244	658
Gal3st3	NM_001024290	-2.099947	0.000519	659
Vamp5	NM_053555	2.09824	0.001307	660
Pion	NM_001107845	2.097689	0.000891	661
Sv2c	ENSRNOT00000024459	2.097105	0.002501	662
Folh1	ENSRNOT00000018592	-2.093954	0.000362	663
Bach1	NM_001107113	2.093498	0.000655	664
	ENSRNOT00000016138	2.093017	0.002334	665
Gpr4	NM_001025680	2.090454	0.000636	666
RGD1304963	ENSRNOT00000011832	2.090452	0.001178	667
Lrrn2	NM_001177368	-2.090364	0.001729	668
	ENSRNOT00000009770	-2.087884	0.000824	669
Rbms1	NM_001012184	2.085544	0.001197	670
	ENSRNOT00000011720	-2.08536	0.000978	671
RGD1563533	ENSRNOT00000055621	2.08517	0.001611	672
Itpka	NM_031045	-2.084472	0.001164	673
Thnsl2	NM_001009658	-2.083378	0.000634	674
Gria2	NM_017261	-2.082697	0.001983	675
Nadkd1	ENSRNOT00000022455	-2.082292	0.000464	676
Pdp1	ENSRNOT00000067823	2.082071	0.000972	677
Arhgap12	NM_001107357	-2.081022	0.001379	678
MGC114464	BC097418	-2.080676	0.000506	679
Mmp13	NM_133530	2.07969	0.002272	680
LOC500413	NM_001171802	-2.078936	0.002094	681
S1pr1	NM_017301	-2.077459	0.00025	682
Clic4	ENSRNOT00000024464	2.077404	0.000406	683
Rgs10	ENSRNOT00000027375	-2.077124	0.001954	684
Atf4	ENSRNOT00000065304	2.076288	0.000372	685
Ttll12	NM_001135922	2.074669	0.000549	686
Tp53	NM_030989	2.073429	0.000574	687
Jak2	ENSRNOT00000021217	2.073331	0.000798	688
Fam70a	NM_182822	-2.0724	0.001574	689
	ENSRNOT00000059588	-2.071436	0.001078	690
Mtmr11	NM_001191096	2.07023	0.000386	691
Fhit	NM_021774	-2.070208	0.000814	692
Srgap1	NM_001191784	2.06998	0.00104	693
Tgif1	ENSRNOT00000021534	2.069037	0.001071	694
	ENSRNOT00000036564	2.068948	0.001145	695
Iffo2	NM_001134703	2.066961	0.000898	696
Dennd2c	NM_001191569	2.066531	0.001708	697
Ptprc	NM_138507	2.062295	0.001838	698
Nrbp2	NM_001135007	-2.06071	0.000539	699
Irf1	ENSRNOT00000010968	2.060437	0.000458	700
Slc6a1	NM_024371	-2.060099	0.000409	701

Cntn6	NM_013225	-2.057311	0.001639	702
Asrgl1	ENSRNOT00000027459	-2.057058	0.000764	703
Wnt4	ENSRNOT00000018064	-2.055502	0.002119	704
Parp3	NM_001008328	2.052796	0.000348	705
LOC691414	ENSRNOT00000045621	-2.051495	0.001304	706
Icam1	NM_012967	2.048878	0.002267	707
Map6d1	NM_001108844	-2.046767	0.00256	708
Rapgefl1	BC091361	-2.046538	0.001576	709
Mmp14	NM_031056	2.046004	0.001137	710
Slc10a4	NM_001008555	2.042987	0.002496	711
Arid5a	ENSRNOT00000059916	2.042395	0.001831	712
Ntm	NM_017354	-2.04175	0.002593	713
Rftn2	ENSRNOT00000058841	-2.041596	0.000343	714
Frem2	NM_001245978	-2.04107	0.000504	715
Fam69c	XM_574179	-2.039166	0.00031	716
Bcan	NM_001033665	-2.039151	0.000879	717
LOC501738	NM_001167664	2.037793	0.001103	718
Lrtm2	NM_001109430	-2.036381	0.002489	719
S100a3	ENSRNOT00000016034	2.036377	0.001634	720
Slc39a1	NM_001134577	2.03597	0.000128	721
Fads2	NM_031344	-2.034964	0.000943	722
Cspg5	NM_019284	-2.03251	0.000863	723
Arhgef40	ENSRNOT00000015331	2.031267	0.000573	724
Scn2b	ENSRNOT00000021819	-2.031235	0.000448	725
Trerf1	NM_001108199	-2.030079	0.000254	726
Stom	NM_001011965	2.028986	0.000532	727
Kdm6b	NM_001108829	2.028753	0.001176	728
LOC10036331C	ENSRNOT00000025495	2.02784	0.000647	729
Bcl3	NM_001109422	2.02422	0.000508	730
Kcne4	NM_212526	2.023909	0.000537	731
RGD1564053	NM_001109259	-2.02349	0.000365	732
Zcchc16	XM_003754834	-2.02196	0.001736	733
Il4ra	NM_133380	2.02098	0.001024	734
Klhl21	NM_001107996	2.020079	0.000865	735
Cdc42ep4	NM_001107063	-2.01837	0.000966	736
Acbd4	NM_001012013	-2.01704	0.0013	737
LOC10036423E	ENSRNOT00000065554	-2.016867	0.00094	738
	ENSRNOT00000021988	2.0166	0.000726	739
Midn	NM_001191577	2.016324	0.000424	740
Dot1l	NM_001108733	2.015787	0.000858	741
Trip10	NM_053920	2.014894	0.001185	742
Tet3	ENSRNOT00000015296	2.013825	0.000941	743
	ENSRNOT00000058364	-2.013819	0.002175	744
RGD1559613	NM_001109138	-2.012271	0.000677	745
Lpar4	NM_001106940	-2.011157	0.000926	746
Gulp1	NM_001013171	-2.009467	0.00093	747
Shroom2	NM_001047893	-2.009083	0.000776	748
LOC295528	ENSRNOT00000012638	-2.009074	0.001249	749
Obfc2a	NM_001014216	2.008944	0.00247	750
Spred3	NM_001173344	2.008414	0.001237	751
Creb3l1	NM_001005562	2.008232	0.002834	752
Bak1	ENSRNOT00000000576	2.007766	0.000993	753
Des	NM_022531	2.007656	0.00122	754
Prkcdbp	NM_134449	2.007395	0.001638	755

Crem	NM_001110860	2.006937	0.000987	756
RGD1307119	NM_001013975	-2.006584	0.00202	757
Nkain1	XM_001062503	2.005041	0.000751	758
Litaf	NM_001105735	2.004668	0.000856	759
Spry4	NM_001106150	2.00344	0.002311	760
Tnfrsf10b	NM_001108873	2.003047	0.001469	761
Abhd1	NM_001008520	-2.002809	0.000527	762
Ucn2	NM_133385	2.001369	0.002651	763
Rps6ka3	NM_001192004	2.001316	0.001214	764
Wasf1	NM_001025114	-2.001216	0.001133	765
Hk2	NM_012735	2.000292	0.000644	766
Adamts7	NM_001047101	1.999768	0.00127	767
Alox5ap	NM_017260	1.998844	0.002133	768
Diras2	NM_001169578	-1.998504	0.000524	769
Gprc5d	NM_001109254	1.998302	0.001883	770
Rdh10	NM_181478	1.997711	0.000456	771
Caly	NM_138915	-1.997479	0.000401	772
LOC10091249€	XM_003751082	-1.995321	0.000397	773
Bend7	NM_001191834	1.99455	0.001514	774
Ptrf	NM_001105841	1.994234	0.001985	775
Nppc	NM_053750	-1.99396	0.00118	776
Hspa2	NM_021863	1.993563	0.001967	777
LOC691666	XM_003754628	-1.99197	0.000953	778
Acsf2	NM_001034951	-1.989276	0.000267	779
Phkg1	NM_031573	-1.988931	0.000561	780
Rnf19b	NM_001108003	1.988157	0.000473	781
4-Sep	NM_001011893	-1.985809	0.001476	782
Ppp4r1	ENSRNOT00000066793	1.985453	0.000334	783
Lmna	ENSRNOT00000026917	1.984824	0.000645	784
Phospho1	NM_001105833	1.984568	0.001264	785
	ENSRNOT00000043131	-1.98384	0.002462	786
Mvp	NM_022715	1.983697	0.000705	787
Panx1	NM_199397	1.983254	0.001593	788
Sppl2b	NM_001014200	1.983161	0.000318	789
Ddx21	NM_001037201	1.982598	0.000706	790
Plod2	NM_175869	1.981519	0.000979	791
Tmem8a	NM_001106991	1.977212	0.000641	792
	ENSRNOT00000042423	-1.976709	0.000968	793
	ENSRNOT00000057950	-1.974413	0.00064	794
Gstm7	ENSRNOT00000025689	-1.972709	0.000602	795
Tmem14a	ENSRNOT00000037489	-1.972559	0.000679	796
Apobec1	NM_012907	1.971315	0.002816	797
Eif2c2	ENSRNOT00000011898	1.971314	0.000552	798
Egfl6	NM_001108254	-1.969075	0.000827	799
Gria1	NM_031608	-1.967269	0.001382	800
Chac1	NM_001173437	1.966319	0.001897	801
Cflar	NM_001033864	1.966202	0.00134	802
Axl	ENSRNOT00000028131	1.962658	0.000652	803
Gpr155	NM_001107811	-1.960167	0.001835	804
Gpc5	NM_001107285	-1.959042	0.000794	805
LOC10036474€	ENSRNOT00000045742	1.958663	0.000995	806
Stx11	NM_001025638	1.95844	0.000611	807
Galntl6	NM_001135756	-1.955037	0.000502	808
Elmo1	NM_001108415	1.954504	0.00161	809

Mpped1	NM_001130569	-1.95399	0.002041	810
Uck2	NM_001102408	1.953197	0.000444	811
	ENSRNOT00000055950	1.952884	0.002178	812
Ankrd63	ENSRNOT00000056450	-1.952691	0.001253	813
Irak2	NM_001025422	1.952648	0.001526	814
Camk2g	NM_133605	-1.952564	0.000475	815
Zdhhc23	ENSRNOT00000034122	-1.950932	0.002741	816
Tmem38a	ENSRNOT00000064060	-1.950722	0.000378	817
Slc2a1	ENSRNOT00000064452	1.950595	0.001466	818
Hephl1	ENSRNOT00000043669	1.950049	0.002544	819
Oxnad1	NM_001107295	1.949189	0.000723	820
	ENSRNOT00000014120	-1.949096	0.002058	821
RGD1563072	ENSRNOT00000029395	-1.947223	0.00138	822
Mad2l1bp	NM_001009699	1.947055	0.0011	823
Etv1	NM_001163156	-1.945793	0.002023	824
Elovl2	NM_001109118	-1.945379	0.001581	825
Cntn2	ENSRNOT00000012190	-1.944094	0.002153	826
Rhoq	ENSRNOT00000020822	1.943893	0.001614	827
Jph3	NM_001107437	-1.943405	0.001396	828
Iqgap1	NM_001108489	1.943191	0.001734	829
Serinc5	ENSRNOT00000065131	-1.942343	0.002712	830
Amigo2	NM_182816	-1.942333	0.001509	831
Slc9a4	ENSRNOT00000065234	-1.941538	0.001833	832
LOC691995	NM_001103353	1.941048	0.002389	833
Sorbs3	NM_001005762	-1.939517	0.000596	834
Adk	NM_012895	-1.939294	0.000742	835
Rab20	NM_001109535	1.937621	0.002369	836
Calhm2	NM_001008306	-1.937561	0.00176	837
LOC499330	NM_001024292	-1.937367	0.000276	838
Arntl2	NM_133391	1.935257	0.002608	839
Ddx21	NM_001037201	1.934826	0.000784	840
Itgal	ENSRNOT00000024404	1.934783	0.001037	841
Grin2a	NM_012573	-1.933775	0.00229	842
RGD1311458	NM_001009678	1.932888	0.000702	843
Pycr1	ENSRNOT00000054949	1.932567	0.00136	844
Chn2	ENSRNOT00000012655	-1.931732	0.001682	845
Tp53bp2	ENSRNOT00000004330	-1.931693	0.000516	846
Cdc40	NM_001108538	-1.931562	0.001334	847
Papss2	NM_001106375	1.931124	0.001321	848
Gabrd	NM_017289	-1.930071	0.001571	849
B4galt1	NM_053287	1.925111	0.000632	850
Atp10b	XM_001067759	-1.924887	0.002075	851
Chrd	ENSRNOT00000002394	-1.922923	0.000997	852
Trim16	NM_001135033	1.920995	0.00215	853
Scube1	NM_001134884	1.920185	0.002744	854
Tmem43	ENSRNOT00000010895	1.920044	0.000436	855
Pelo	NM_001007634	1.919645	0.001094	856
Ltbr	NM_001008315	1.919254	0.001376	857
Rcan1	NM_153724	1.918543	0.000721	858
Trim59	ENSRNOT00000013987	-1.917704	0.000498	859
Galnt4	NM_001025053	1.917064	0.000445	860
Camkv	ENSRNOT00000025311	-1.916829	0.000613	861
Slit1	ENSRNOT00000034758	-1.915645	0.001298	862
Asphd2	NM_001009716	-1.914861	0.000927	863

Hrk	NM_057130	-1.914651	0.002549	864
Cdk6	NM_001191861	1.914468	0.002081	865
Fbln1	NM_001127547	-1.913974	0.002771	866
Zc3h12c	NM_001108146	1.913796	0.001559	867
Pkd2	NM_001191934	1.913194	0.002319	868
Gabra5	ENSRNOT00000014573	-1.912493	0.002585	869
Tet1	ENSRNOT00000000302	-1.911758	0.000606	870
Kcnn2	ENSRNOT00000022530	-1.911286	0.00137	871
Adm	NM_012715	1.91103	0.00193	872
Fbl	NM_001025643	1.91033	0.002936	873
Fcgr3a	ENSRNOT00000047434	1.910217	0.002581	874
Heatr1	NM_001108418	1.909696	0.000501	875
Fgf2	NM_019305	1.9085	0.001425	876
Gstk1	NM_181371	-1.906849	0.000874	877
Vwa5a	NM_198755	1.906445	0.001686	878
Homer1	NM_031707	1.905039	0.002166	879
Scg2	NM_022669	1.904456	0.001116	880
Scn8a	ENSRNOT00000066049	-1.904158	0.000382	881
Cdh10	NM_001168631	-1.903509	0.002171	882
RGD1566319	BC166423	1.901479	0.001256	883
Cad	NM_001105710	1.901024	0.001261	884
Omg	NM_001005898	-1.89956	0.00083	885
Hrsp12	ENSRNOT00000007430	-1.89855	0.002365	886
Atp1b2	NM_012507	-1.898409	0.000639	887
Phka1	ENSRNOT00000041138	-1.897719	0.001247	888
Acot13	NM_001106111	-1.896867	0.001837	889
Fam84b	XM_235349	-1.895518	0.002097	890
Gcnt2	NM_001001511	1.895054	0.001445	891
	GENSCAN00000036702	-1.894752	0.001286	892
F3	NM_013057	1.89425	0.002012	893
RGD1560470	ENSRNOT00000040995	-1.894069	0.000756	894
P2ry6	NM_057124	1.893441	0.000801	895
Nav2	NM_138529	1.893388	0.001315	896
Park2	ENSRNOT00000050014	-1.893372	0.001041	897
Ccdc86	ENSRNOT00000028405	1.893064	0.002003	898
Pcdh8	NM_022868	1.892758	0.002121	899
Gfra1	NM_012959	1.890296	0.001595	900
Kctd16	NM_001172155	-1.889189	0.00181	901
Gpld1	NM_001100512	-1.889158	0.001129	902
Pmp2	ENSRNOT00000032059	1.889132	0.002761	903
Cdc42ep4	NM_001107063	-1.887832	0.001681	904
Ddah1	NM_022297	1.886949	0.002046	905
Impdh2	NM_199099	1.886244	0.000575	906
Itga1	NM_030994	1.885974	0.000884	907
Rnd3	ENSRNOT00000006111	1.885899	0.002258	908
Iqsec2	ENSRNOT00000004620	-1.885476	0.001244	909
Ptpn2	ENSRNOT00000063770	1.88476	0.000468	910
Gstm1	ENSRNOT00000047139	-1.882211	0.000616	911
Lrrfip2	NM_001024761	1.881884	0.001319	912
Cdkl1	NM_001025121	-1.880751	0.001855	913
Cml5	NM_080884	-1.880523	0.00121	914
RGD1304810	NM_001107314	-1.880372	0.00049	915
Sdcbp2	NM_001025692	1.880329	0.00272	916
Cbfb	NM_001013191	1.87867	0.002441	917

Ppapdc1b	NM_001109411	1.87827	0.001339	918
Plod3	NM_178101	1.878183	0.001113	919
Gpr65	NM_001106751	1.878054	0.002673	920
Pstpip1	NM_001106824	1.877671	0.002335	921
Gpc4	NM_001014108	-1.876379	0.002015	922
Myh9	ENSRNOT00000007398	1.87625	0.001483	923
Kcnq3	NM_031597	-1.875814	0.002455	924
RT1-CE11	NM_001008834	1.874577	0.001673	925
Lmo7	ENSRNOT00000032917	-1.87289	0.001655	926
Aff1	NM_001107206	1.872704	0.000541	927
Pltp	NM_001168543	-1.872509	0.000761	928
Pxmp2	ENSRNOT00000056680	-1.87212	0.001017	929
Tyw1	NM_001107137	1.871196	0.000374	930
Fam132b	ENSRNOT00000051810	1.871162	0.002884	931
Slc7a5	NM_017353	1.870877	0.000624	932
Id1	NM_012797	1.870314	0.001184	933
Srm	NM_053464	1.868362	0.000739	934
Rtkn2	NM_001107625	-1.866827	0.000651	935
Sh3bp4	ENSRNOT00000026312	-1.866819	0.001007	936
Bcar1	NM_012931	1.866594	0.001403	937
	ENSRNOT00000001017	1.86625	0.001205	938
Ervfrd-1	NM_001024239	-1.866058	0.001112	939
Ptpru	NM_001191575	-1.865367	0.001044	940
Trib3	NM_144755	1.864295	0.001732	941
	ENSRNOT00000047388	-1.863282	0.001867	942
Lrrc3b	XM_002725036	-1.862759	0.001678	943
Hddc3	NM_001107528	-1.862333	0.002563	944
Clec1a	NM_001109253	-1.862171	0.00155	945
Dync1i1	ENSRNOT00000013184	-1.86184	0.000894	946
Ptprd	ENSRNOT00000066046	-1.861138	0.000335	947
LOC100359498	XM_003752497	1.861029	0.001326	948
Odc1	NM_012615	1.859637	0.000619	949
Jun	NM_021835	1.859527	0.00056	950
Slc37a1	NM_001011944	1.859328	0.002142	951
	ENSRNOT00000061132	-1.858039	0.000598	952
Dse	ENSRNOT00000001093	1.857777	0.00147	953
Itprip	ENSRNOT00000017296	1.85674	0.001912	954
Aldh6a1	NM_031057	-1.85662	0.000944	955
	GENSCAN00000016382	1.856613	0.002227	956
Heatr5a	ENSRNOT00000035309	1.85562	0.000735	957
Mat2a	NM_134351	1.855205	0.000842	958
Tbxas1	ENSRNOT00000010796	1.854135	0.002263	959
Eln	NM_012722	1.852381	0.002054	960
Spry2	NM_001012046	1.851308	0.00168	961
P4hb	NM_012998	1.849144	0.001778	962
Celsr2	NM_001191110	-1.849086	0.000867	963
Ifitm2	NM_030833	1.848338	0.001904	964
Ttll9	NM_001014051	-1.847803	0.002186	965
Pros1	NM_031086	1.845982	0.000916	966
Crtap	NM_001108785	1.841489	0.000719	967
Cd84	NM_001192006	-1.84135	0.001573	968
Fads6	NM_001107064	-1.841256	0.001	969
	ENSRNOT00000043099	-1.84084	0.001949	970
Sncb	NM_080777	-1.840634	0.000904	971

Podxl	NM_138848	1.840133	0.001491	972
LOC686781	XM_002728700	1.839826	0.001217	973
Gfpt2	NM_001002819	1.839727	0.001104	974
Syt2	NM_012665	-1.839644	0.001619	975
RT1-CE4	NM_001008842	1.839418	0.002848	976
Vangl2	NM_001105969	-1.838507	0.00189	977
Pcdhb13	ENSRNOT00000027172	-1.836457	0.002223	978
Smad1	ENSRNOT00000025079	1.836404	0.000888	979
Grpr	ENSRNOT00000005559	-1.836246	0.002176	980
Ppara	NM_013196	-1.835432	0.00078	981
Mlkl	ENSRNOT00000036972	1.835388	0.002163	982
Slc39a6	NM_001024745	1.832799	0.000328	983
	ENSRNOT00000004637	1.832302	0.00265	984
	ENSRNOT00000067452	-1.832207	0.001709	985
Vasp	NM_001108475	1.832163	0.000675	986
Csda	NM_031979	1.831973	0.001365	987
Cbr4	NM_182672	-1.831884	0.002766	988
Snrpa	NM_001008303	1.831305	0.000558	989
Rbpj	NM_001106631	1.831016	0.000896	990
Relt	NM_001108495	1.829931	8.00E-04	991
Rnf180	NM_001134986	-1.829625	0.000623	992
Ppp2r1b	NM_001025418	1.829403	0.00021	993
Ncf4	NM_001127304	1.82914	0.001787	994
Plek	NM_001025750	1.827473	0.002762	995
Hcls1	NM_001011898	1.825395	0.001554	996
LOC10036478З	ENSRNOT00000042647	1.825314	0.000381	997
Cdc42ep1	NM_001079700	1.825071	0.001399	998
Syt4	NM_031693	1.824964	0.002526	999
Cav2	NM_131914	1.824762	0.000797	1000
Fam189b	ENSRNOT00000027828	-1.82452	0.001091	1001
Mapk1ip1l	NM_001108373	1.824039	0.000234	1002
	ENSRNOT00000012248	-1.822968	0.002316	1003
Samd9l	XM_003753866	-1.822531	0.001858	1004
Pgr	ENSRNOT00000066956	-1.821817	0.002129	1005
LOC361128	NM_001047104	1.821529	0.001768	1006
Esam	NM_001004245	1.818996	0.000701	1007
Sfxn5	NM_153298	-1.818892	0.000846	1008
Golga7b	XM_219889	1.818718	0.003026	1009
Pdcl3	NM_001025709	1.818217	0.000958	1010
Plxna3	NM_001107581	-1.817152	0.00068	1011
Slc9a3r1	ENSRNOT00000004351	-1.816949	0.001952	1012
S100a13	NM_001191607	-1.816743	0.00169	1013
Dis3	NM_001127483	1.815788	0.001507	1014
Tars	NM_001006976	1.815442	0.000546	1015
Pmaip1	ENSRNOT00000025362	1.814131	0.002668	1016
Pla2g4a	NM_133551	1.813058	0.002628	1017
Zyx	NM_053761	1.812356	0.001443	1018
	ENSRNOT00000039420	1.811296	0.001895	1019
Tmod3	NM_001011997	1.811215	0.000822	1020
Eri1	NM_001014143	1.810985	0.000399	1021
Slc31a2	NM_001033693	1.81006	0.001265	1022
Srgn	NM_020074	1.809659	0.00086	1023
Aldh5a1	ENSRNOT00000050919	-1.809244	0.000769	1024
Sec24d	BC168946	1.808949	0.000892	1025

Kbtbd3	NM_001108121	-1.808941	0.001053	1026
Slc20a1	ENSRNOT00000025294	1.808526	0.002189	1027
Ptpro	NM_017336	1.808049	0.000472	1028
Cdh12	XM_229369	-1.806996	0.001088	1029
Lrg1	NM_001009717	1.806281	0.00037	1030
Mpp2	NM_053513	-1.806256	0.001136	1031
Nim1	ENSRNOT00000060518	-1.806115	0.001484	1032
Ptpn6	ENSRNOT00000065416	1.80565	0.00175	1033
Dtl	ENSRNOT00000005628	1.804975	0.000923	1034
Dennd3	XM_001072997	1.804876	0.000565	1035
Slitrk2	ENSRNOT00000015401	-1.804841	0.001209	1036
Prr18	NM_001108464	-1.804401	0.000698	1037
LOC501224	BC079060	-1.801639	0.001538	1038
Phlda2	NM_001100521	-1.80134	9.00E-04	1039
Cntn5	NM_053746	-1.800773	0.001558	1040
Mcc	NM_001170534	1.800505	0.001557	1041
Rhbdd1	NM_001024891	1.800419	0.001109	1042
Fut9	NM_053465	-1.800328	0.002625	1043
Nol12	NM_001012747	1.800155	0.001263	1044
Shbg	NM_012650	-1.799033	0.000562	1045
LOC10035986	XM_003754011	1.798736	0.002141	1046
Tec	ENSRNOT00000003125	1.798572	0.001653	1047
Otud1	XM_001074167	1.798568	0.000955	1048
Pecam1	NM_031591	1.798524	0.001222	1049
Drp2	ENSRNOT00000039864	-1.797978	0.001121	1050
Speg	NM_001108802	-1.797056	0.000983	1051
Fkbp10	NM_001014120	1.795355	0.001462	1052
Spred2	NM_001047094	1.794785	0.001441	1053
Pxdc1	NM_001025719	1.794763	0.001233	1054
Nt5c1a	NM_001107976	-1.794418	0.002371	1055
Lrfn5	NM_001108024	-1.794295	0.000773	1056
Gpt	ENSRNOT00000050556	-1.793747	0.001006	1057
Myo6	ENSRNOT00000064856	-1.793339	0.001563	1058
Ankrd28	ENSRNOT00000026680	1.793193	0.000627	1059
LOC100360891	ENSRNOT00000042721	-1.793034	0.002323	1060
Kcnq5l	ENSRNOT00000018552	-1.792188	0.002645	1061
Pex5l	NM_173152	-1.791344	0.001631	1062
Gpr111	ENSRNOT00000033075	1.789997	0.001584	1063
Cav2	NM_131914	1.789056	0.000789	1064
Llgl1	ENSRNOT00000005366	1.788697	0.000786	1065
	ENSRNOT00000006968	-1.788516	0.001288	1066
Spsb2	NM_001009660	1.787908	0.001841	1067
RGD1305222	NM_001169102	1.78785	0.000969	1068
Mcee	NM_001106341	-1.787535	0.001783	1069
Otub2	NM_001108053	-1.787375	0.001367	1070
Fam5b	NM_173115	-1.786905	0.002612	1071
	ENSRNOT00000041261	-1.786675	0.001171	1072
Sprn	NM_001031845	-1.786457	0.000499	1073
Aldh18a1	NM_001108524	1.785916	0.000914	1074
Mgat4c	NM_001135814	-1.785859	0.001963	1075
Mars	NM_001127659	1.785785	0.000708	1076
Pxn	NM_001012147	1.785571	0.001578	1077
LOC10036022	ENSRNOT00000060396	-1.78517	0.002317	1078
Yars	NM_001025696	1.785006	0.000696	1079

Capn2	NM_017116	1.784844	0.000956	1080
	ENSRNOT00000040395	-1.78225	0.002404	1081
Arrdc4	NM_001047853	1.781548	0.001726	1082
RGD1565119	ENSRNOT00000048106	-1.780898	0.002688	1083
Spi1	NM_001005892	1.780331	0.001397	1084
Crispld2	NM_138518	1.778807	0.001764	1085
	ENSRNOT00000039741	-1.778136	0.000949	1086
	ENSRNOT00000055381	-1.778044	0.00102	1087
Sp140	NM_001012133	1.777931	0.002555	1088
Ftsj3	NM_001012014	1.777012	0.001216	1089
	GENSCAN00000001985	-1.776929	0.002211	1090
C1qtnf4	NM_001107745	-1.776896	0.001098	1091
LOC498368	BC079124	-1.775486	0.002082	1092
Hif1a	NM_024359	1.775435	0.000714	1093
LOC100364307	ENSRNOT00000046075	-1.775239	0.002321	1094
Nek6	ENSRNOT00000066510	1.774563	0.000483	1095
Plxdc1	NM_001107046	-1.774549	0.002694	1096
Psme2	NM_017257	1.772232	0.000771	1097
Polr1e	NM_001107938	1.772121	0.00234	1098
Antxr1	NM_001044249	-1.769793	0.001371	1099
Cables2	ENSRNOT00000008279	1.769501	0.002436	1100
	ENSRNOT00000068680	-1.769487	0.001241	1101
Lgr5	NM_001106784	-1.769158	0.001536	1102
Gar1	NM_001024306	1.768642	0.000962	1103
RGD1307443	NM_001197023	-1.768345	0.001665	1104
Phkb	NM_001014152	-1.767993	0.001763	1105
Nop56	NM_001025732	1.76491	0.000981	1106
Ripk1	NM_001107350	1.764046	0.000861	1107
Gdpd2	NM_001106944	-1.76381	0.001125	1108
Pwwp2b	NM_001108507	-1.763753	0.001859	1109
Abhd14b	NM_001007664	-1.763621	0.000945	1110
Csf1	ENSRNOT00000025222	1.76292	0.002704	1111
Mybbp1a	ENSRNOT00000021134	1.760943	0.001122	1112
Txndc16	ENSRNOT00000008372	-1.759972	0.000727	1113
	ENSRNOT00000056133	-1.75814	0.001099	1114
Isoc1	ENSRNOT00000026706	-1.757979	0.002472	1115
Cyp4x1	NM_145675	-1.757736	0.000805	1116
	ENSRNOT00000055579	-1.756246	0.001603	1117
Tln1	NM_001039025	1.755933	0.000635	1118
Klhl5	NM_001047093	-1.755859	0.000533	1119
Psat1	NM_198738	-1.754903	0.002424	1120
Trpc4	NM_080396	-1.754609	0.002494	1121
Vipr2	NM_017238	-1.754196	0.001893	1122
Crh	ENSRNOT00000016953	1.754181	0.002711	1123
LOC314942	ENSRNOT00000039318	-1.754069	0.001031	1124
Klhl13	ENSRNOT00000067114	-1.754004	0.000991	1125
Klf10	ENSRNOT00000008350	1.75351	0.002926	1126
Tph1	NM_001100634	1.752818	0.001627	1127
Car11	NM_175708	-1.752493	0.001162	1128
Itpr1	NM_001007235	-1.752446	0.002414	1129
RGD1306058	ENSRNOT00000022203	1.752242	0.002181	1130
Jakmip3	NM_001163277	-1.752059	0.001313	1131
Bcl11a	ENSRNOT00000009421	-1.751737	0.002247	1132
Aspg	ENSRNOT00000017454	1.751483	0.000852	1133

Rgnef	NM_001108542	-1.750374	0.000875	1134
Lrrk2	ENSRNOT00000005438	-1.750003	0.002776	1135
Gstt1	NM_053293	-1.749974	0.000395	1136
rnf141	NM_001001800	-1.749785	0.001825	1137
Arhgap20	ENSRNOT00000035989	-1.749606	0.000887	1138
Nos3	NM_021838	1.748873	0.001659	1139
Fcer1g	NM_001131001	1.748779	0.002067	1140
Lrrc23	NM_001013165	-1.748558	0.001959	1141
Pcbp3	NM_001011945	1.748277	0.000816	1142
Ankrd34a	NM_001024980	-1.747745	0.002557	1143
Ska3	NM_001108379	1.747606	0.000948	1144
RGD1309651	ENSRNOT00000023721	-1.746642	0.001548	1145
Ggta1p	NM_145674	1.746617	0.002867	1146
Ckap4	ENSRNOT00000010601	1.746165	0.001521	1147
Ptprn	ENSRNOT00000026654	1.745916	0.000932	1148
Lgmn	NM_022226	1.745856	0.001141	1149
Fn3krp	NM_001107077	-1.745477	0.000744	1150
	ENSRNOT00000027244	-1.744611	0.001528	1151
Ndp	NM_001108814	-1.744369	0.001212	1152
Mcm7	ENSRNOT00000001825	1.74425	0.001023	1153
Dhrs3	ENSRNOT00000021155	-1.743726	0.001062	1154
Amot	ENSRNOT00000009669	-1.743628	0.000835	1155
Sipa1	ENSRNOT00000028137	1.743406	0.00038	1156
Egflam	NM_001108938	-1.742971	0.00069	1157
Paqr7	ENSRNOT00000022713	-1.741504	0.000947	1158
Ripk2	NM_001191865	1.74121	0.002275	1159
Nudt2	NM_207596	-1.739613	0.001624	1160
Ptpn12	NM_057115	1.738304	0.000569	1161
Frmd8	NM_001008348	1.738096	0.000577	1162
Abhd8	NM_001107301	-1.737816	0.001449	1163
Osgin2	XM_232798	1.73718	0.001705	1164
Npl	NM_001013984	1.735531	0.001504	1165
B3gat1	NM_054003	-1.735146	0.002259	1166
	ENSRNOT00000059629	-1.734449	0.000836	1167
Cdk2ap1	NM_001113751	1.733843	0.001003	1168
Oxct1	NM_001127580	-1.733642	0.001067	1169
Grm5	NM_017012	-1.733447	0.001255	1170
Echs1	NM_078623	-1.732955	0.001623	1171
Morc4	BC158847	1.732441	0.002037	1172
Slc25a23	NM_001106873	-1.731683	0.000323	1173
Myo1f	NM_001108076	1.73144	0.0024	1174
Vhl	ENSRNOT00000013727	1.731326	0.000839	1175
Chst7	NM_207600	-1.730723	0.001193	1176
Trank1	NM_001191799	1.730601	0.002564	1177
Rassf1	ENSRNOT00000037375	1.7305	0.000693	1178
Pygb	NM_013188	-1.729538	0.000709	1179
Cdc25c	NM_001107396	1.72936	0.001431	1180
Ddx39a	NM_053563	1.727313	0.002234	1181
Cdc25a	NM_133571	1.726676	0.001409	1182
Lonp1	NM_133404	1.726491	0.000344	1183
Utp20	NM_001191779	1.726146	0.00182	1184
Prss23	NM_001007691	1.725767	0.001163	1185
Bai3	NM_001106898	-1.725691	0.001151	1186
Smagp	ENSRNOT00000040205	1.72506	0.00081	1187

Pyroxd2	NM_001004261	-1.724558	0.002452	1188
Krt18	NM_053976	-1.724499	0.001743	1189
Anxa3	ENSRNOT00000065429	1.72418	0.001111	1190
LOC689271	XM_001070212	-1.722887	0.001869	1191
LOC100360712	ENSRNOT00000048256	-1.722523	0.00188	1192
Cyb5a	ENSRNOT00000020446	-1.722147	0.000683	1193
Mob3a	NM_001108734	1.721485	0.002809	1194
LOC680222	BC162040	-1.721286	0.000841	1195
	ENSRNOT00000030681	-1.719728	0.001722	1196
Ttc9	NM_001134731	1.71961	0.001065	1197
Tpt1	NM_053867	1.719302	0.000595	1198
Zfp521	NM_001107403	1.719067	0.002569	1199
Tbc1d30	ENSRNOT00000024679	-1.719007	0.001798	1200
RGD1565648	ENSRNOT00000055510	1.718959	0.002219	1201
Smarca2	ENSRNOT00000016740	-1.718671	0.001008	1202
	ENSRNOT00000037022	-1.718192	0.002519	1203
Appl2	NM_001108741	-1.71765	0.000913	1204
Rplp0	NM_022402	1.716703	0.000711	1205
Slc12a5	NM_134363	-1.716672	0.000871	1206
Plekhd1	NM_001127566	-1.715004	0.002605	1207
Lrrc73	NM_001024360	-1.714543	0.001146	1208
Arhgef2	NM_001012079	-1.714225	0.001174	1209
Wdr46	NM_212491	1.71416	0.001772	1210
	ENSRNOT00000031380	-1.713344	0.002221	1211
Adrbk2	NM_012897	-1.713265	0.001649	1212
Dck	ENSRNOT00000004440	-1.713028	0.00233	1213
Fzd10-ps1	XM_003752587	-1.712566	0.001032	1214
Slc24a3	NM_053505	-1.712243	0.001738	1215
Lgi1	ENSRNOT00000020411	-1.711792	0.001011	1216
Aldh1l2	NM_001191778	1.711196	0.002596	1217
LOC10036555€	ENSRNOT00000045724	-1.709818	0.000782	1218
Trmt61a	NM_001007706	1.709365	0.001291	1219
Abcb9	NM_022238	-1.708538	0.002197	1220
	ENSRNOT00000058076	-1.707754	0.002307	1221
Nefm	NM_017029	-1.706995	0.002541	1222
Sorl1	NM_053519	-1.706759	0.00273	1223
Klf12	NM_001107281	-1.706009	0.001356	1224
Nrsn2	NM_001109561	-1.705958	0.002205	1225
Orai1	NM_001013982	1.705608	0.001609	1226
Snx18	NM_001107652	1.705049	0.001126	1227
Ggh	NM_012960	-1.704982	0.00241	1228
Zfp385d	ENSRNOT00000038449	-1.704701	0.0014	1229
Inpp5a	ENSRNOT00000054890	1.704581	0.000454	1230
S100a1	NM_001007636	-1.704425	0.001105	1231
Aatf	NM_053720	1.704063	0.00216	1232
Mcm5	ENSRNOT00000019677	1.703968	0.002053	1233
Wdr43	NM_001037791	1.702951	0.00129	1234
Inpp5j	ENSRNOT00000026293	-1.702502	0.001941	1235
Gpcpd1	NM_198779	-1.702179	0.000937	1236
Cnn3	NM_019359	1.699934	0.000465	1237
Pnkd	NM_001134750	-1.698916	0.001552	1238
RGD1308772	ENSRNOT00000013780	-1.697514	0.00217	1239
Dzip1	ENSRNOT00000038611	-1.69741	0.000804	1240
Sema5b	NM_001107091	1.697375	0.002242	1241

Gbp2	NM_133624	1.696711	0.002859	1242
Ezh2	NM_001134979	1.696191	0.001275	1243
	ENSRNOT00000039710	-1.69593	0.00275	1244
LOC691600	ENSRNOT00000046551	-1.694491	0.002387	1245
RGD1304827	NM_001009437	-1.693623	0.001706	1246
Gas7	NM_053484	-1.693411	0.000671	1247
8-Sep	NM_001107002	-1.693272	0.000826	1248
Shc1	NM_001164060	1.692657	0.000853	1249
	ENSRNOT00000010548	-1.69263	0.002572	1250
Eif3b	NM_001031640	1.692274	0.000653	1251
Pld2	NM_033299	-1.689913	0.001328	1252
St6galnac3	NM_019123	-1.689864	0.002691	1253
Pi4k2b	NM_001005883	1.689836	0.00114	1254
RGD1305834	ENSRNOT00000055611	1.689717	0.000905	1255
Ctnna2	NM_001106598	-1.688052	0.001028	1256
Gria4	NM_017263	-1.686946	0.000919	1257
Pcna	ENSRNOT00000028887	1.686213	0.001251	1258
Prtg	NM_001037651	-1.685262	0.001347	1259
Hyal2	NM_172040	1.684605	0.002652	1260
Fgf13	NM_053428	-1.683832	0.001876	1261
Mis18bp1	NM_001109531	1.68378	0.002453	1262
Plxdc2	NM_001108422	-1.682252	0.002403	1263
Tpp1	NM_031357	-1.681198	0.00194	1264
Aprt	NM_001013061	1.677326	0.001374	1265
Tmem218	NM_001008325	-1.676769	0.001223	1266
Thoc4	NM_001109602	1.675424	0.001191	1267
C1qa	NM_001008515	1.674656	0.001805	1268
LOC100362572	XM_002727697	-1.674437	0.002044	1269
Lrrc59	NM_001008280	1.674019	0.00085	1270
Paqr8	NM_001014099	-1.672099	0.00228	1271
Plcb1	ENSRNOT00000051184	-1.672093	0.001035	1272
Eaf1	NM_001107293	1.671929	0.00157	1273
RGD1565616	NM_001109206	-1.671878	0.000704	1274
Il2rg	NM_080889	1.671609	0.002957	1275
B4galt5	NM_001108608	1.670828	0.001327	1276
Lrrc16a	NM_001191692	-1.670231	0.000924	1277
Arl4a	ENSRNOT00000005800	1.669856	0.002282	1278
Limd1	NM_001112737	1.669623	0.002115	1279
Pgcp	NM_031640	-1.669563	0.002006	1280
Syngap1	NM_181092	-1.668999	0.000626	1281
Paqr5	NM_001014092	-1.668626	0.002634	1282
Prok2	NM_001037541	1.668536	0.00207	1283
Trex1	NM_001024989	1.668185	0.001296	1284
Tspo	NM_012515	1.667889	0.002873	1285
LOC100364307	ENSRNOT00000046075	-1.667499	0.002672	1286
Sema4g	NM_001108526	-1.667292	0.001648	1287
Mtfr1	NM_001100977	1.666507	0.002394	1288
Rwdd3	NM_001128152	-1.666366	0.001132	1289
LOC501224	BC079060	-1.664834	0.001816	1290
B3gnt1	NM_001106324	-1.664232	0.00149	1291
Cish	NM_031804	1.664014	0.001513	1292
Tmem150c	NM_001108354	-1.663944	0.002061	1293
Slc16a2	ENSRNOT00000040637	-1.663191	0.001155	1294
Stmn1	NM_017166	-1.662483	0.001308	1295

Irf3	NM_001006969	1.662124	0.002297	1296
Tmem154	NM_001108553	1.662033	0.001336	1297
Serpini1	ENSRNOT00000013904	-1.661153	0.001158	1298
Zfp259	NM_001137646	1.660127	0.001724	1299
Ivd	ENSRNOT00000013829	-1.658649	0.000303	1300
Ak4	NM_017135	-1.658297	0.002431	1301
Itpripl2	NM_001127303	1.658066	0.001773	1302
Nfkb1	ENSRNOT00000068674	1.657867	0.002943	1303
Rars	NM_001105777	1.656548	0.000662	1304
Aven	NM_001107757	1.655156	0.00047	1305
Rit2	NM_001013060	-1.654516	0.001351	1306
Fam126b	BC097998	1.654056	0.001503	1307
Ndufb4	NM_001037338	-1.652641	0.002485	1308
Erbb4	ENSRNOT00000019283	-1.652113	0.001532	1309
Fnip2	XM_227287	1.652026	0.000931	1310
Phactr3	NM_214459	-1.651501	0.001808	1311
	ENSRNOT00000064618	-1.649932	0.001248	1312
LOC497978	NM_001039341	-1.648117	0.001271	1313
Cmpk2	NM_001108017	-1.647334	0.001545	1314
Flt3	NM_001100822	-1.646625	0.002373	1315
Aldh2	ENSRNOT00000001816	-1.64599	0.002504	1316
Mrpl45	NM_001105834	1.645979	0.00082	1317
Adck3	NM_001013185	-1.645709	0.001797	1318
Pabpc1	ENSRNOT00000012775	1.645445	0.000582	1319
Twf1	NM_001008521	1.645226	0.000899	1320
Pdlim5	NM_053326	1.644832	0.000915	1321
Kcna2	ENSRNOT00000050149	-1.643656	0.001889	1322
Tmem238	XM_574354	1.643424	0.001962	1323
Fgfr1	ENSRNOT00000029284	1.643413	0.00145	1324
RGD1308059	NM_001025022	1.643274	0.003015	1325
RGD1559864	ENSRNOT00000047893	-1.643154	0.001942	1326
Rasgef1a	ENSRNOT00000042277	-1.642968	0.001405	1327
Chpt1	ENSRNOT00000007305	-1.641865	0.001834	1328
	ENSRNOT00000067417	-1.641555	0.002125	1329
Fads3	NM_173137	1.641537	0.002906	1330
Npm1	NM_012992	1.641321	0.002313	1331
Pa2g4	NM_001004206	1.641132	0.000697	1332
Zfhx2	NM_001098803	-1.640991	0.00192	1333
Muc1	NM_012602	1.639554	0.000738	1334
Ston2	NM_001135874	-1.638959	0.001668	1335
Fnbp1	NM_138914	-1.637897	0.001422	1336
Cebpg	ENSRNOT00000028703	1.637394	0.00159	1337
LOC690550	XM_003753274	-1.637269	0.002099	1338
Mgst3	ENSRNOT00000005719	-1.63697	0.002236	1339
Tlr7	NM_001097582	1.636623	0.002357	1340
Crlf1	NM_001106074	-1.636316	0.002714	1341
Klhl23	NM_001134504	-1.635119	0.002348	1342
Fndc3b	NM_001191704	1.635028	0.002807	1343
Vcl	NM_001107248	1.634771	0.001991	1344
Rbfox3	ENSRNOT00000004524	-1.634216	0.002695	1345
Csrp3	ENSRNOT00000019311	1.634082	0.002254	1346
Acsbg1	ENSRNOT00000016104	-1.634068	0.001879	1347
Zswim4	NM_001107163	1.63358	0.001273	1348
Adipor1	NM_207587	1.632688	0.000356	1349

Fam105a	BC107649	1.632497	0.002105	1350
Sez6l2	NM_001107550	-1.632066	0.001284	1351
LOC10036069€	ENSRNOT00000060304	-1.63198	0.002401	1352
Usp16	NM_001100501	1.631375	0.001728	1353
Nr1h3	ENSRNOT00000018154	1.631375	0.001864	1354
Tmem123	NM_001014205	1.630983	0.002615	1355
Rasal1	NM_001108335	-1.630902	0.002104	1356
Add3	NM_001164103	-1.629988	0.001657	1357
Pbx2	NM_001002828	-1.629849	0.001278	1358
	ENSRNOT00000057802	1.629794	0.002152	1359
Vps29	NM_001105932	-1.629472	0.001279	1360
Trpt1	NM_001106331	1.628937	0.002476	1361
Nat8l	NM_001191681	-1.628208	0.00227	1362
Ppapdc2	NM_001034854	-1.627914	0.002579	1363
Abhd3	NM_001106162	-1.627163	0.001412	1364
Rest	ENSRNOT00000002837	1.626398	0.001108	1365
Amph	ENSRNOT00000017102	-1.626321	0.001868	1366
RGD1562655	ENSRNOT00000038868	1.62622	0.002027	1367
Snx7	NM_001012083	1.626024	0.002746	1368
Serpine2	NM_019197	-1.625101	0.000881	1369
Cidec	NM_001024333	1.624842	0.001066	1370
Abhd14a	ENSRNOT00000016056	-1.624411	0.001588	1371
Wnt7a	NM_001100473	-1.623886	0.000935	1372
LOC10036069€	ENSRNOT00000060304	-1.623276	0.00221	1373
Stk10	NM_019206	1.622913	0.001828	1374
Dok4	NM_001108438	-1.622862	0.001972	1375
Robo2	NM_032106	-1.622732	0.001674	1376
	ENSRNOT00000006961	1.622602	0.003006	1377
LOC100360017	ENSRNOT00000002752	1.622431	0.001457	1378
Zc3h6	NM_001107772	-1.622188	0.001387	1379
Skap2	NM_130413	1.621496	0.002551	1380
	ENSRNOT00000067720	1.621049	0.00173	1381
LOC10036132€	ENSRNOT00000034945	-1.620703	0.001324	1382
Ddx18	NM_001006996	1.62047	0.000664	1383
	ENSRNOT00000063809	-1.619588	0.001916	1384
Elmod1	NM_001191579	-1.618992	0.00186	1385
Cetn2	ENSRNOT00000065164	-1.618302	0.002127	1386
Sh2b2	NM_053669	1.617003	0.002686	1387
Pycard	ENSRNOT00000026699	1.615602	0.002576	1388
Hn1l	NM_001013182	1.615238	0.003037	1389
Adam9	ENSRNOT00000041330	1.614878	0.000603	1390
Tnpo1	NM_001100692	1.614735	0.001056	1391
Sgpl1	NM_173116	1.614485	0.001384	1392
Slc18a2	ENSRNOT00000011983	1.614193	0.001964	1393
LOC690349	NM_001109581	1.613849	0.000909	1394
Nxph1	NM_012994	-1.612965	0.001228	1395
Jdp2	NM_053894	-1.612944	0.00214	1396
Sipa1l1	NM_139330	-1.612863	0.001086	1397
Pinx1	ENSRNOT00000016027	1.612415	0.00172	1398
Vwa3a	NM_001198653	-1.612306	0.001437	1399
Tmed5	ENSRNOT00000000083	1.612258	0.002008	1400
Nudt14	NM_001106760	-1.611891	0.002132	1401
Ocln	ENSRNOT00000024674	-1.611466	0.002676	1402
Ier5l	NM_001025041	1.61014	0.000928	1403

Mrto4	NM_001106697	1.609758	0.001092	1404
Kcnrg	ENSRNOT00000032514	-1.609656	0.00231	1405
Faim2	NM_144756	-1.608238	0.00076	1406
Nampt	ENSRNOT00000013043	1.608022	0.001946	1407
Actl6a	NM_001039033	1.607616	0.000961	1408
Msmo1	ENSRNOT00000044171	1.607603	0.001353	1409
Dbf4	NM_001191748	1.6064	0.001533	1410
Rab3b	ENSRNOT00000010645	-1.605992	0.002192	1411
Pygo1	NM_001191117	-1.605513	0.001385	1412
Ttll7	ENSRNOT00000055318	-1.605118	0.0012	1413
Gcdh	NM_001108896	-1.604659	0.000922	1414
Matk	NM_021859	-1.604595	0.001666	1415
Ttc30b	NM_001127607	-1.604232	0.001776	1416
Fermt2	NM_001011915	1.604125	0.000767	1417
	ENSRNOT00000040854	-1.602986	0.00148	1418
LOC500877	NM_001047963	-1.602399	0.001843	1419
Astn1	NM_001170603	-1.60236	0.000862	1420
Scp2	ENSRNOT00000015420	-1.602342	0.001531	1421
Pde1b	NM_022710	-1.602242	0.001839	1422
Ddx3x	ENSRNOT00000039551	1.602002	0.002286	1423
LOC501224	BC079060	-1.601793	0.001822	1424
Ncald	NM_001024371	-1.601759	0.001512	1425
Cdv3	NM_001014097	1.60174	0.002532	1426
Disp2	NM_001107759	-1.601495	0.000765	1427
Ckmt1b	NM_001012738	-1.601025	0.002151	1428
Dgat1	ENSRNOT00000039795	1.600694	0.00167	1429
Vav1	NM_012759	1.60067	0.002727	1430
Haus8	NM_001024971	1.600652	0.001516	1431
Cstb	NM_012838	1.600517	0.001757	1432
Alg10	NM_139101	1.600458	0.000973	1433
Dram	NM_001173427	1.59997	0.001224	1434
Arf6	NM_024152	1.599674	0.00062	1435
LOC10036183C	ENSRNOT00000017592	1.599203	0.002698	1436
Ctnna2	NM_001106598	-1.598504	0.002604	1437
St3gal4	NM_203337	1.598362	0.002022	1438
RGD1563365	XM_002729760	1.59791	0.002134	1439
Chst11	NM_001108079	1.597497	0.001582	1440
LOC10036399З	ENSRNOT00000066278	1.597359	0.001751	1441
Slc7a6	NM_001107424	1.597305	0.00075	1442
RGD1311756	NM_001173557	-1.597261	0.001661	1443
LOC10036237З	ENSRNOT00000064610	-1.596559	0.002735	1444
RGD1312026	NM_001108149	1.59643	0.00304	1445
Snx32	ENSRNOT00000036745	-1.596315	0.001101	1446
LOC100360712	ENSRNOT00000048256	-1.595858	0.001259	1447
Nme6	NM_001191884	1.595598	0.001753	1448
Trip11	ENSRNOT00000007004	1.595557	0.002408	1449
Mdp1	NM_001106039	-1.595323	0.001907	1450
Hspd1	ENSRNOT00000019912	1.595083	0.00249	1451
Camta2	NM_001105801	-1.594981	0.001357	1452
Tpcn1	NM_139332	-1.594133	0.001809	1453
Slc10a3	NM_001024368	1.59342	0.001761	1454
Ptprf	ENSRNOT00000027271	1.592676	0.001702	1455
Prkab1	ENSRNOT00000001508	1.591156	0.002537	1456
LOC10036200٤	ENSRNOT00000007042	-1.591022	0.002542	1457

Ddhd2	ENSRNOT00000065770	-1.590995	0.001932	1458
Nmbr	NM_012799	-1.590964	0.00141	1459
Stxbp2	NM_031126	1.590746	0.001154	1460
Gab2	ENSRNOT00000016361	1.590685	0.002419	1461
Pde2a	NM_001143847	-1.590321	0.001771	1462
Necab2	NM_133415	-1.590257	0.001061	1463
Dysf	ENSRNOT00000064536	1.589203	0.0017	1464
LOC100364984	ENSRNOT00000038103	-1.588944	0.002666	1465
Ptk2b	ENSRNOT00000030007	-1.588033	0.001626	1466
Lyar	NM_001011911	1.587081	0.001033	1467
Tmc5	NM_001012216	-1.587027	0.001926	1468
Cdk4	NM_053593	1.586876	0.001455	1469
Irf2bpl	NM_001012470	1.586631	0.001717	1470
LOC690349	NM_001109581	1.586477	0.002156	1471
Hibadh	NM_022243	-1.585811	0.001245	1472
Slc16a4	NM_001013913	-1.585604	0.002457	1473
Slc23a2	NM_017316	1.58382	0.001979	1474
Bnip2	NM_001106835	1.583795	0.00143	1475
Gprc5b	NM_001106304	-1.583253	0.001852	1476
Tmem120a	NM_001010945	1.583122	0.002074	1477
Pus3	NM_001108134	1.582983	0.00099	1478
Zdhhc18	ENSRNOT00000009235	1.582778	0.002007	1479
Cenpi	NM_012955	1.582498	0.001913	1480
LOC691414	ENSRNOT00000051446	-1.582468	0.002609	1481
Fam46a	NM_001106844	1.58193	0.002033	1482
Helb	XM_001081027	1.58176	0.002085	1483
LOC100359977	ENSRNOT00000056197	1.581561	0.001689	1484
Tmem25	NM_001109528	-1.581449	0.002631	1485
Vwa5b2	NM_001134535	-1.581412	0.002384	1486
Ppp1r14b	NM_172045	1.581089	0.001274	1487
Prpf3	NM_001108559	1.58098	0.001096	1488
Sox8	NM_001106989	-1.580619	0.001406	1489
Aig1	NM_001134425	-1.58044	0.001565	1490
Parvb	NM_001134780	1.579894	0.001128	1491
Isg20l2	NM_001007741	1.579773	0.001842	1492
LOC304239	ENSRNOT00000049215	-1.577614	0.002754	1493
	ENSRNOT00000007303	1.577463	0.001348	1494
Mgat5b	NM_001107068	-1.577273	0.001806	1495
Olfm1	NM_053573	-1.577218	0.002352	1496
Bid	ENSRNOT00000016776	1.57687	0.000774	1497
Hars	NM_001025414	1.575833	0.001586	1498
	ENSRNOT00000041337	-1.575146	0.001149	1499
Numbl	ENSRNOT00000057213	1.574824	0.002516	1500
Erc2	NM_170787	1.574738	0.002707	1501
Erc2	NM_170787	1.574738	0.002707	1502
Erc2	NM_170787	1.574738	0.002707	1503
Dhx37	ENSRNOT00000030823	1.574473	0.002449	1504
Ebna1bp2	NM_001008721	1.574048	0.001978	1505
Slfn2	NM_001107031	1.573892	0.001134	1506
Ung	NM_001013124	1.573731	0.002973	1507
Thoc6	NM_024384	1.573263	0.001059	1508
Ak2	NM_030986	1.573148	0.002353	1509
Elf1	NM_053520	1.572117	0.001824	1510
Ppm1l	NM_001107681	1.572053	0.002661	1511

Klhdc1	ENSRNOT00000064668	-1.571746	0.001714	1512
Fau	NM_001012739	1.571256	0.001454	1513
Dnajc25	NM_001025021	1.571227	0.002095	1514
Fjx1	NM_001108955	-1.571004	0.002183	1515
Dock8	NM_001037793	1.570729	0.002616	1516
Slco2b1	NM_080786	-1.570643	0.002545	1517
Pabpc4	NM_001100538	1.570517	0.002071	1518
RGD1565059	NM_001127562	-1.570389	0.001917	1519
Mad2l2	NM_001012106	1.569858	0.001309	1520
Epb4.1l5	NM_001012023	-1.569664	0.001508	1521
Rps13	NM_130432	1.56913	0.000998	1522
Epb41l3	NM_053927	-1.569024	0.00152	1523
Psma5	ENSRNOT00000026928	1.567897	0.002167	1524
LOC100364307	ENSRNOT00000059076	-1.567714	0.001704	1525
Trpc5	ENSRNOT00000008299	-1.567115	0.002062	1526
Bcl2l1	NM_001033671	1.567075	0.002523	1527
Tpcn2	NM_001107566	1.567028	0.001303	1528
Ran	ENSRNOT00000001247	1.566694	0.002174	1529
RGD1308396	ENSRNOT00000027604	1.566667	0.001073	1530
Nol3	ENSRNOT00000020908	1.566466	0.002123	1531
Srcin1	NM_019378	-1.566318	0.001025	1532
RGD1308165	NM_001108664	1.566139	0.001986	1533
Hspa9	NM_001100658	1.565911	0.000778	1534
Tap1	ENSRNOT00000000529	1.565509	0.001408	1535
Col4a2	ENSRNOT00000057461	1.565395	0.001765	1536
Tceal1	NM_001009675	-1.565106	0.002509	1537
Tspyl4	NM_001012075	-1.564369	0.000663	1538
Pdcd6ip	NM_001029910	1.564227	0.001095	1539
Nme3	ENSRNOT00000021650	-1.564207	0.000878	1540
Sgsm3	NM_198787	1.563907	0.002294	1541
Lrrc4	NM_001037336	-1.563829	0.0027	1542
Mob1a	NM_001033891	1.563769	0.001344	1543
RGD1359378	NM_001007658	-1.563405	0.002719	1544
Ralgps2	ENSRNOT00000006511	-1.563287	0.001748	1545
	ENSRNOT00000034542	-1.562793	0.001795	1546
Prkra	NM_001024780	-1.562142	0.00142	1547
Frmpd4	NM_001106960	-1.562103	0.002409	1548
Snx2	NM_001106135	1.561785	0.001685	1549
Atp13a3	ENSRNOT00000066907	1.561737	0.001082	1550
LOC100360712	ENSRNOT00000048256	-1.56172	0.002718	1551
Oxnad1	NM_001107295	1.561036	0.000976	1552
Bop1	NM_001024250	1.56077	0.00117	1553
Ppil1	ENSRNOT00000068527	1.560685	0.001501	1554
Slc2a13	ENSRNOT00000021153	-1.560454	0.001632	1555
	ENSRNOT00000044597	-1.560061	0.002565	1556
Chchd10	NM_001007008	-1.559555	0.00107	1557
Oxct1	NM_001127580	-1.559045	0.001383	1558
Ctu2	NM_001037094	1.558948	0.00195	1559
Rela	NM_199267	1.558914	0.001236	1560
Sdad1	NM_001006958	1.558849	0.001235	1561
Serf1	XM_577983	-1.558714	0.001426	1562
Samd4a	NM_001107254	1.55862	0.002425	1563
Mob1a	NM_001033891	1.557974	0.001612	1564
Utp3	NM_001012036	1.55779	0.001945	1565

Ddx54	NM_001191548	1.557462	0.00101	1566
	ENSRNOT00000048377	1.557021	0.002331	1567
Gas6	NM_057100	-1.556548	0.000985	1568
Sdr9c7	ENSRNOT00000005919	-1.556524	0.001994	1569
Slc26a1	ENSRNOT00000000047	-1.556315	0.001522	1570
Nacc2	NM_001100533	-1.556286	0.000994	1571
Esyt1	NM_017249	1.556255	0.002226	1572
Plekhg2	BC169013	1.556	0.002225	1573
Unc5a	NM_022206	-1.555665	0.001049	1574
Imp4	NM_001009700	1.555289	0.000832	1575
Fam151b	BC167070	-1.555249	0.001418	1576
Zcchc12	NM_001014065	-1.554682	0.002213	1577
Dclk1	NM_053343	1.554659	0.002517	1578
Cacnb3	NM_012828	-1.554255	0.002469	1579
Nalcn	NM_153630	-1.554048	0.001744	1580
Ppan	ENSRNOT00000027973	1.553563	0.000848	1581
Hs6st1	ENSRNOT00000019966	1.553143	0.001961	1582
Pecr	NM_133299	-1.552716	0.001518	1583
Vegfa	NM_031836	1.552376	0.001331	1584
Dlg3	ENSRNOT00000003741	-1.552249	0.001933	1585
Rgs12	ENSRNOT00000045068	-1.552194	0.001299	1586
Cntfr	NM_001003929	-1.552159	0.001759	1587
Pgm1	NM_017033	-1.551656	0.001175	1588
	ENSRNOT00000010609	-1.55099	0.002073	1589
Scamp2	NM_023955	1.550179	0.002429	1590
Phf10	NM_001024747	1.549976	0.002991	1591
Fam59b	NM_001191874	-1.549696	0.001598	1592
Slc7a1	NM_013111	1.54943	0.001322	1593
Tpx2	ENSRNOT00000010851	1.547605	0.00174	1594
Rab3a	NM_013018	-1.54673	0.001045	1595
Gmpr2	NM_001013036	1.546638	0.001442	1596
Glud1	ENSRNOT00000013788	-1.54564	0.001569	1597
Ezh1	NM_001107051	-1.545235	0.001898	1598
	ENSRNOT00000060398	-1.545179	0.002725	1599
Tecpr1	ENSRNOT00000001347	-1.544795	0.001153	1600
Hcn2	NM_053684	-1.544723	0.001511	1601
	ENSRNOT00000004800	-1.544707	0.002377	1602
Pxk	NM_182821	1.544347	0.000812	1603
LOC10036419C	ENSRNOT00000008688	-1.543976	0.002011	1604
Pcgf5	NM_001129882	1.543299	0.002588	1605
Tbck	NM_001134513	-1.542311	0.002229	1606
Eif2c1	NM_001191765	-1.541309	0.001784	1607
Zer1	NM_001100707	-1.540087	0.001617	1608
Taf9	NM_001012463	1.540011	0.001257	1609
Cd151	NM_022523	1.539938	0.001684	1610
Naa50	NM_001105881	1.538738	0.001138	1611
	ENSRNOT00000065894	1.538508	0.000725	1612
Cacna2d2	ENSRNOT00000021218	-1.53784	0.001687	1613
Sft2d2	NM_001034011	1.537521	0.001953	1614
Vars	NM_053292	1.53749	0.001439	1615
Psd	NM_134370	-1.537003	0.002246	1616
Wdr74	NM_001109569	1.536473	0.002281	1617
Ctsl1	NM_013156	1.535996	0.002086	1618
Atic	NM_031014	1.535719	0.000918	1619

Rnf5	NM_001109025	-1.534848	0.000977	1620
Eif2s2	NM_199380	1.534846	0.001529	1621
RGD1304884	NM_001107436	1.534243	0.001424	1622
Ttc7	NM_001100756	1.533958	0.002399	1623
Large	NM_001108439	-1.533873	0.001767	1624
Esrrg	ENSRNOT00000003489	-1.533424	0.00236	1625
Naa50	NM_001105881	1.533274	0.002083	1626
Taf5l	NM_001107442	1.533137	0.001525	1627
Pphln1	NM_001108992	1.532891	0.001287	1628
Dcaf10	NM_001107935	1.532767	0.000485	1629
	ENSRNOT00000055887	1.532577	0.002252	1630
Fry	ENSRNOT00000041365	-1.531304	0.001693	1631
	ENSRNOT00000065617	-1.531077	0.001924	1632
RGD1560891	ENSRNOT00000066401	-1.531055	0.002214	1633
Tsc22d4	NM_001044284	1.530563	0.002599	1634
Mpp3	ENSRNOT00000055191	-1.530556	0.002584	1635
Nsun2	NM_001108403	1.530132	0.001562	1636
RGD1562626	NM_001253859	-1.529321	0.002589	1637
Rrp1b	ENSRNOT00000001583	1.529074	0.002432	1638
	ENSRNOT00000061526	-1.528996	0.002343	1639
Chrna1	NM_024485	1.528075	0.001599	1640
Tmem93	ENSRNOT00000026168	1.52803	0.00096	1641
Afaf	ENSRNOT00000029441	1.527418	0.001938	1642
Arhgap39	NM_173122	-1.527208	0.002396	1643
Dync2li1	NM_001013940	-1.526982	0.001069	1644
Utp15	NM_001107647	1.526833	0.002757	1645
Atic	NM_031014	1.526527	0.001166	1646
LOC10036283€	ENSRNOT00000058937	1.526439	0.00223	1647
Gmfg	ENSRNOT00000026891	1.525712	0.001812	1648
Extl2	NM_001100704	-1.524964	0.002493	1649
Btbd2	ENSRNOT00000025391	-1.524607	0.001267	1650
	ENSRNOT00000035084	1.523856	0.00244	1651
Rps3	NM_001009239	1.523677	0.002797	1652
Gpr75	NM_001109096	-1.522603	0.001343	1653
Arhgap17	ENSRNOT00000019309	1.522426	0.002862	1654
Ube2g2	NM_001106380	1.522415	0.001899	1655
Hspe1	NM_012966	1.522349	0.00225	1656
Prkcg	ENSRNOT00000019825	-1.52217	0.00253	1657
Shank3	ENSRNOT00000047960	-1.521675	0.002696	1658
Ssx2ip	NM_175597	-1.521469	0.002548	1659
Tmem39a	NM_001013865	1.52135	0.00126	1660
Eif2b1	ENSRNOT00000001373	1.521124	0.00123	1661
Tor3a	NM_001009683	1.520276	0.002909	1662
Neto1	NM_001107371	-1.519961	0.001725	1663
Edn3	ENSRNOT00000009826	-1.519768	0.002161	1664
Sfxn4	NM_001108527	-1.518854	0.002327	1665
Rpl6	NM_053971	1.518727	0.001923	1666
Hnrnpf	NM_001037286	1.518474	0.002266	1667
Cald1	ENSRNOT00000041264	1.518409	0.002468	1668
Snf1lk2	DQ188032	1.518239	0.001826	1669
Ptprd	XM_001067936	-1.517655	0.00219	1670
	ENSRNOT00000066845	-1.517202	0.002064	1671
Tspan33	NM_001109227	-1.517164	0.001478	1672
Fkrp	NM_001025678	-1.516955	0.001497	1673

Galnt1	NM_024373	1.516788	0.001226	1674
Cby1	ENSRNOT00000018787	-1.515909	0.001676	1675
Prkd1	ENSRNOT00000005596	-1.515064	0.002339	1676
Panx2	ENSRNOT00000048501	-1.514973	0.00197	1677
Lhpp	NM_001009706	-1.514317	0.001567	1678
Nip7	ENSRNOT00000027653	1.514213	0.002421	1679
Cmtm3	NM_001106164	1.513987	0.001651	1680
Caskin1	NM_080690	-1.513564	0.001891	1681
Tyw5	NM_001170473	1.51346	0.002998	1682
Cnpy4	NM_001108852	1.513115	0.002767	1683
	ENSRNOT00000058763	1.51288	0.001488	1684
Psmd8	NM_001100831	1.512783	0.001167	1685
Chn1	NM_032083	-1.512085	0.002354	1686
Cnih4	NM_001105981	1.512056	0.00084	1687
Rps20	NM_001007603	1.511987	0.000747	1688
Mapk7	NM_001191547	1.511411	0.002274	1689
Lrrfip1	NM_001014269	1.509978	0.00205	1690
Gstp1	NM_012577	-1.509943	0.001231	1691
LOC691141	ENSRNOT00000041091	1.509651	0.002845	1692
Trmt6	NM_001107779	1.50889	0.002647	1693
Zfp593	NM_001106689	1.508744	0.001316	1694
Scn2a1	NM_012647	-1.507558	0.002397	1695
Ica1l	ENSRNOT00000041546	-1.506171	0.002535	1696
Rpl23	NM_001007599	1.505845	0.001052	1697
Cnppd1	NM_199112	1.5056	0.001747	1698
Pfkm	ENSRNOT00000013374	-1.505334	0.000895	1699
Plec	ENSRNOT00000045149	1.505205	0.002333	1700
Selv	NM_001166396	-1.504954	0.001998	1701
Ascc2	NM_001109091	1.504448	0.002179	1702
Alkbh5	NM_001191643	1.503873	0.002533	1703
	ENSRNOT00000031893	-1.503871	0.002136	1704
Prdx6	ENSRNOT00000034583	-1.503415	0.001495	1705
	ENSRNOT00000036931	1.503032	0.002101	1706
Ptprs	NM_019140	-1.502989	0.001014	1707
Gnl3	ENSRNOT00000033677	1.502967	0.001463	1708
Uhrf1bp1l	NM_001108753	1.502885	0.002568	1709
Sh2d5	ENSRNOT00000020069	1.502212	0.000668	1710
Slc3a2	NM_019283	1.501733	0.001716	1711
Dyrk3	NM_001024767	1.500707	0.002636	1712
Akr1c19	NM_001100576	-1.500598	0.00242	1713
Nell2	NM_031070	-1.500588	0.001846	1714
Hs2st1	NM_001100518	1.500106	0.002715	1715
Pate4	NM_001108757	4.644672	0.003808	1716
Nps	XM_002728814	4.077022	0.003268	1717
	ENSRNOT00000012067	3.387216	0.003467	1718
Epcam	NM_138541	3.179326	0.004	1719
Rxfp2	NM_001012475	3.06544	0.003538	1720
Galr1	NM_012958	3.011638	0.003969	1721
Cd74	NM_013069	2.906864	0.003363	1722
Pld5	NM_001191674	-2.794129	0.003016	1723
Egr2	NM_053633	2.745066	0.003679	1724
Rgs4	NM_017214	2.712403	0.003496	1725
Ecel1	NM_021776	2.628777	0.003729	1726
Rln3	ENSRNOT00000007775	2.595383	0.003904	1727

Camk4	NM_012727	-2.473498	0.003209	1728
Sgpp2	NM_001191811	-2.458675	0.003454	1729
Sec16b	NM_053571	2.415012	0.003056	1730
LOC680415	ENSRNOT00000059368	2.412246	0.004055	1731
Kcnj16	NM_053314	-2.37943	0.002969	1732
Pafah1b3	ENSRNOT00000027774	2.351007	0.003875	1733
	ENSRNOT00000044814	-2.307367	0.002814	1734
Ctsk	NM_031560	2.277841	0.003763	1735
Sorl1	NM_053519	-2.250894	0.002952	1736
Nfkbiz	NM_001107095	2.241058	0.003478	1737
Slc24a2	NM_031743	-2.201504	0.003455	1738
Gpr88	ENSRNOT00000037068	2.185524	0.004067	1739
LOC363060	NM_001014209	2.177781	0.003747	1740
Mettl7a	NM_001037355	-2.170461	0.003068	1741
	ENSRNOT00000042498	-2.159461	0.002885	1742
Hspa1a	NM_031971	2.152565	0.003339	1743
Mpped2	ENSRNOT00000065168	-2.128681	0.003288	1744
Eya1	ENSRNOT00000064946	-2.10947	0.003244	1745
Hmgn5	NM_001134706	-2.104548	0.003072	1746
Cryab	NM_012935	2.103014	0.003451	1747
	ENSRNOT00000065035	2.095427	0.004079	1748
Rasl11b	NM_001002830	-2.078531	0.003735	1749
Sphkap	NM_001127492	-2.024988	0.002801	1750
Dpp10	NM_001012205	-2.017499	0.003401	1751
Fzd6	NM_001130536	2.010769	0.003313	1752
Cadps2	ENSRNOT00000068404	-2.008941	0.003149	1753
Trpm3	NM_001191562	-2.006232	0.003444	1754
Npy1r	NM_001113357	-2.005266	0.003599	1755
Klk8	NM_001107509	-1.996433	0.003089	1756
Abcg2	NM_181381	-1.992319	0.003632	1757
Efnb3	NM_001100980	-1.972764	0.002941	1758
Cnp	ENSRNOT00000023872	-1.970648	0.003115	1759
Scn3b	NM_139097	-1.969429	0.003443	1760
Sstr2	ENSRNOT00000003735	-1.966955	0.00292	1761
Plcxd2	NM_001134481	-1.965898	0.003754	1762
Rsph10b	NM_001013867	-1.947019	0.003062	1763
Coro7	NM_001191639	-1.941114	0.003378	1764
Fat3	NM_138544	1.928822	0.003699	1765
Aldh1a1	NM_022407	-1.928819	0.003437	1766
	ENSRNOT00000033772	-1.920638	0.0038	1767
Raver2	NM_001191867	-1.913898	0.00324	1768
	ENSRNOT00000014013	-1.905118	0.003668	1769
Edn1	NM_012548	1.897816	0.003619	1770
	ENSRNOT00000039652	-1.888113	0.003675	1771
Pctp	ENSRNOT00000003295	1.880743	0.003663	1772
Coq10b	NM_001009671	1.877057	0.003522	1773
Pak6	ENSRNOT00000010471	1.873588	0.003847	1774
Syt17	ENSRNOT00000023090	-1.869661	0.00286	1775
RGD1309170	NM_001134579	1.869633	0.003981	1776
Plekhb1	NM_172033	-1.869357	0.003346	1777
Fxyd7	NM_022008	-1.864034	0.003182	1778
	ENSRNOT00000024963	1.85858	0.003193	1779
Pnck	ENSRNOT00000024227	-1.855306	0.00344	1780
RT1-O1	NM_001008856	-1.848126	0.003074	1781

Vwa1	NM_001013938	1.848052	0.003673	1782
Rassf2	ENSRNOT00000028883	-1.838802	0.00348	1783
Rac2	NM_001008384	1.836981	0.003071	1784
Extl1	NM_001107985	-1.835706	0.003479	1785
Tmem176a	NM_001039008	-1.830963	0.003034	1786
Kcnh7	NM_131912	-1.828216	0.00367	1787
Jph1	NM_001106630	-1.823488	0.003318	1788
RGD1564195	ENSRNOT00000049044	-1.811065	0.003153	1789
Pcsk1	NM_017091	1.781661	0.0031	1790
Tas2r135	ENSRNOT00000031921	1.775337	0.00394	1791
Zfp367	NM_001012051	1.772965	0.003285	1792
RGD1561916	ENSRNOT00000017903	-1.772675	0.003208	1793
Dagla	NM_001005886	-1.772475	0.003067	1794
Gpr63	NM_001106640	-1.772422	0.003082	1795
Tsc22d3	ENSRNOT00000048080	-1.772148	0.00325	1796
Mcam	NM_023983	1.766836	0.0035	1797
Scn1a	NM_030875	-1.764184	0.003198	1798
	ENSRNOT00000061017	1.761061	0.003535	1799
LOC497940	NM_001017476	1.759218	0.00338	1800
Mast3	NM_001134796	-1.758543	0.002802	1801
Mx2	NM_134350	-1.754718	0.003486	1802
Chst15	ENSRNOT00000022157	-1.747416	0.003617	1803
Tmem229a	NM_001109480	-1.744395	0.003726	1804
LOC100188984	NM_001134998	-1.741382	0.00284	1805
LOC364653	XM_001056850	-1.733142	0.003261	1806
Sec23b	NM_001108593	1.728968	0.003108	1807
Htra1	NM_031721	1.726612	0.003805	1808
Nkain3	NM_001109540	-1.725786	0.002881	1809
Gpr161	ENSRNOT00000004104	-1.722579	0.002953	1810
Wfdc1	NM_133581	-1.714057	0.002832	1811
Psrc1	NM_001044302	-1.713458	0.003214	1812
Sergef	XM_344891	1.711837	0.003236	1813
	ENSRNOT00000050879	1.711304	0.00334	1814
Tox3	NM_001106171	-1.710905	0.002792	1815
	ENSRNOT00000059478	-1.710404	0.003782	1816
Plekhg3	ENSRNOT00000008573	-1.708074	0.003718	1817
Wnt9a	NM_001105783	-1.707119	0.003595	1818
Cib2	NM_001015010	-1.705896	0.003751	1819
LOC100365923	ENSRNOT00000002991	1.701302	0.003778	1820
Ttyh2	ENSRNOT00000004081	-1.701168	0.003275	1821
Cd53	ENSRNOT00000024344	1.694485	0.003894	1822
Tmem176b	ENSRNOT00000011327	-1.694131	0.003054	1823
Vwf	ENSRNOT00000026643	1.693726	0.003065	1824
Gdnf	NM_019139	1.691003	0.003101	1825
Degs2	NM_001017457	-1.688806	0.003338	1826
Cldn23	NM_001033062	1.687402	0.003144	1827
	ENSRNOT00000015429	1.676266	0.003147	1828
Lyn	NM_030857	1.674916	0.003995	1829
Arhgdib	NM_001009600	1.674623	0.003282	1830
	ENSRNOT00000065093	1.671198	0.003998	1831
Kcnab2	NM_017304	-1.668782	0.003665	1832
Acsl6	ENSRNOT00000030760	-1.667503	0.003779	1833
Mrps12	NM_001106239	1.667332	0.00386	1834
Ltc4s	ENSRNOT00000004359	-1.659025	0.002905	1835

Zfp575	NM_001107489	-1.658986	0.003385	1836
Leprel4	NM_021581	1.657695	0.003433	1837
RGD1566085	XM_001079270	-1.655734	0.002879	1838
Abcb1a	NM_133401	-1.655433	0.003647	1839
LOC10036431З	ENSRNOT00000034041	-1.655103	0.003799	1840
RGD1563613	ENSRNOT00000044779	1.650294	0.003334	1841
Arpc1b	ENSRNOT00000001315	1.648849	0.003312	1842
Camkk1	NM_031662	-1.646113	0.003565	1843
Gchfr	NM_133595	1.645578	0.003396	1844
Uspl1	NM_001198555	1.644337	0.003258	1845
Acadsb	NM_013084	-1.643672	0.002903	1846
Cplx2	ENSRNOT00000000117	-1.642682	0.003492	1847
Sp7	NM_001037632	1.641394	0.004141	1848
Ecm1	NM_053882	1.64071	0.003523	1849
Gpr84	NM_001109509	1.640425	0.00396	1850
Kctd14	ENSRNOT00000016654	1.639899	0.003172	1851
Itga2b	ENSRNOT00000036112	1.638654	0.003382	1852
Ppp1r16b	NM_001191072	-1.637734	0.002914	1853
Fzd2	ENSRNOT00000032944	-1.637256	0.003711	1854
Pik3c2b	NM_001105951	-1.636843	0.002844	1855
	ENSRNOT00000052207	-1.635716	0.00342	1856
Galm	NM_001007704	-1.633015	0.003076	1857
Kcnc1	NM_012856	-1.632216	0.002827	1858
Btbd3	NM_001107782	-1.631857	0.003627	1859
L1cam	NM_017345	-1.626531	0.002937	1860
RGD1563285	XM_001080526	1.626044	0.003519	1861
LOC688801	ENSRNOT00000064470	-1.624323	0.00363	1862
Wee1	NM_001012742	-1.622858	0.003634	1863
Slc5a11	NM_001100482	-1.621251	0.002966	1864
	ENSRNOT00000048826	1.621046	0.00381	1865
Dapk1	NM_001107335	-1.618426	0.002917	1866
Uap1	NM_001191930	1.617113	0.0039	1867
Plekhh2	NM_001191770	1.616488	0.004121	1868
Cd276	NM_182824	1.614926	0.003592	1869
Cenpm	NM_001130504	1.61446	0.003717	1870
Epha6	ENSRNOT00000047310	-1.613723	0.003584	1871
Gjd2	NM_019281	-1.612724	0.003297	1872
	ENSRNOT00000027225	1.611948	0.00316	1873
Sez6	NM_001105754	-1.610364	0.003043	1874
Cd97	NM_001012164	1.609955	0.003192	1875
Plekhh1	ENSRNOT00000014257	-1.608353	0.003319	1876
Cyba	NM_024160	1.605662	0.003114	1877
Nnmt	NM_001106819	1.604396	0.003372	1878
Pdlim7	ENSRNOT00000018899	1.603011	0.003113	1879
LOC10036137б	NM_001270413	1.600602	0.003853	1880
RGD1560383	XM_001081416	-1.600585	0.003676	1881
Map7	NM_001106270	-1.600182	0.002806	1882
RGD1308626	BC088867	-1.600036	0.003138	1883
Arl5c	NM_001109046	1.598548	0.003712	1884
Spon1	ENSRNOT00000042088	-1.597225	0.003507	1885
LOC691308	ENSRNOT00000060297	-1.596955	0.00314	1886
Cyp2s1	NM_001107495	1.596701	0.00354	1887
Mpst	ENSRNOT00000000201	-1.595953	0.003758	1888
Ptgr2	NM_001015009	-1.595914	0.002823	1889

Cap2	NM_053874	-1.595541	0.002871	1890
Itpkb	NM_019312	1.595214	0.003324	1891
As3mt	NM_080890	-1.59131	0.003459	1892
Rbl1	NM_001191066	1.590583	0.003683	1893
Bcas3	NM_001173430	-1.59057	0.003551	1894
Ifit2	NM_001024753	-1.589618	0.00351	1895
Ccdc80	NM_022543	-1.588866	0.002956	1896
LOC691984	ENSRNOT00000050826	1.588344	0.00341	1897
Ppp3r1	NM_017309	-1.587986	0.003559	1898
Napa	ENSRNOT00000002044	-1.585962	0.003081	1899
Muc5b	XM_238988	1.585612	0.003811	1900
Dbndd2	NM_001047111	-1.584519	0.00374	1901
Ugdh	ENSRNOT00000003691	1.58438	0.003342	1902
Snai1	NM_053805	1.58183	0.00318	1903
Atp10a	NM_001141935	-1.581784	0.003686	1904
Atp1a2	NM_012505	-1.578611	0.002824	1905
Ier5	NM_001025137	1.576052	0.004038	1906
Slc9a2	NM_001113335	-1.574305	0.003598	1907
Phyh	NM_053674	-1.572239	0.00319	1908
RGD1563441	ENSRNOT00000012061	1.571652	0.00358	1909
Dynlt1	ENSRNOT00000024656	1.569542	0.003456	1910
Plac8	NM_001108353	1.567076	0.003358	1911
Cdk2	NM_199501	1.566378	0.003129	1912
10-Sep	NM_001014033	1.565481	0.003554	1913
Tnr	ENSRNOT00000003407	-1.565279	0.003571	1914
RGD1563888	NM_001108312	-1.564291	0.002795	1915
Bicc1	NM_001108531	1.563793	0.003489	1916
RGD1566215	NM_001106929	-1.563228	0.003186	1917
Stil	ENSRNOT00000010410	1.561607	0.003265	1918
Cldn9	NM_001011889	-1.561102	0.003587	1919
Polr1b	ENSRNOT00000024882	1.55874	0.003425	1920
Nradd	ENSRNOT00000028416	1.558645	0.003468	1921
	ENSRNOT00000048156	1.55778	0.003325	1922
Ppm1h	BC088307	1.557605	0.003953	1923
LOC500827	NM_001024352	1.556636	0.00368	1924
Mt3	NM_053968	-1.556628	0.003655	1925
Sema4d	NM_001170563	-1.553803	0.003137	1926
Acsm5	ENSRNOT00000046025	-1.553334	0.003118	1927
Fut2	NM_031635	-1.552579	0.003771	1928
Pln	ENSRNOT00000000469	-1.552031	0.002837	1929
	ENSRNOT00000035992	-1.550322	0.00329	1930
Vcan	NM_001170558	1.548185	0.003204	1931
Stk17b	ENSRNOT00000016856	1.547827	0.003117	1932
Pim3	NM_022602	1.547696	0.003702	1933
Prrg4	NM_001109203	1.547183	0.004028	1934
Gpr158	NM_001170326	1.546986	0.003255	1935
Tm7sf4	ENSRNOT00000006243	1.546315	0.003403	1936
Tnfrsf26	NM_001108511	1.545021	0.003099	1937
Gnal	NM_001191836	-1.544377	0.003737	1938
Bmp15	NM_021670	-1.543317	0.003272	1939
Calcrl	ENSRNOT00000006462	1.542513	0.003791	1940
Galntl1	NM_001100863	-1.542125	0.003658	1941
Zfp518b	XM_001062122	1.540805	0.00399	1942
Aga	NM_001031641	-1.540177	0.002869	1943

	ENSRNOT00000055358	-1.539437	0.003008	1944
Rgma	NM_001107524	-1.538192	0.003266	1945
Tmem205	NM_001106804	-1.53748	0.003304	1946
Cdh11	NM_053392	-1.536571	0.003426	1947
Spns2	NM_001144991	1.536567	0.004131	1948
Il1rl1	ENSRNOT00000020108	1.536315	0.003708	1949
Scyl3	NM_001191828	1.536207	0.003213	1950
Hey1	NM_001191845	-1.535859	0.003232	1951
Spats2l	NM_001014102	-1.534895	0.003387	1952
Rasl10b	NM_001191648	-1.533989	0.002787	1953
LOC679748	XM_001054317	-1.532788	0.003057	1954
Fam13a	NM_001100862	-1.532145	0.002784	1955
Atl3	NM_001044241	1.531834	0.003111	1956
Ankrd50	NM_001191606	1.531046	0.003051	1957
Mmp15	NM_001106168	-1.530382	0.003333	1958
Zcchc24	NM_001108394	-1.530095	0.00369	1959
Gcnt1	NM_022276	1.529998	0.003405	1960
Sh2d1b2	XM_001065477	1.529816	0.003475	1961
Mal2	NM_198786	-1.528777	0.003434	1962
Ccdc85a	NM_001191553	-1.527143	0.00347	1963
Srebf1	ENSRNOT00000042979	-1.526065	0.002962	1964
LOC691308	ENSRNOT00000060297	-1.523925	0.00301	1965
Gabrb2	NM_012957	-1.520836	0.003168	1966
Gpr22	NM_001106722	-1.518254	0.002982	1967
Atad3a	NM_001034922	1.517445	0.003093	1968
Etv3	NM_001106450	1.517246	0.003482	1969
S100a6	NM_053485	1.516722	0.003693	1970
Scaper	ENSRNOT00000067848	-1.51668	0.003012	1971
MGC95152	BC083875	-1.516227	0.002829	1972
Insc	NM_001106285	-1.5159	0.002965	1973
Pdrg1	NM_001014762	1.515665	0.003827	1974
Rpusd1	NM_001105774	-1.515325	0.003212	1975
Dpy19l3	NM_001135835	-1.515322	0.00296	1976
Naa38	NM_001106585	-1.512517	0.003578	1977
	ENSRNOT00000051311	-1.512411	0.002993	1978
Kbtbd7	NM_001012045	-1.512305	0.003441	1979
Arl5b	NM_001015031	1.508805	0.003784	1980
Ccbp2	NM_078621	-1.508083	0.002788	1981
Vrk2	NM_001108366	1.507426	0.003986	1982
Gpsm1	ENSRNOT00000025287	1.505825	0.003428	1983
Gabrg1	ENSRNOT00000003240	-1.50541	0.00362	1984
Mamstr	ENSRNOT00000029220	-1.503852	0.00331	1985
Smo	ENSRNOT00000011356	-1.502276	0.003512	1986
Gdap1	NM_001107897	-1.501562	0.003543	1987
Chst12	NM_001037775	-1.501012	0.003413	1988
Mapkapk2	ENSRNOT00000061070	1.500579	0.003639	1989
Nop2	NM_001191785	1.500283	0.003493	1990
Ccl3	NM_013025	4.553214	0.004724	1991
Ccl4	NM_053858	4.439651	0.005568	1992
Sele	ENSRNOT00000030677	4.149317	0.005558	1993
Aloxe3	NM_001105793	3.965387	0.004327	1994
Efcab1	NM_001106930	-3.243258	0.004322	1995
Mcoln3	NM_001012059	2.738649	0.00467	1996
	ENSRNOT00000034474	2.707085	0.004686	1997

Mt1a	ENSRNOT00000057898	2.690421	0.005119	1998
RGD1561381	ENSRNOT00000004798	-2.669734	0.004115	1999
Tmco5a	ENSRNOT00000006794	2.612195	0.004668	2000
Abcb1b	NM_012623	-2.571365	0.004435	2001
Rprm	NM_001044276	2.566522	0.004952	2002
Prg4	NM_001105962	2.55919	0.004556	2003
Slc6a13	NM_133623	-2.546255	0.003877	2004
Thrsp	NM_012703	-2.516611	0.003895	2005
Lgi3	NM_001107277	-2.467097	0.004102	2006
Adamts3	NM_001107212	-2.452979	0.004283	2007
Gjc2	NM_001100784	-2.353326	0.004072	2008
Fn1	NM_019143	2.323156	0.004761	2009
Nmb	NM_001109149	-2.250053	0.00512	2010
	ENSRNOT00000027984	-2.225749	0.004633	2011
Rasgrf2	ENSRNOT00000019087	-2.196821	0.004036	2012
MGC105649	NM_001008518	2.165216	0.00483	2013
Mgp	ENSRNOT00000007577	2.160777	0.00497	2014
Il16	NM_001105749	-2.139441	0.004224	2015
Shc4	NM_001191065	2.121781	0.004576	2016
Zfp238	ENSRNOT00000005849	-2.119365	0.004708	2017
Fah	ENSRNOT00000068167	-2.080885	0.004281	2018
Kcnd2	NM_031730	-2.075573	0.004727	2019
LOC10036260€	XM_002727259	2.063805	0.004648	2020
Gnal4	NM_001013151	-2.047026	0.004081	2021
Bcl11b	NM_001108057	-2.04353	0.004175	2022
Cp	ENSRNOT00000016083	2.038771	0.004613	2023
Pax1	NM_001107787	2.028624	0.00546	2024
Hcrtr1	NM_013064	2.027335	0.004466	2025
Megf11	ENSRNOT00000037941	2.017514	0.004171	2026
Slamf8	NM_001105973	2.01332	0.004225	2027
Epha4	NM_001162411	-2.008056	0.004589	2028
	ENSRNOT00000060666	-2.007586	0.003917	2029
Angptl4	NM_199115	1.990572	0.005408	2030
Vstm2b	NM_001108479	1.981166	0.005172	2031
	ENSRNOT00000060420	1.976085	0.005177	2032
Sult5a1	NM_001201369	-1.968269	0.004993	2033
Ccnd1	NM_171992	1.964523	0.004526	2034
Chml	NM_001109524	1.961835	0.004518	2035
Ppil6	ENSRNOT00000066634	-1.961737	0.003823	2036
Acan	NM_022190	1.959229	0.005373	2037
LOC100361607	XM_002729069	1.958012	0.004463	2038
	ENSRNOT00000066467	1.939648	0.00453	2039
Birc3	NM_023987	1.939057	0.005066	2040
Igfbp4	NM_001004274	-1.925529	0.0041	2041
Rnf112	NM_138613	-1.912227	0.004402	2042
Calcb	ENSRNOT00000014764	1.872593	0.005286	2043
Sntb1	NM_001130542	-1.860786	0.004745	2044
	ENSRNOT00000066667	1.85498	0.004984	2045
Rbm47	NM_001005882	1.854441	0.004203	2046
Bmp2	ENSRNOT00000028904	1.834883	0.005563	2047
	GENSCAN00000036930	-1.833258	0.004533	2048
Pdgfd	NM_023962	-1.822525	0.003963	2049
LOC363320	XM_001075455	-1.809759	0.004454	2050
Slc41a2	NM_001108742	1.809042	0.004471	2051

Cst6	ENSRNOT00000027727	-1.803338	0.004547	2052
Otop3	NM_001105852	-1.797144	0.004979	2053
Higd2al1	ENSRNOT00000001416	-1.794667	0.004501	2054
Cd55	NM_022269	-1.793147	0.004452	2055
Hadh	ENSRNOT00000014658	-1.791162	0.004704	2056
Arl15	ENSRNOT00000014761	-1.78997	0.00441	2057
LOC679566	ENSRNOT00000006921	1.789049	0.004521	2058
	GENSCAN00000002492	-1.784292	0.003948	2059
Ryr2	ENSRNOT00000067949	-1.777809	0.004983	2060
Plk5	NM_001170557	-1.777472	0.004793	2061
RGD1562699	NM_001106141	1.776832	0.004462	2062
Abca9	ENSRNOT00000005775	-1.767086	0.004795	2063
Smpdl3a	NM_001005539	-1.767038	0.003822	2064
Ctrb1	ENSRNOT00000026017	1.766998	0.005672	2065
	ENSRNOT00000037434	-1.752535	0.004365	2066
Cnih3	NM_001166578	-1.748421	0.004522	2067
Scara3	NM_001108870	-1.747756	0.004597	2068
Dclk3	NM_001191800	-1.74655	0.004126	2069
Accn1	NM_001034014	-1.743949	0.003893	2070
Bphl	NM_001037206	-1.73241	0.005254	2071
Fyb	NM_001109176	1.731714	0.005491	2072
Prrt1	NM_001032285	-1.729446	0.004085	2073
Sod3	NM_012880	-1.728451	0.004469	2074
LOC689408	ENSRNOT00000009204	1.723971	0.004303	2075
Rexo4	NM_001033884	1.723747	0.005073	2076
Tnfaip2	NM_001137633	1.719212	0.004241	2077
Lrrc4c	NM_001107753	-1.710364	0.004815	2078
Chrm3	ENSRNOT00000019662	-1.704499	0.0045	2079
Sepp1	NM_019192	-1.701716	0.004538	2080
Cpne4	NM_001109003	-1.701317	0.005227	2081
Zfp385b	NM_001107736	-1.70112	0.004191	2082
Bmp6	NM_013107	-1.693423	0.005245	2083
Tes	NM_001039344	1.69102	0.00502	2084
Fam26e	BC089225	1.684201	0.005318	2085
Ampd3	ENSRNOT00000024933	1.682042	0.005392	2086
Fcgr2a	NM_053843	1.681578	0.004654	2087
Clec2d	ENSRNOT00000010098	1.681557	0.004665	2088
	ENSRNOT00000019301	-1.673435	0.004898	2089
Adamts2	NM_001137622	-1.673389	0.004617	2090
Cthrc1	NM_172333	1.665888	0.005189	2091
Tekt5	ENSRNOT00000003465	-1.664259	0.004631	2092
Ephb1	NM_001104528	-1.661626	0.003951	2093
Flrt3	NM_001126291	-1.661385	0.003996	2094
Gpr12	NM_001037295	-1.657526	0.00528	2095
Ndst3	NM_001191716	-1.657487	0.005074	2096
Slc7a12	NM_001011948	1.651619	0.004595	2097
Grip1	ENSRNOT00000064448	1.650469	0.004678	2098
	GENSCAN00000002492	-1.649512	0.003918	2099
Heph	NM_133304	-1.64911	0.005097	2100
Itgbl1	NM_001017505	-1.648524	0.003814	2101
Usp2	ENSRNOT00000009975	-1.647453	0.004213	2102
	ENSRNOT00000064378	1.64232	0.00438	2103
Tmem126b	ENSRNOT00000038279	-1.640801	0.004244	2104
Cntnap5	NM_001047866	-1.640499	0.004679	2105

Il18	ENSRNOT00000013093	1.639521	0.00416	2106
Arhgap22	NM_001107297	1.637425	0.005164	2107
Myo3b	NM_001191901	1.635707	0.005333	2108
Cntn4	ENSRNOT00000007788	-1.631472	0.004707	2109
Kndc1	ENSRNOT00000034426	-1.629244	0.005122	2110
Sesn1	NM_001106396	-1.627786	0.004806	2111
Olr1616	NM_001000840	1.624315	0.004271	2112
Gpr68	NM_001108049	1.617769	0.004449	2113
Bex1	NM_001037365	-1.6168	0.004208	2114
RGD1563092	NM_001200045	-1.615204	0.004206	2115
Sgcz	NM_001108875	-1.614008	0.004354	2116
	ENSRNOT00000048303	-1.610047	0.004974	2117
Rfx3	NM_001012172	-1.609334	0.003873	2118
Cyth1	NM_053910	-1.608405	0.00406	2119
RGD1306063	BC168755	-1.608107	0.003851	2120
Frem3	NM_001191699	-1.604826	0.004699	2121
	ENSRNOT00000050834	1.601507	0.004598	2122
Nasp	NM_001005543	1.597905	0.004162	2123
Hrh1	ENSRNOT00000009775	1.593126	0.004511	2124
Adcy4	NM_019285	1.592181	0.004343	2125
Parp9	NM_001103351	1.591262	0.004563	2126
Steap1	NM_001106629	1.588109	0.004922	2127
	ENSRNOT00000056493	-1.58776	0.004044	2128
Tslp	ENSRNOT00000065616	1.587191	0.005366	2129
	ENSRNOT00000011178	-1.585843	0.003915	2130
Myo19	NM_001163736	1.58268	0.004259	2131
Gng7	NM_024138	-1.58094	0.005213	2132
Fam5c	NM_173121	-1.579653	0.004143	2133
MGC116197	BC098041	-1.578764	0.004933	2134
Atp2b3	ENSRNOT00000023934	-1.578726	0.004086	2135
	ENSRNOT00000046404	-1.578416	0.004429	2136
Masp1	ENSRNOT00000067223	-1.577678	0.003985	2137
Clcn5	NM_017106	1.576078	0.004377	2138
Olr1227	NM_001000443	-1.575582	0.004423	2139
Lgi4	ENSRNOT00000028632	-1.574834	0.005051	2140
Ptchd1	NM_001191734	1.574058	0.005263	2141
LOC690286	ENSRNOT00000055664	-1.572639	0.004136	2142
	ENSRNOT00000059264	-1.571076	0.005069	2143
B3galt5	NM_001105887	-1.570349	0.004196	2144
	GENSCAN00000009960	-1.568377	0.004146	2145
	ENSRNOT00000027019	-1.568129	0.004468	2146
Gabrg2	ENSRNOT00000004619	-1.56628	0.003964	2147
Cdk18	NM_001100506	-1.565329	0.003978	2148
Fcho1	NM_001106069	-1.563622	0.003938	2149
Nkiras1	NM_001107252	-1.563159	0.004362	2150
Slc27a1	NM_053580	-1.562676	0.004236	2151
Fam81a	NM_001108163	-1.562398	0.005031	2152
Manba	NM_001031655	-1.562161	0.004456	2153
Foxo6	XM_001053458	-1.561955	0.003973	2154
Avp	NM_016992	1.558957	0.00477	2155
Sort1	NM_031767	-1.558206	0.004683	2156
Fam70b	ENSRNOT00000030100	-1.555632	0.004515	2157
Abcc8	ENSRNOT00000028696	-1.553088	0.004066	2158
Trem3	NM_001191581	1.549675	0.004252	2159

Ldlr	NM_175762	1.548826	0.004735	2160
Klf15	NM_053536	-1.548776	0.004568	2161
Hsd17b11	NM_001004209	-1.54819	0.003888	2162
Uba7	NM_001106856	-1.547866	0.004111	2163
Ccnl1	NM_053662	1.547143	0.004965	2164
Il17rd	NM_001191937	-1.545885	0.004499	2165
Lmo2	NM_001037358	1.542512	0.005595	2166
	ENSRNOT00000008101	-1.540661	0.004954	2167
LOC100364194	XM_002724633	1.539295	0.004574	2168
	ENSRNOT00000012825	1.53815	0.00436	2169
Chrna7	ENSRNOT00000051883	-1.537748	0.004605	2170
Tmod1	NM_013044	1.536887	0.005527	2171
Riok2	NM_001009687	1.535468	0.00426	2172
Cntnap5a	NM_001047865	-1.534338	0.004908	2173
Tmem119	NM_001107155	-1.533692	0.005167	2174
Osr1	NM_001106716	-1.528822	0.005151	2175
Porcn	NM_001173355	-1.528363	0.005182	2176
Spock3	NM_001107310	-1.527237	0.004189	2177
	ENSRNOT00000029858	-1.526631	0.004524	2178
Hck	NM_013185	1.52659	0.004578	2179
Ppp1r1a	NM_022676	1.525412	0.004987	2180
Ccl19	NM_001108661	-1.523851	0.004099	2181
11-Mar	NM_001101828	1.523612	0.004638	2182
Folr4	ENSRNOT00000051471	1.523017	0.005283	2183
Dgat2	NM_001012345	-1.522098	0.004195	2184
Rspo3	ENSRNOT00000015395	-1.521191	0.004477	2185
Cacna1c	NM_012517	-1.518256	0.004237	2186
Eml5	AY445136	1.517095	0.005277	2187
Abca2	ENSRNOT00000020339	-1.516573	0.004299	2188
Atp2b2	NM_012508	-1.515771	0.00421	2189
Cdh13	NM_138889	-1.513724	0.003828	2190
Ctsh	ENSRNOT00000019285	-1.512515	0.004285	2191
	ENSRNOT00000067950	1.512156	0.004246	2192
Nipal3	ENSRNOT00000068004	-1.511797	0.003912	2193
Cyp4f6	NM_153318	-1.508921	0.004911	2194
Epb4114b	ENSRNOT00000015114	1.50854	0.005308	2195
Kcnk6	ENSRNOT00000027974	1.508159	0.0056	2196
RGD1310039	NM_001014165	-1.508144	0.004033	2197
Olr894	NM_001000940	1.507146	0.005644	2198
Epb4.1l4b	XM_345535	-1.50604	0.004404	2199
Ly96	NM_001024279	1.505863	0.004528	2200
Accn2	NM_024154	-1.505556	0.003936	2201
Irak3	ENSRNOT00000005686	1.504616	0.005589	2202
Actn2	NM_001170325	-1.50454	0.004719	2203
Prkx	NM_001033963	1.504298	0.004839	2204
LOC10036271C	ENSRNOT00000022567	-1.504183	0.004772	2205
Tnfaip8	NM_001107387	-1.504088	0.004173	2206
Elovl7	ENSRNOT00000014074	-1.503558	0.003881	2207
RGD1564664	NM_001110055	1.502069	0.004744	2208
Erf	NM_001170335	1.501754	0.004778	2209
RGD1560166	XM_576464	-1.501082	0.003921	2210
Tnn	NM_001107189	1.500526	0.005209	2211
Zfp458	NM_001024299	-1.500011	0.004214	2212
Hspa1b	NM_212504	3.678476	0.00577	2213

Npas4	NM_153626	3.436555	0.007343	2214
Sult1a1	NM_031834	-3.116341	0.006592	2215
Nppa	NM_012612	2.895737	0.008509	2216
Slc19a3	NM_001108228	-2.768222	0.006224	2217
Cdkn3	NM_001106028	2.68472	0.006047	2218
Cplx4	NM_001191835	2.463227	0.006535	2219
Lamc2	NM_001100640	2.438763	0.008024	2220
Mog	NM_022668	-2.335159	0.007542	2221
Gpd1	NM_022215	2.325168	0.007574	2222
Postn	NM_001108550	2.14285	0.006378	2223
Slc13a4	ENSRNOT00000059892	-2.137077	0.007811	2224
Hpse	NM_022605	2.110831	0.006775	2225
Clec4a1	NM_001005890	2.10029	0.006947	2226
Slc30a3	NM_001013243	-2.078128	0.007847	2227
Mt2A	ENSRNOT00000067391	2.069925	0.007784	2228
LOC680069	ENSRNOT00000039210	2.06125	0.008119	2229
RGD1310819	ENSRNOT00000068496	-2.027152	0.00758	2230
	GENSCAN00000011876	-2.017712	0.005642	2231
Lypd1	NM_001007727	-2.015049	0.005922	2232
	ENSRNOT00000058914	-2.009972	0.005883	2233
Frzb	NM_001100527	-1.992672	0.007251	2234
Kcnj10	ENSRNOT00000010146	-1.981255	0.005583	2235
Cldn1	ENSRNOT00000002640	-1.964925	0.005979	2236
Enpp6	NM_001107311	-1.963409	0.007837	2237
Dusp1	NM_053769	1.937463	0.005763	2238
Ace	NM_012544	1.935915	0.006452	2239
Cxcl10	ENSRNOT00000003075	1.907726	0.006996	2240
	ENSRNOT00000060889	-1.898191	0.006046	2241
Cyp4f1	NM_019623	-1.896019	0.005374	2242
Stc2	NM_022230	1.895412	0.008409	2243
Prc1	NM_001107529	1.889688	0.006007	2244
	ENSRNOT00000017696	1.866476	0.006744	2245
Lcp1	NM_001012044	1.84838	0.00713	2246
	ENSRNOT00000005162	-1.837559	0.007804	2247
Ms4a4a	XM_342027	1.832431	0.007562	2248
Pou4f3	NM_001108889	1.829895	0.008781	2249
LOC100363141	XM_003754442	1.827784	0.006737	2250
Psmb8	NM_080767	1.825674	0.005819	2251
Siah3	NM_001191118	1.816534	0.008032	2252
Grem2	NM_001105974	1.814861	0.006274	2253
Arc	ENSRNOT00000067442	1.810708	0.006715	2254
Dpf3	ENSRNOT00000010926	-1.80885	0.006756	2255
	ENSRNOT00000055774	-1.802878	0.00578	2256
Rab26	ENSRNOT00000004249	-1.770886	0.006847	2257
Calb2	ENSRNOT00000022943	-1.767385	0.005352	2258
Th	NM_012740	1.767221	0.00732	2259
Grm2	NM_001105711	-1.764228	0.006165	2260
Hpca	ENSRNOT00000009153	-1.745867	0.007787	2261
Pnlip	NM_013161	-1.719594	0.005586	2262
Irf7	NM_001033691	-1.716802	0.006136	2263
Rspo2	NM_001130575	-1.709198	0.005711	2264
Gpc3	NM_012774	-1.706955	0.007214	2265
Thbd	NM_031771	1.705801	0.00587	2266
Nr3c2	ENSRNOT00000052018	-1.705334	0.005669	2267

Dusp19	NM_001107739	-1.70018	0.005775	2268
LOC501223	XM_001071000	-1.692726	0.005862	2269
	ENSRNOT00000039048	1.687226	0.008309	2270
Pllp	NM_022533	-1.686449	0.005726	2271
Ms4a6b	NM_001006975	1.683979	0.00747	2272
Mobp	ENSRNOT00000025343	-1.683425	0.007316	2273
	ENSRNOT00000048419	1.679935	0.007108	2274
Kcnj11	NM_031358	-1.674439	0.00548	2275
Nt5e	ENSRNOT00000015057	1.673014	0.006614	2276
Eepd1	NM_001014088	-1.672115	0.006591	2277
Pitpnm2	NM_001107139	-1.670396	0.00558	2278
	ENSRNOT00000019667	1.668764	0.007948	2279
Ppp3ca	ENSRNOT00000013305	-1.666945	0.005556	2280
Zbtb20	NM_001105880	-1.663994	0.005947	2281
Ccdc111	ENSRNOT00000066653	1.662136	0.006502	2282
Mid1	ENSRNOT00000004873	-1.655606	0.005424	2283
Satb1	NM_001012129	-1.651613	0.005427	2284
LOC690467	XM_003751857	-1.650093	0.007853	2285
Galnt13	NM_199106	-1.644759	0.005513	2286
Kcnv1	NM_021697	-1.643252	0.006379	2287
Limd2	NM_001025715	-1.642173	0.005918	2288
	ENSRNOT00000057904	-1.637979	0.005458	2289
Hrh3	NM_053506	-1.635981	0.006006	2290
RGD1560088	XM_574423	-1.63385	0.006483	2291
Zdbf2	ENSRNOT00000016038	1.632068	0.006301	2292
Rps6ka6	NM_001191721	1.631355	0.005719	2293
F7	NM_152846	1.628193	0.007599	2294
Lama2	ENSRNOT00000014917	-1.625669	0.007809	2295
Ifi27	NM_203410	-1.621209	0.005546	2296
Nuak2	NM_001007617	1.619004	0.007756	2297
Magee1	NM_001079891	-1.608917	0.00581	2298
C1s	ENSRNOT00000016330	1.60726	0.006343	2299
LOC685619	XM_003753830	1.604845	0.006989	2300
Cd34	NM_001107202	-1.602074	0.005802	2301
Grasp	NM_138894	1.601802	0.006184	2302
Pcdh20	NM_001107280	-1.60057	0.006795	2303
Lgals9	NM_012977	-1.600228	0.00571	2304
Ctgf	NM_022266	1.600073	0.006734	2305
	ENSRNOT00000057187	-1.599247	0.006213	2306
Cd163	NM_001107887	1.596234	0.007646	2307
Mrgprf	NM_153722	1.5957	0.005741	2308
	ENSRNOT00000034750	1.595327	0.007106	2309
Grap2	NM_001034944	-1.592589	0.005389	2310
Rasd2	ENSRNOT00000020017	-1.57991	0.006869	2311
Ccdc109b	ENSRNOT00000012587	1.577515	0.00761	2312
Lrrtm4	NM_001134746	-1.576708	0.005953	2313
Hist1h2ac	XM_003751712	1.576521	0.00679	2314
Mms22l	NM_001135780	1.574853	0.005998	2315
Rab38	NM_145774	-1.574765	0.005777	2316
Kcnn4	NM_023021	1.574316	0.007818	2317
Lrrc69	NM_001134624	1.56905	0.008778	2318
Ankrd45	ENSRNOT00000003857	-1.568082	0.006021	2319
Fkbp5	NM_001012174	1.567709	0.005928	2320
Tecta	NM_001106814	-1.566158	0.006161	2321

Chst10	ENSRNOT00000017714	1.565455	0.007128	2322
Cgref1	NM_139087	1.564845	0.007148	2323
Hebp2	NM_001107515	-1.560205	0.005812	2324
	ENSRNOT00000043510	-1.55753	0.005962	2325
Rragd	ENSRNOT00000068028	-1.555256	0.007851	2326
	ENSRNOT00000061062	-1.552341	0.007591	2327
	ENSRNOT00000048551	-1.551668	0.007053	2328
Cyp4f17	ENSRNOT00000060284	-1.551229	0.006859	2329
Pygl	ENSRNOT00000009183	1.549544	0.006749	2330
	ENSRNOT00000061338	-1.547061	0.005419	2331
Filip1	NM_145682	-1.544701	0.006299	2332
Cldn5	NM_031701	-1.543893	0.006991	2333
Apoc1	NM_012824	-1.54274	0.006816	2334
RGD1564688	ENSRNOT00000058917	-1.542227	0.005312	2335
Rasl11a	NM_001002829	1.540725	0.005839	2336
Cnih2	NM_001025132	-1.539859	0.006604	2337
Clec4d	ENSRNOT00000013536	1.539776	0.008322	2338
Cdh22	NM_019161	1.537692	0.00839	2339
	GENSCAN00000008206	-1.535901	0.005433	2340
LOC100362279	XM_003753476	1.535514	0.007945	2341
LOC292722	XM_002728703	-1.534288	0.005882	2342
Bub1	NM_001106507	1.532682	0.007495	2343
Cntnap5b	NM_001047873	-1.531595	0.006534	2344
LOC100360856	XM_002730136	-1.528142	0.00772	2345
Pcdhb9	NM_001109390	-1.524496	0.006061	2346
	ENSRNOT00000012194	-1.523063	0.005406	2347
Dnai1	NM_001024342	-1.52298	0.005413	2348
Naaladl2	XM_001062720	-1.522772	0.005497	2349
Mef2c	ENSRNOT00000041296	-1.521969	0.005446	2350
Fndc3a	NM_001107278	1.521739	0.006471	2351
LOC100364306	ENSRNOT00000041850	-1.521058	0.007871	2352
Pyy	ENSRNOT00000028323	1.518224	0.008099	2353
Plekhg1	NM_001190999	-1.51763	0.005721	2354
	ENSRNOT00000040063	-1.516558	0.007156	2355
Gprasp1	NM_134386	-1.516505	0.005731	2356
Alox12b	NM_001039377	-1.514147	0.005543	2357
RGD1560691	NM_001107365	-1.513932	0.007021	2358
LOC100366268	ENSRNOT00000060139	1.513699	0.00677	2359
St18	ENSRNOT00000059625	-1.513443	0.005863	2360
Dusp4	NM_022199	1.511704	0.007782	2361
Nid67	NM_173126	1.510901	0.007998	2362
	ENSRNOT00000019453	-1.506176	0.006295	2363
Cwc25	NM_001108295	1.504533	0.006732	2364
Pdgfb	NM_031524	1.501767	0.006432	2365
	ENSRNOT00000064055	3.79589	0.011071	2366
Plek2	NM_001114180	3.194657	0.009034	2367
Car3	ENSRNOT00000014180	-2.968559	0.012516	2368
Gnas	NM_001159653	2.556789	0.012104	2369
	ENSRNOT00000036735	-2.539649	0.013943	2370
Slpi	NM_053372	2.474823	0.013692	2371
Tpbg	ENSRNOT00000014326	2.456677	0.009002	2372
Pcdhb6	NM_001014780	-2.358383	0.011692	2373
LOC24906	NM_031537	2.344616	0.010726	2374
Ptger3	NM_012704	2.293697	0.010165	2375

Nts	NM_001102381	-2.255211	0.009221	2376
Mmp12	NM_053963	2.223494	0.013459	2377
Clec2dl1	NM_001085404	2.194352	0.010469	2378
Fam163b	NM_001109458	-2.161436	0.009568	2379
Omd	ENSRNOT00000020648	-2.092646	0.008853	2380
Car7	NM_001106165	-2.085039	0.012677	2381
Slc39a12	ENSRNOT00000044313	2.062999	0.008975	2382
RGD1559662	XM_001059769	2.023994	0.011603	2383
Klhl14	ENSRNOT00000020697	-2.014461	0.011938	2384
Dnajb13	NM_001005885	-2.008888	0.010457	2385
Cadm2	NM_001047102	-1.956713	0.009122	2386
Hapln4	NM_001108398	-1.948866	0.010828	2387
Dio2	NM_031720	-1.92857	0.007944	2388
Aspn	ENSRNOT00000020704	-1.926763	0.00968	2389
Plac9	NM_001108395	-1.905981	0.011557	2390
Tspan9	ENSRNOT00000007328	1.868661	0.011403	2391
LOC100364994	ENSRNOT00000064931	-1.866815	0.008114	2392
Hpse	NM_022605	1.850448	0.008934	2393
Fras1	NM_001191595	1.840726	0.009825	2394
Vom2r37	ENSRNOT00000056515	1.812837	0.009406	2395
	ENSRNOT00000058894	1.805475	0.012959	2396
Pde7b	NM_080894	-1.795727	0.010102	2397
Crabp2	NM_017244	1.784377	0.010009	2398
Fkbp9	NM_001007646	-1.780881	0.008553	2399
Tmem144	NM_001108551	-1.778194	0.0083	2400
Mgll	NM_138502	-1.769896	0.00987	2401
Kcnn3	NM_019315	1.763151	0.016175	2402
Retsat	ENSRNOT00000019571	-1.761464	0.012631	2403
Pbld	NM_138530	-1.756244	0.009148	2404
Slc17a6	ENSRNOT00000022383	1.746699	0.015278	2405
Wipf3	NM_147211	-1.744251	0.009966	2406
Prex2	ENSRNOT00000064774	-1.738098	0.009535	2407
RT1-Da	NM_001008847	1.734802	0.012373	2408
	GENSCAN00000022774	-1.730247	0.012081	2409
Ltbp1	ENSRNOT00000040931	1.728705	0.011187	2410
Dkk3	NM_138519	-1.721553	0.012058	2411
	ENSRNOT00000012503	-1.716024	0.008111	2412
Cxcl11	ENSRNOT00000031667	1.71455	0.015124	2413
Ppp1r14a	NM_130403	-1.712329	0.011291	2414
Ptgfr	NM_013115	1.700665	0.010207	2415
Plk4	NM_001107669	1.69538	0.010006	2416
Gpr165	NM_001106582	-1.695026	0.013182	2417
Crhr1	NM_030999	1.690135	0.01563	2418
Loxl4	NM_001107592	1.689228	0.013931	2419
Ifit1	NM_020096	-1.687144	0.010658	2420
Cpm	NM_001108098	-1.68032	0.009492	2421
Mei1	NM_001130555	-1.679622	0.010295	2422
Serpinf1	NM_177927	1.67916	0.010354	2423
Cacng3	NM_080691	-1.678862	0.009468	2424
Mdk	NM_030859	-1.674435	0.010154	2425
Cdh23	ENSRNOT00000048029	1.666209	0.010915	2426
Samsn1	ENSRNOT00000040202	1.662097	0.013278	2427
Ptges	ENSRNOT00000045993	1.656038	0.009368	2428
Prox1	NM_001107201	-1.649135	0.012352	2429

St5	NM_001107547	-1.648844	0.008389	2430
LOC685596	ENSRNOT00000046035	-1.64659	0.010706	2431
Slc22a6	ENSRNOT00000024757	-1.645972	0.010595	2432
LOC690467	XM_003751857	-1.635259	0.00801	2433
Uqcrb	NM_001127553	-1.634259	0.007951	2434
Lrrn1	NM_001037363	-1.632491	0.010514	2435
	ENSRNOT00000015993	1.630363	0.009834	2436
Scn7a	NM_031686	1.62047	0.012708	2437
LOC10036430€	ENSRNOT00000041850	-1.620278	0.012842	2438
Nptx1	NM_153735	-1.617881	0.012562	2439
Il17rb	NM_001107290	1.615735	0.0115	2440
LOC10036430€	ENSRNOT00000041850	-1.614707	0.009271	2441
LOC685699	XM_001064894	-1.613221	0.011846	2442
Cdh7	ENSRNOT00000041413	-1.607392	0.009622	2443
Wnt9b	NM_001107055	1.606378	0.009171	2444
Dsg1b	ENSRNOT00000061344	1.60019	0.015922	2445
RGD1564019	ENSRNOT00000032897	-1.598638	0.009708	2446
Tfcp2l1	NM_001107170	-1.595931	0.012875	2447
Rel	ENSRNOT00000065110	1.594821	0.01116	2448
RGD1562650	ENSRNOT00000068178	1.593996	0.010073	2449
Anxa4	NM_024155	1.593364	0.011735	2450
Gsg1l	ENSRNOT00000022392	1.582127	0.013091	2451
Galnt6	NM_001172063	1.580384	0.010436	2452
Klf5	NM_053394	1.580303	0.014427	2453
Clmp	NM_173154	-1.580251	0.013189	2454
Stxbp6	NM_001191872	-1.579263	0.011379	2455
Npsr1	NM_001106808	-1.577974	0.011925	2456
Syt7	NM_021659	-1.575384	0.008878	2457
Dhrs9	ENSRNOT00000008921	1.57227	0.009874	2458
Akr7a3	NM_013215	-1.56835	0.012324	2459
Sertad4	NM_001108351	-1.56802	0.009533	2460
	ENSRNOT00000057536	-1.566629	0.013778	2461
RGD1563499	ENSRNOT00000060372	1.563866	0.015551	2462
RGD1561955	ENSRNOT00000044063	-1.560046	0.013959	2463
Hs3st2	ENSRNOT00000023773	1.559062	0.015967	2464
RGD1566006	XM_577240	1.556435	0.016181	2465
Sptb	ENSRNOT00000009028	-1.545585	0.009163	2466
Dpysl3	NM_012934	1.540859	0.011391	2467
RGD1560672	NM_001109073	-1.540678	0.010859	2468
	ENSRNOT00000041121	1.540076	0.014784	2469
Efhc1	NM_001122947	-1.53907	0.011549	2470
Syndig1	NM_001025020	-1.538898	0.008165	2471
	ENSRNOT00000045820	1.536359	0.01103	2472
Mal1	NM_001014182	1.53604	0.013974	2473
RGD1565690	ENSRNOT00000039316	-1.535898	0.009699	2474
Coch	ENSRNOT00000007365	-1.532556	0.009383	2475
LOC10036430€	ENSRNOT00000041850	-1.530007	0.013513	2476
Cxcl5	ENSRNOT00000003823	1.529837	0.010629	2477
Igsf1	NM_175763	-1.526411	0.008872	2478
Nrip3	NM_001108498	-1.522815	0.013673	2479
Oas1k	NM_001009489	1.518806	0.01165	2480
Retnlg	ENSRNOT00000002673	1.516089	0.01316	2481
Lppr1	ENSRNOT00000009987	-1.514709	0.008601	2482
Tceb1	NM_022593	-1.514	0.009109	2483

Hmgcs2	NM_173094	-1.511877	0.008653	2484
	ENSRNOT00000042839	-1.509663	0.008676	2485
	ENSRNOT00000051710	-1.506404	0.008575	2486
	ENSRNOT00000040676	1.504936	0.009414	2487
	ENSRNOT00000043332	-1.503886	0.011148	2488
Egr3	ENSRNOT00000024067	1.503832	0.015222	2489
LOC10036430€	ENSRNOT00000041850	-1.501958	0.013044	2490
Slit2	NM_022632	-1.500806	0.010652	2491
Ttr	NM_012681	-5.173733	0.021212	2492
RT1-M6-2	NM_001008853	-2.426004	0.01843	2493
Clec4a2	ENSRNOT00000051525	2.243001	0.016631	2494
Kcnj13	NM_053608	-2.211462	0.027183	2495
Mas1	NM_012757	-2.174416	0.020649	2496
Npy2r	NM_023968	2.133234	0.023363	2497
Lilrb4	NM_001013894	2.121848	0.02854	2498
Gabrq	NM_031733	2.116983	0.020959	2499
Npy2r	NM_023968	2.104044	0.02184	2500
Gipr	NM_012714	2.098253	0.018324	2501
Dio3	NM_017210	2.08843	0.024844	2502
Mmp3	NM_133523	2.083542	0.032558	2503
Egr4	NM_019137	2.045069	0.028509	2504
Usp18	NM_001014058	-2.012849	0.018349	2505
Rerg	ENSRNOT00000030850	-1.953865	0.019887	2506
Mmp8	NM_022221	1.937529	0.019373	2507
RGD1563891	ENSRNOT00000052189	-1.933336	0.028037	2508
Slc4a11	NM_001107775	1.877018	0.017787	2509
Olr1462	NM_001001097	-1.85253	0.022701	2510
Pde1c	NM_031078	1.820067	0.016863	2511
Rasl10a	NM_001108862	-1.797871	0.022574	2512
Tas2r143	NM_001025061	1.75427	0.020858	2513
	ENSRNOT00000068215	-1.749086	0.01933	2514
	ENSRNOT00000038579	-1.740778	0.025284	2515
	ENSRNOT00000068390	-1.740668	0.01749	2516
Stoml3	NM_001106431	-1.73042	0.021104	2517
Igf2	NM_001190162	-1.728635	0.01465	2518
	ENSRNOT00000037433	1.708395	0.026155	2519
Satb2	NM_001109306	-1.690794	0.02259	2520
Hsf2bp	NM_001127683	1.673688	0.017728	2521
Fibcd1	NM_001107829	-1.65783	0.021796	2522
Kcnj6	NM_013192	-1.652707	0.020942	2523
Rbp4	NM_013162	-1.648114	0.021673	2524
	ENSRNOT00000056902	-1.636029	0.018436	2525
	ENSRNOT00000048288	-1.633202	0.015018	2526
Vip	NM_053991	1.621161	0.018552	2527
	ENSRNOT00000064176	-1.614705	0.022218	2528
Uox	ENSRNOT00000021970	1.614656	0.017891	2529
Ntf3	NM_031073	-1.610661	0.017153	2530
Cyp2j4	NM_023025	-1.607775	0.019015	2531
Sfrp2	NM_001100700	-1.607253	0.015146	2532
B3gnt5	NM_053932	1.60562	0.025784	2533
Sgk1	NM_019232	1.603162	0.024119	2534
Fmo5	NM_144739	1.600976	0.019994	2535
Zdhhc22	NM_001039325	1.599348	0.026622	2536
Rarres2	NM_001013427	-1.586968	0.022552	2537

Cacna1h	NM_153814	-1.585813	0.017247	2538
Lhx9	ENSRNOT00000013873	-1.583596	0.019955	2539
LOC10036327€	ENSRNOT00000040311	1.580831	0.022136	2540
Caln1	NM_001077201	-1.579413	0.027732	2541
Etl4	ENSRNOT00000057655	-1.575001	0.027845	2542
Sell	ENSRNOT00000003733	1.573774	0.017884	2543
	ENSRNOT00000031662	-1.572721	0.014297	2544
Serpinb1a	NM_001031642	1.56789	0.017201	2545
LOC10036430€	ENSRNOT00000041850	-1.562022	0.014391	2546
Pcdhb4	NM_001114601	-1.560011	0.019823	2547
RGD1564677	XM_002728557	1.552946	0.020756	2548
Lyzl4	HM125534	-1.550878	0.020404	2549
Spata18	NM_199374	-1.547543	0.02255	2550
C1ql3	ENSRNOT00000023637	-1.545169	0.016502	2551
Anpep	NM_031012	1.543679	0.026037	2552
LOC10036430€	ENSRNOT00000041850	-1.539543	0.017715	2553
LOC10036430€	ENSRNOT00000041850	-1.539543	0.017715	2554
	ENSRNOT00000052377	-1.538936	0.023259	2555
Ddit4l	NM_080399	-1.534921	0.021947	2556
LOC10036430€	ENSRNOT00000041850	-1.534253	0.016441	2557
Dgkz	ENSRNOT00000024280	-1.533936	0.022124	2558
Chrm4	NM_031547	-1.532885	0.018615	2559
	ENSRNOT00000040047	-1.532529	0.020585	2560
Dusp14	BC158555	1.528453	0.023994	2561
	ENSRNOT00000028498	1.527311	0.016681	2562
RGD1562220	ENSRNOT00000003207	1.526794	0.030703	2563
RGD1566007	XM_003754559	1.524375	0.020578	2564
Klhl4	NM_001108244	-1.520132	0.020993	2565
Pbx3	NM_001107834	1.517757	0.024738	2566
C1qtnf5	NM_001012123	-1.51717	0.023415	2567
LOC690467	XM_003751857	-1.516566	0.019813	2568
	ENSRNOT00000041483	-1.516009	0.016906	2569
	ENSRNOT00000041800	-1.51242	0.01459	2570
LOC685320	ENSRNOT00000064675	1.511831	0.018695	2571
Kcnh3	ENSRNOT00000045515	-1.50992	0.01446	2572
	ENSRNOT00000068439	1.509027	0.017785	2573
Sh3bp5	NM_054011	-1.506105	0.021228	2574
Kcnf1	NM_001169104	2.021955	0.052881	2575
Lbp	ENSRNOT00000019787	1.822086	0.049573	2576
Drd5	NM_012768	1.772643	0.061461	2577
Kngl1l	NM_001009628	1.763109	0.048393	2578
Selp	ENSRNOT00000003759	1.76061	0.034456	2579
Plagl1	NM_012760	1.715426	0.037422	2580
RT1-M6-1	NM_001008852	-1.71381	0.043658	2581
Capsl	NM_001106417	-1.710654	0.051643	2582
Pgr15l	NM_001106583	1.709553	0.041545	2583
Slc38a4	ENSRNOT00000009187	1.708739	0.035226	2584
Hdc	NM_017016	-1.708433	0.032112	2585
Aspa	ENSRNOT00000026743	-1.681311	0.054802	2586
Plch1	NM_001191707	1.664521	0.053301	2587
Epha7	ENSRNOT00000009899	-1.660942	0.041623	2588
Rpl30	K02932	1.655164	0.053392	2589
Fcnb	ENSRNOT00000012494	1.633457	0.036204	2590
Dpt	ENSRNOT00000066119	-1.604734	0.035277	2591

	GENSCAN00000042074	-1.603001	0.032382	2592
Serping1	ENSRNOT00000009817	1.587583	0.048452	2593
Crispld1	NM_001134963	-1.578309	0.040436	2594
	ENSRNOT00000044428	1.5754	0.057149	2595
Necab3	NM_001098724	-1.571523	0.036615	2596
Cftr	NM_031506	-1.564338	0.041375	2597
LOC10036159€	XM_003753961	-1.562059	0.030622	2598
	ENSRNOT00000065440	1.557299	0.057121	2599
Bmp4	NM_012827	-1.553042	0.042225	2600
Klk6	NM_019175	1.546219	0.055166	2601
Cldn11	NM_053457	-1.53598	0.032016	2602
Cbln2	NM_001012740	1.535773	0.053799	2603
Camk2d	NM_012519	1.533328	0.037294	2604
Apln	NM_031612	1.532253	0.051793	2605
Trat1	NM_001037981	1.527843	0.050636	2606
Lrrc34	NM_001044696	-1.527217	0.032561	2607
	ENSRNOT00000056240	-1.519285	0.050119	2608
	ENSRNOT00000042570	-1.515996	0.031016	2609
Tox	NM_001108654	-1.5142	0.047506	2610
Sox18	NM_001024781	1.506774	0.046132	2611
Bbox1	ENSRNOT00000006197	-1.500734	0.046091	2612
