# Supplementary material for: Long non-coding RNA H19 contributes to apoptosis of hippocampal neurons by inhibiting let-7b in a rat model of temporal lobe epilepsy
Source: Cell Death Dis. 2018 May 23;9(6):617. doi: 10.1038/s41419-018-0496-y (PMC5966382; doi:10.1038/s41419-018-0496-y)
Supplement: Supplementary file 3 — Supplementary Figures [file 41419_2018_496_MOESM3_ESM.docx]

**Supplementary Figure 1**


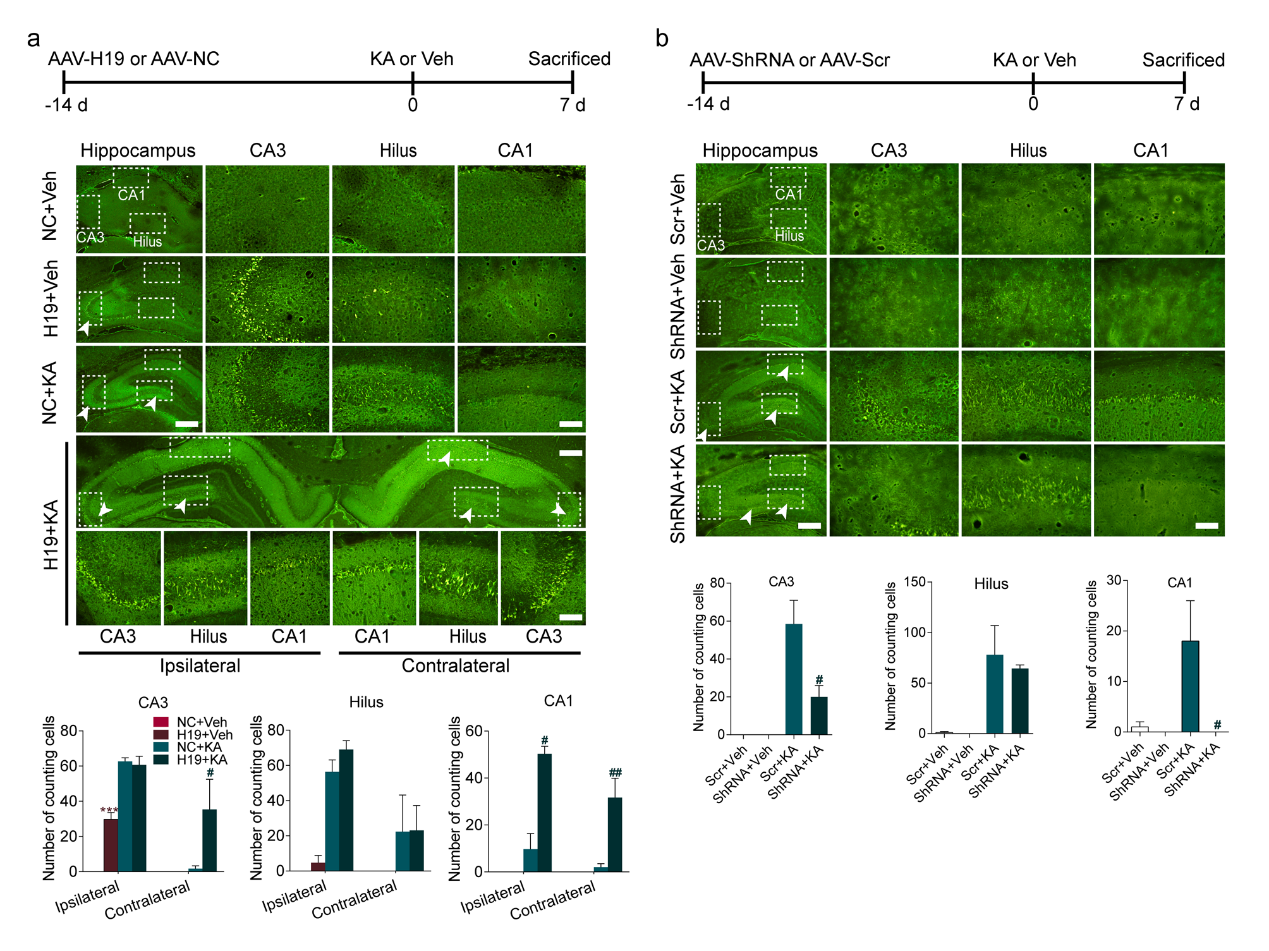


**Supplementary Figure 1** H19 exacerbated SE-induced hippocampal neuronal death *in vivo*. (**a, b**) Top, the timeline showing the experimental design. Middle, representative Fluoro-Jade C (FJC) staining photomicrographs of hippocampus ipsilateral or contralateral to the AAV vectors and/or KA injection side from (**a**) H19 overexpression and (**b**) H19 knockdown rats with or without KA treatment for 7 d. The arrowheads indicate FJC positive neurons (Scale bar = 400 μm). The magnified images correspond to the labeled box from their upper panels (Scale bar = 100 μm). The bottom bar graphs show the count of cells in the CA3, dentate hilus and CA1 regions of hippocampus ipsilateral or contralateral to the KA injection side (n = 4-5). All data are shown as mean ± s.e.m. **p* < 0.05, ** *p* < 0.01, *** *p* < 0.001 versus NC+Veh or Scr+Veh group. # *p* < 0.05, ## *p* < 0.01, ### *p* < 0.001 versus NC+KA or Scr+KA group.


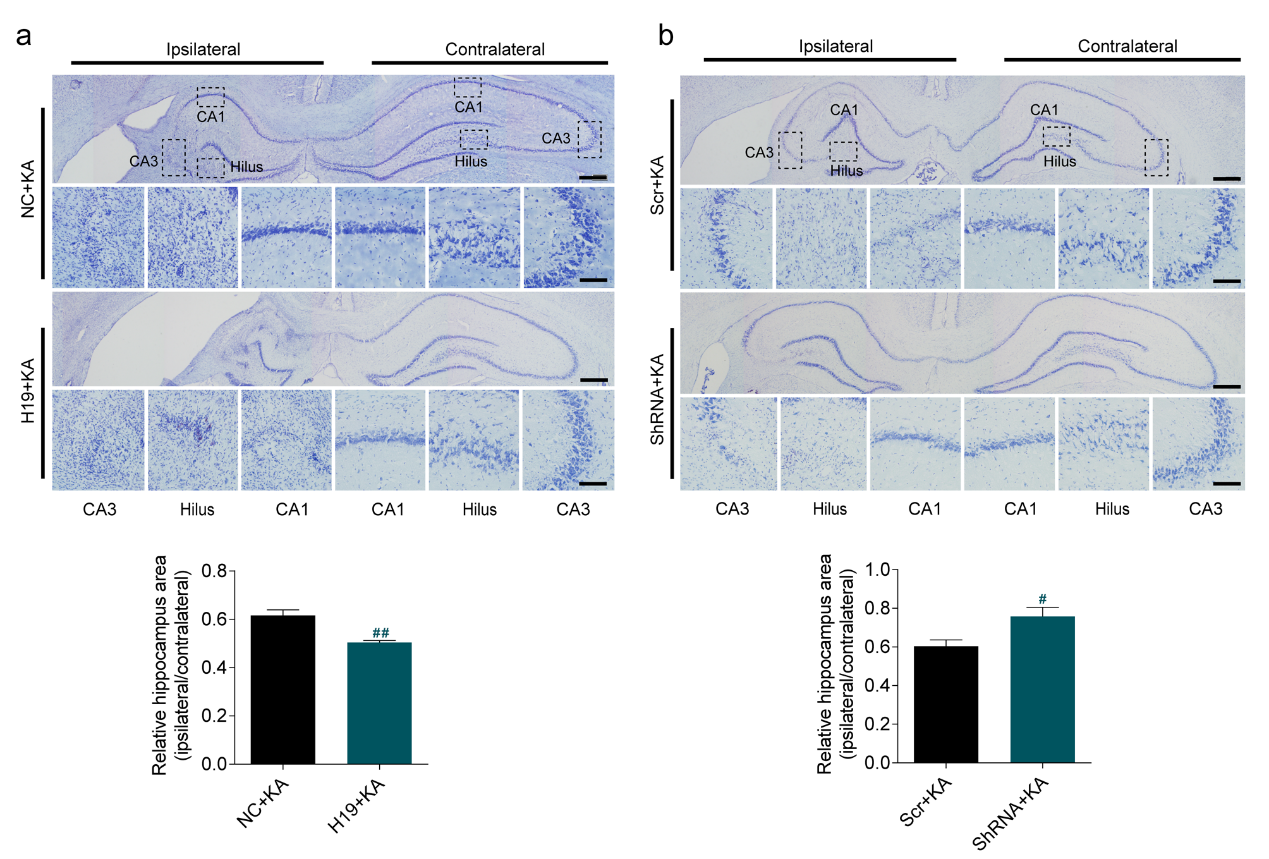


**Supplementary Figure 2** H19 exacerbated SE-induced hippocampal neuronal death *in vivo*. (**a, b**) Top, representative Nissl staining photomicrographs of the hippocampus ipsilateral or contralateral to the AAV vectors and KA injection side from (**a**) H19 overexpression and (**b**) H19 knockdown rats and their corresponding controls (NC+KA or Scr+KA) at 60 d after SE (Scale bar = 400 μm). The magnified images correspond to the labeled box from their upper panels (Scale bar = 100 μm). The bottom panels are the relative ratios of the hippocampal cross-sectional area (ipsilateral area /contralateral area) in H19 overexpression or H19 knockdown rats and their corresponding controls (n = 4-5). All data are shown as mean ± s.e.m. #*p* < 0.05, ##*p* < 0.01, ###*p* < 0.001 versus NC+KA or Scr+KA group.

**Supplementary Figure 3**


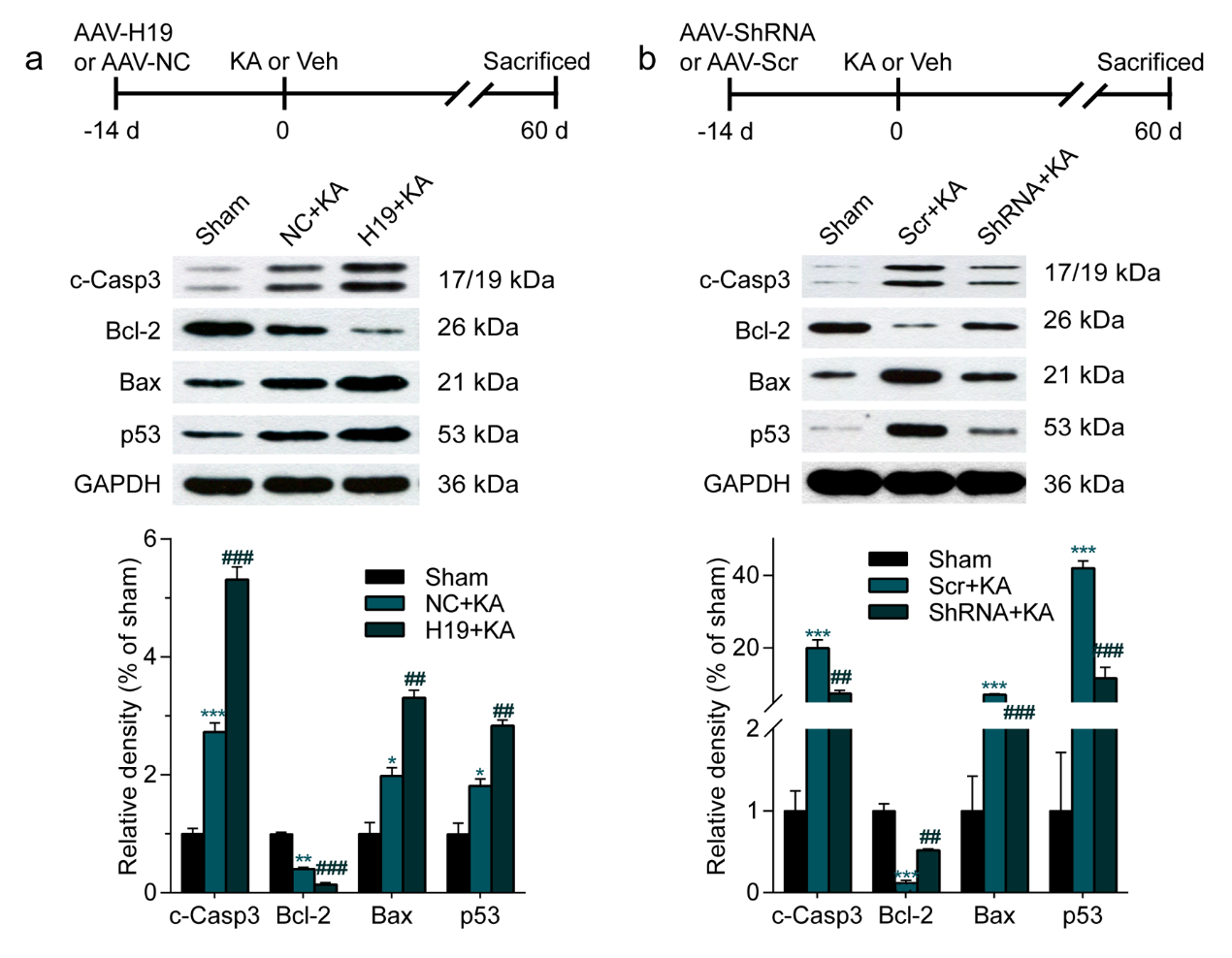


**Supplementary Figure 3** H19 promoted SE-induced hippocampal cell apoptosis *in vivo*. (**a, b**) Top, the time line showing the experimental design. Bottom, quantification of c-Casp3, Bcl-2, Bax and p53 proteins in CA3 subfield of hippocampus from (**a**) H19 overexpression or (**b**) H19 knockdown rats with or without KA treatment for 60 d (n = 3-4). The blots were densitometrically quantified, and the data were normalized to GAPDH blot. All data are shown as mean ± s.e.m. **p* < 0.05, ***p* < 0.01, ****p* < 0.001 versus sham group, #*p* < 0.05, ##*p* < 0.01, ###*p* < 0.001 versus NC+KA or Scr+KA group.

**Supplementary Figure 4**

**
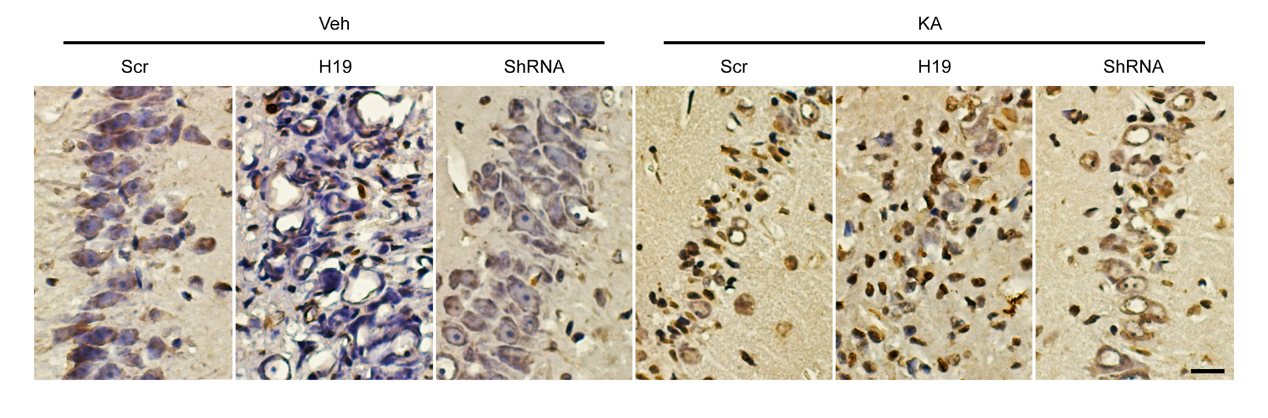
**

**Supplementary Figure 4** Representative photomicrographs of TUNEL-staining in CA3 subfield of hippocampus from H19 overexpression or H19 knockdown rats with or without KA treatment for 7 d. Scale bars = 20 μm

**Supplementary Figure 5**


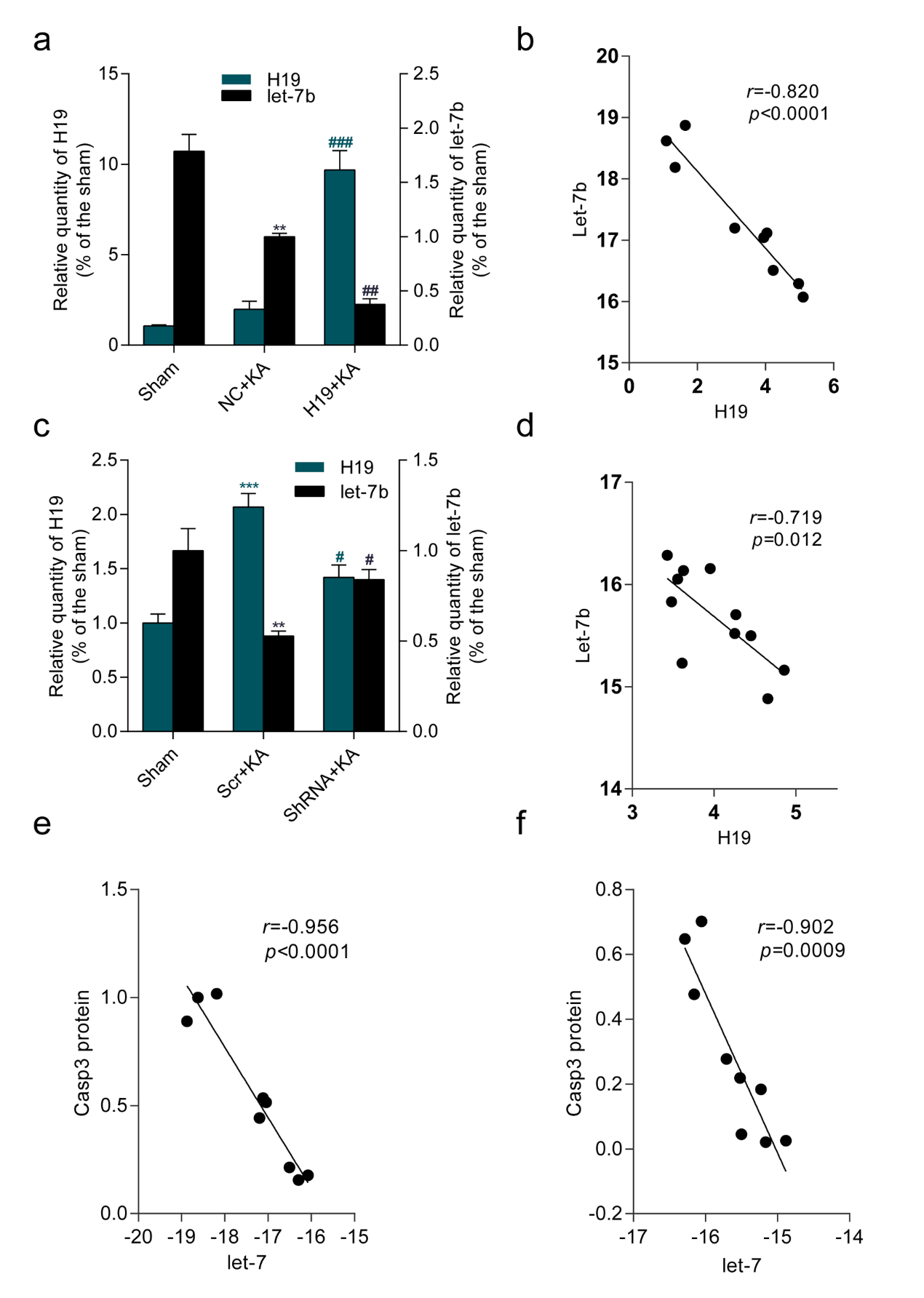


**Supplementary Figure 5** The expression of H19, let-7b, and Casp3 and their correlation in H19 overexpression or H19 knockdown rats. (**a, c**) qPCR quantification of H19 and let-7b levels in CA3 subfield of hippocampus from H19 overexpression (**a**) and H19 knockdown rats (**c**) with or without KA treatment for 60 d (n = 3-5). (**b, d**) Correlations according to Pearson coefficient between H19 and let-7b levels in CA3 subfield of hippocampus from (**b**) H19 overexpression (n = 9) or (**d**) H19 knockdown (n = 11) rats at 60 d after SE. (**e, f**) Correlations according to Pearson coefficient between let-7b and Casp3 protein in CA3 subfield of hippocampus from (**e**) H19 overexpression (n = 9) and (**f**) H19 knockdown (n = 9) rats at 60 d after SE. The relative H19 and Casp3 mRNA levels were normalized to GAPDH and the relative expression of let-7b was normalized to U6. All data are shown as mean ± s.e.m. **p* < 0.05, ***p* < 0.01, ****p* < 0.001 versus NC+Veh or Scr+ Veh group. #*p* < 0.05, ##*p* < 0.01, ###*p* < 0.001 versus NC+KA or Scr+KA group.
